# Tea plant roots respond to aluminum-induced mineral nutrient imbalances by transcriptional regulation of multiple cation and anion transporters

**DOI:** 10.1186/s12870-022-03570-4

**Published:** 2022-04-19

**Authors:** Jing Hao, Anqi Peng, Yingying Li, Hao Zuo, Ping Li, Jinsong Wang, Keke Yu, Chun Liu, Shancen Zhao, Xiaochun Wan, Jon K. Pittman, Jian Zhao

**Affiliations:** 1 State Key Laboratory of Tea Plant Biology and Utilization, Anhui Agricultural University, 130 West Changjiang Road, Hefei, 230036 China; 2 BGI Institute of Applied Agriculture, BGI–Shenzhen, Shenzhen, 518083 China; 3 Department of Earth and Environmental Sciences, Faculty of Science and Engineering, The University of Manchester, M13 9PT, Manchester, UK

**Keywords:** Metal transporter, Transcriptome, Ionome, Ion imbalance, Aluminum tolerance

## Abstract

**Background:**

Tea is one of the most popular non-alcoholic beverages in the world for its flavors and numerous health benefits. The tea tree (*Camellia sinensis* L.) is a well-known aluminum (Al) hyperaccumulator. However, it is not fully understood how tea plants have adapted to tolerate high concentrations of Al, which causes an imbalance of mineral nutrition in the roots.

**Results:**

Here, we combined ionomic and transcriptomic profiling alongside biochemical characterization, to probe the changes of metal nutrients and Al responsive genes in tea roots grown under increasing concentrations of Al. It was found that a low level of Al (~ 0.4 mM) maintains proper nutrient balance, whereas a higher Al concentration (2.5 mM) compromised tea plants by altering micro- and macro-nutrient accumulation into roots, including a decrease in calcium (Ca), manganese (Mn), and magnesium (Mg) and an increase in iron (Fe), which corresponded with oxidative stress, cellular damage, and retarded root growth. Transcriptome analysis revealed more than 1000 transporter genes that were significantly changed in expression upon Al exposure compared to control (no Al) treatments. These included transporters related to Ca and Fe uptake and translocation, while genes required for N, P, and S nutrition in roots did not significantly alter. Transporters related to organic acid secretion, together with other putative Al-tolerance genes also significantly changed in response to Al. Two of these transporters, *CsALMT1* and *CsALS8*, were functionally tested by yeast heterologous expression and confirmed to provide Al tolerance.

**Conclusion:**

This study shows that tea plant roots respond to high Al-induced mineral nutrient imbalances by transcriptional regulation of both cation and anion transporters, and therefore provides new insights into Al tolerance mechanism of tea plants. The altered transporter gene expression profiles partly explain the imbalanced metal ion accumulation that occurred in the Al-stressed roots, while increases to organic acid and Al tolerance gene expression partly explains the ability of tea plants to be able to grow in high Al containing soils. The improved transcriptomic understanding of Al exposure gained here has highlighted potential gene targets for breeding or genetic engineering approaches to develop safer tea products.

**Supplementary Information:**

The online version contains supplementary material available at 10.1186/s12870-022-03570-4.

## Background

Tea is one of the most popular non-alcoholic beverages, consumed by over two-thirds of the world’s population due to its health-beneficial, refreshing and pleasant stimulant effects. Because tea made from the fresh buds and leaves of tea plants (*Camellia sinensis* L.) contains various nutritional trace elements, drinking tea provides intake of many essential macro- and micro-nutrients for humans [1–4]. Meanwhile, environmental exposure of tea plants to metal pollutants may inhibit growth and therefore the yield of tea, and may cause the accumulation of significant amounts of non-essential and potentially toxic metals in tea leaves, which is a public health concern for tea products [2, 3]. For example, trace elements including, aluminum (Al), arsenic (As), cadmium (Cd), copper (Cu), fluoride (F), manganese (Mn), chromium (Cr), and nickel (Ni) have been reported in different tea infusions, and in some cases metal concentrations exceed the maximum permissible limits [3].

Tea plants are perennial evergreen woody trees or shrubs, which are widely grown in subtropical mountainous regions of southern China, the Assam area in India, and other parts of the world [5]. Many tropical and sub-tropical plant species such as tea plants grow in acidic soils that are prevalent in these regions. Metals including Al are more bioavailable in acidic soils leading to Al^3+^ toxicity, which is one of the major factors limiting crop production on acid soils [6]. Elevated Al (as Al^3+^) causes rhizotoxicity symptoms on plants: root elongation is severely inhibited, which could result from the binding of Al to multiple cellular targets and subsequent disruption of the functions of the cell wall, cell membrane, as well as various proteins and enzymes, DNA replication, and other cellular processes [6–10]. Most often, plants grown on acidic soils also suffer from other alterations to essential mineral nutrition that is influenced indirectly by high Al and H^+^ availability. Furthermore, the availability of Al may alter the uptake characteristics of other non-essential but toxic metals such as Cd. Although many metals such as iron (Fe), Mn, Cu, and zinc (Zn) are essential trace micronutrients, excessive accumulation of these metals can be toxic, while deficiencies of micronutrients and macronutrients, such as nitrogen (N), phosphorus (P), sulfur (S), calcium (Ca), magnesium (Mg), and potassium (K) are deleterious to plants [6, 7]. These indirect effects of Al and H^+^ toxicity provide further plant stress, by evoking subsequent oxidative stresses and triggering cell death [8, 11]. While most major crops display significant sensitivity to acidic soils, tea plants can grow normally on most acidic soils containing higher concentrations of Al, without any obvious rhizotoxicity symptoms [4, 12, 13]. So far, at least two broad resistance mechanisms involving external or internal detoxification have been proposed to explain most plant adaptive responses to Al toxicity [6]. For external exclusion mechanisms, many plants secrete compounds including organic acids from their root tips to chelate Al and avoid Al toxicity at the cell wall or to prevent Al entering into growing root tip cells, thus providing a certain degree of Al resistance [14–16]. Organic acids are also used for internal tolerance mechanisms, whereby Al-organic acid complexes accumulate in the root symplasm and then facilitate the partitioning of Al into older shoot tissues for storage in a non-toxic form. In addition, tea plants are Al hyperaccumulators that can tolerate these acidic, high Al conditions due to their ability to accumulate high amounts of Al, such that Al concentrations in older leaves are typically 100-fold higher (e.g., ~ 30,000 mg kg^− 1^) than in leaves of two of the most Al-tolerant monocot plants, rice and common buckwheat, and which in turn are approximately 2–7 times more Al-tolerant than wheat, sorghum, or maize [10, 12, 13]. Tea plants are able to hyperaccumulate high concentrations of Al in part due to the ability to maintain and even enhance root growth in the presence of Al, to allow mobile forms of Al to be translocated into aerial tissues, and having enhanced leaf anti-oxidant processes [9, 10, 12]. However, the accumulation of Al, F, and other metals in tea plant leaves [1, 2] introduces food safety issues and health concerns for drinking tea or consumption of tea products. While some of the physiological and biochemical details of Al tolerance and accumulation mechanisms by tea plants are known, there are still gaps in knowledge particularly with regard to the identification of genetic details of these processes. Therefore research to investigate the uptake, transport, and distribution mechanisms of Al, as well as other Al affected metals is of interest in order to potentially breed low Al-accumulating and reduced metal containing tea plant varieties for safer drinks. Understanding the plant’s Al tolerance mechanisms is also of interest to identify new molecular tools to engineer Al-tolerance to other major crops and to expand their cultivation on acidic soils.

The aim of this study was to explore the Al accumulation as well as Al detoxification mechanisms of tea plants, and the consequential mineral nutritional changes induced by Al exposure, by integrating transcriptomic and ionomic profiling, and biochemical analyses on seedlings grown hydroponically under various Al stress conditions in acidic solutions. We observed imbalanced accumulation of various metal ions in roots, and altered expression of many metal ions and organic acid transporter genes, as well as genes encoding oxidative stress-related enzymes and other putative Al tolerance genes. Since these membrane transporters play essential roles in metal ion uptake, translocation, distribution, and homeostasis, their expression changes are implicated in the tea plant’s adaptive responses to Al and H^+^ stresses.

## Results

### Effects of Al exposure on tea seedling root growth and Al accumulation

**Fig. 1:**
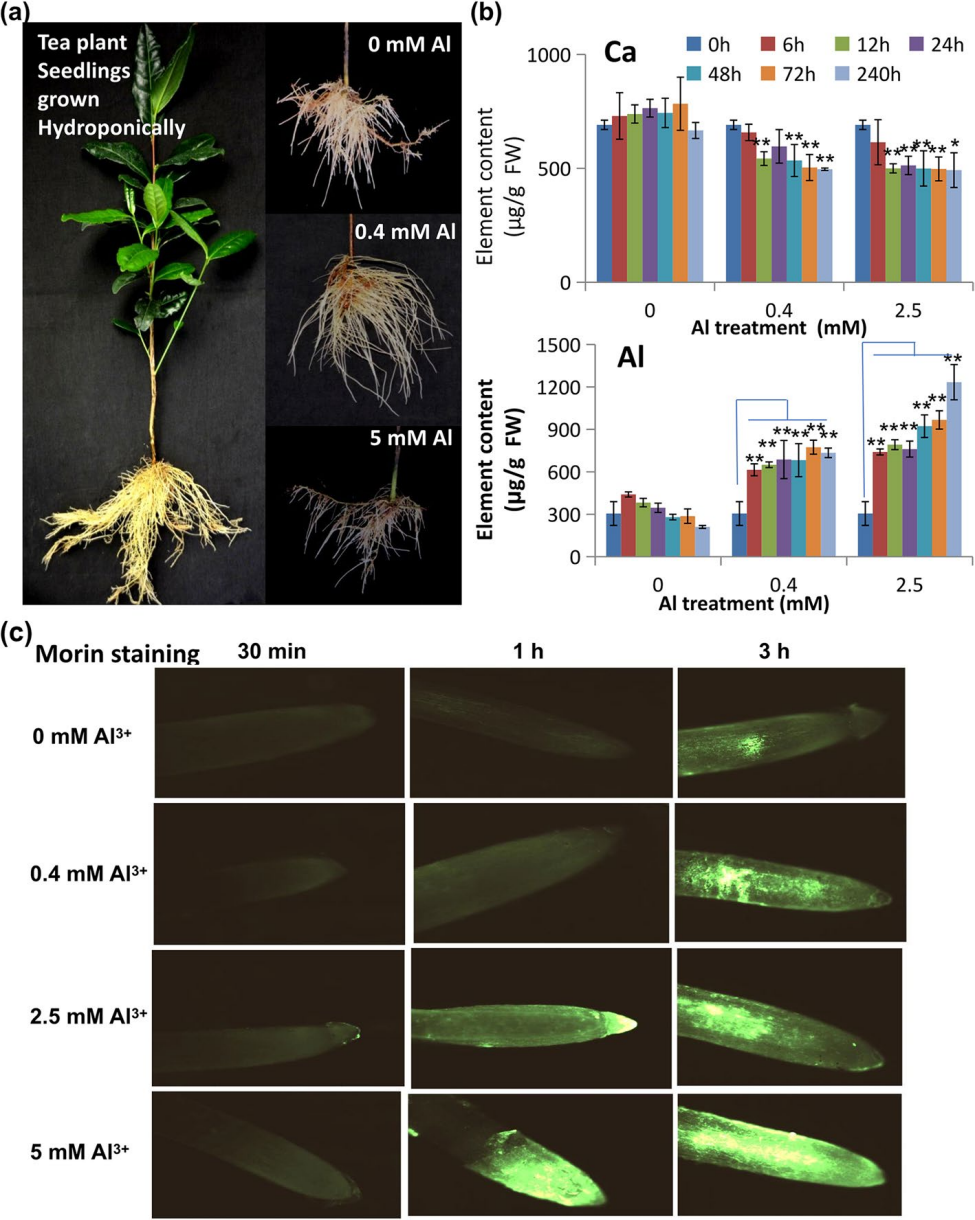
Effects of Al stress on root growth of hydroponically grown tea plants. **a** Two-year old tea plant seedlings of the same genetic background were cultivated hydroponically in SK-medium containing 0.4 mM Al_2_(SO_4_)_3_. The seedlings were treated 0 mM Al or 5 mM Al for the effects of growth. **b** Contents of Ca and Mg in the roots of tea plant seedlings under 0, 0.4, and 2.5 mM Al stress for different times measured by ICP-MS. **c** Imaging of Al accumulation in root tips of tea plant seedlings. The Al-specific fluorescence dye morin was used for imaging. The representative photos shown are from three independent experiments. Data are presented as means ± SD from three independent experiments and statistical significance is based on Student’s t-test (*P < 0.05; ** *P* < 0.001) compared with controls

To gain further understanding of the Al-tolerance mechanisms of tea plants, we treated tea seedlings with different concentrations of Al (0, 0.4, and 2.5 mM, pH 4.3) in modified Shigeki Konishi nutrient solution (SK-solution). Standard SK-solution contains 0.4 mM Al_2_(SO_4_)_3_ since previous studies have demonstrated that Al at this concentration effectively promotes tea seedling root growth and development in contrast to an absence of Al [12, 17]. In the weekly replaced SK-solutions with consistent pH 4.3, we determined Al^3+^ activity at 0.4 mM Al equated to approximately 200 μM Al^3+^ [9, 12]. When tea seedlings were grown hydroponically in SK-solution without Al or containing higher (> 0.4 mM) Al concentrations, the roots displayed clear differences: roots in 0 mM Al-solution were shorter and grew less strongly than those from the 0.4 mM-solution, which showed the healthiest growth (Fig. 1A). But roots from the 2.5 mM Al-solution showed substantially worse root growth (Fig. 1A). We also observed that roots grown in SK-solution containing 5 to 10 mM Al displayed significantly worse growth performance: roots became brown and root growth was severally inhibited (Fig. 1A; data not shown), consistent with other reports [12, 17]. Quantification of root elongation found that seedlings grown in 2.5 mM Al were not significantly changed until 10 days of treatment, and then after a further 5 days of growth there was 16% root inhibition compared to the seedlings grown in 0.4 mM Al.

The root stress symptoms observed were coincident with significantly increased Al accumulation over time as external Al concentration was increased (Fig. 1B). Al content in roots of seedlings treated with no added Al showed no significant change over time and fluctuated slightly at a baseline concentration of approximately 300 mg kg^− 1^. However, the Al content rapidly increased to 600–800 mg kg^− 1^, even after just 6 h of cultivation in 0.4 mM Al-SK solution, but then with very little change over the rest of the 10 day incubation period. In 2.5 mM Al-SK solution, Al content in roots also rapidly increased to 700–800 mg kg^− 1^ after 6 h, and eventually reached a high of approximately 1200 mg kg^− 1^ after 10 days of incubation (Fig. 1B). Root Al accumulation was further examined using the fluorescent Al-sensitive morin dye. Root tip Al accumulation was clearly observed after 3 h exposure within the stele of the root, with a low level of staining for the 0.4 mM Al treatment but very intense staining for the 5 mM Al treatment (Fig. 1C). At earlier time points (1 h) only the 5 mM Al treatment showed strong staining, while some extreme tip staining was consistently observed for the 2.5 mM Al treated roots. No staining was observed for any treatments after just 30 min.

### Changes in metal contents in roots of tea seedlings upon Al stress

**Fig. 2:**
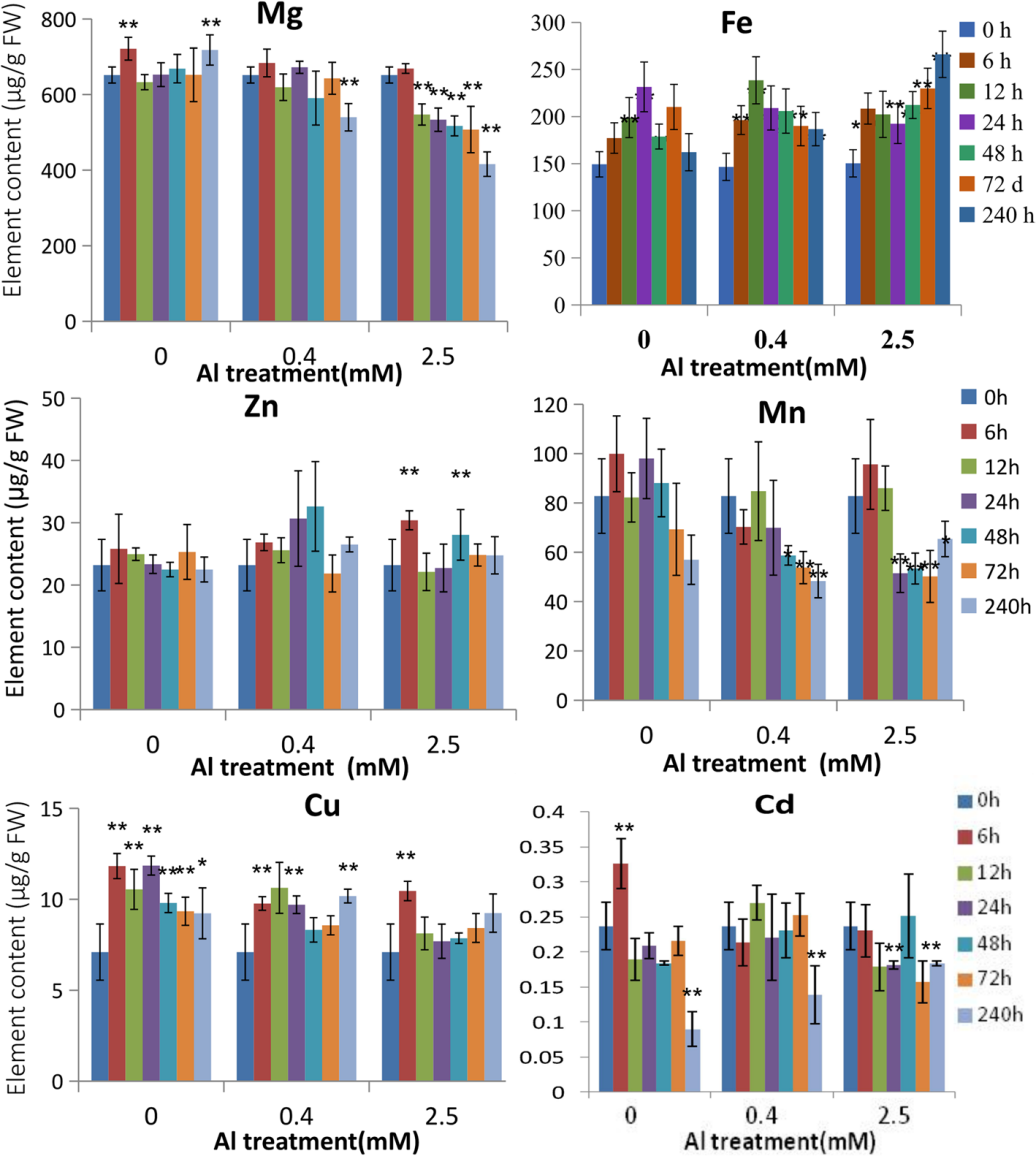
Effects of Al stress on the metal content in roots of hydroponically grown tea plants. Metal element accumulation (Mg, Fe, Zn, Mn, Cu and Cd) in roots of two-year-old tea seedlings grown hydroponically in SK medium containing 0.4 mM Al, and exposed to a series of Al concentrations (0, 0.4, 2.5 mM Al) for different times, then measured by ICP-MS. Data are presented as means ± SD from three independent experiments and statistical significance is based on Student’s t-test (*P < 0.05; ** P < 0.001) compared with controls

Macronutrients such as K, Ca and Mg, and some transition metal micronutrients such as Fe, Cu, Mn, and Zn are essential mineral nutrients for normal plant growth and development, but they also can be toxic to plants when present in excess [18]. To understand how high Al and acidic conditions effects mineral nutrition in tea plant roots, metal elements were measured in the roots from seedlings grown in 0, 0.4, and 2.5 mM Al over a 10 day period. Overall, we observed significantly reduced Ca, Mg and Mn root content in the highest Al treatments, and fluctuations in the root concentrations of Fe, Zn, and Cu (Fig. 2). Ca and Mg content showed no significant change over time in the absence of Al (0 mM treatment), but there was a decrease over time in Ca content in the 0.4 and 2.5 mM Al treatments (Fig. 1B). Likewise, Mg concentration of roots steadily decreased over time, particularly in the 2.5 mM Al treatment where Mg content dropped quickly by 12 h after treatment and continued to decrease to ~ 400 mg kg^− 1^ by day-10 of the treatment period. Mn content also decreased in response to Al. Fe contents in roots fluctuated in larger ranges between 140 to over 300 mg kg^− 1^ with both 0.4 and 2.5 mM Al treatments inducing rapid increases in Fe content after 6 h. The exposure of Al gave a much smaller and more variable response to Cu and Zn contents. For example, some increases in Zn content were observed in the 0.4 and 2.5 mM Al treatments but only a few time points were significantly higher than in the 0 mM Al treatment roots (Fig. 2).

Cd, like Al, is a non-essential and very toxic metal which is able to easily accumulate into plant roots. It was also examined whether Al exposure provides any protection against Cd accumulation by exposing plants to the three Al treatments (0 mM, 0.4 mM and 2.5 mM) and then measuring Cd content. In tea seedlings that were exposed to 0 mM Al treatment, an initial rapid increase in Cd content after 6 h was followed by a decline in root Cd content after 12 h, with a significant drop after 10 days, possibly due to a transfer of Cd into shoot tissues and/or efflux out of the roots (Fig. 2). In response to Al treatment, the initial Cd accumulation into the root was mostly inhibited but more Cd was retained in the roots by 10 d, particularly in the 2.5 mM Al treatment.

### Global gene expression changes in roots upon Al exposure

To understand the molecular mechanisms taking place in the Al-stressed roots in more detail, an RNA-Seq transcriptome analysis was performed. About 45,737 transcript contigs were assembled and 86% were able to be matched to the *C. sinensis var. sinensis* genome. Compared with the non-Al (0 mM) control treatment, the 2.5 mM Al treatment resulted in 2617 up-regulated and 1544 down-regulated genes at 12 h, 1165 up-regulated and 1304 down-regulated genes at 24 h, and 1611 up-regulated and 1704 down-regulated genes at 48 h. However, compared with the 0.4 mM Al treatment, the 2.5 mM Al treatment resulted in 1766 up-regulated and 1768 down-regulated genes at 12 h, 1859 up-regulated and 1329 down-regulated genes at 24 h, and 1719 up-regulated and 1484 down-regulated genes at 48 h. Genes assigned to four categories, signal transduction, environmental adaptation, immune system and sensory system, were over-represented in Al-stressed roots. These included a large number of putative membrane proteins and transporters, including essential and non-essential metal transporters, and organic acid transporters, as well as genes involved in oxidative stress response. Phylogenetic analyses, in comparison with *Arabidopsis thaliana* genes, were performed to group tea genes into specified gene families (Table S1-S9, Fig. S1–24). In addition, the plant tissue expression patterns of each of these identified genes were determined (Table S1-S9, Fig. S1–24).

### Al stress-induced changes in ca and mg transporter genes

**Fig. 3:**
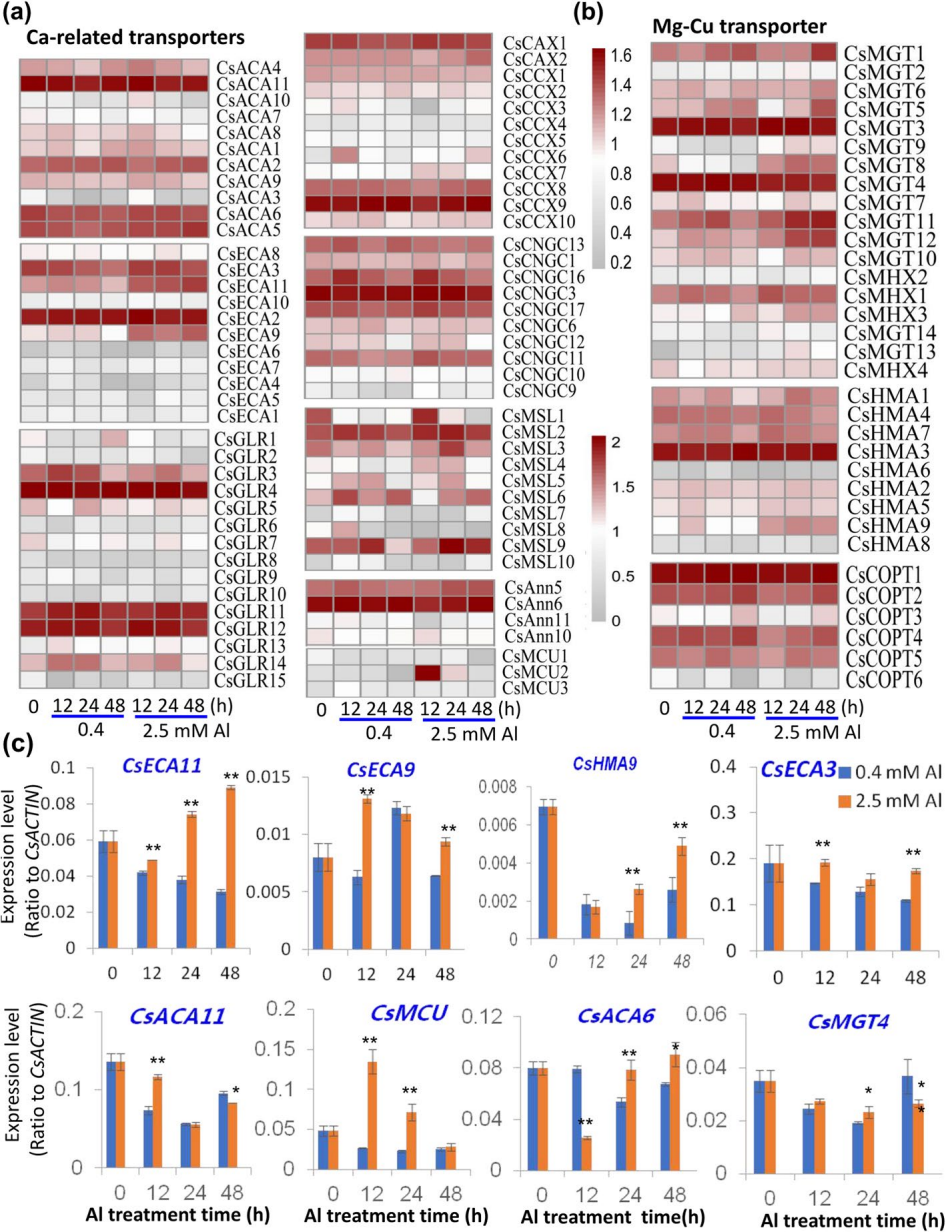
Effects of Al stress on the expression of transporter genes involved in root transport of Ca, Mg, and Cu and other divalent cations. **a** Heatmap analysis of expression patterns of genes encoding various transporters in roots in response to 0.4 mM and 2.5 mM Al stresses at different time points. The transporters include Type IIB Ca^2+^-ATPases (*CsACA*), Type IIA ER-Ca^2+^-ATPases (*CsECA*), Ionotropic glutamate receptor (*CsGLR*), Ca^2+^/H^+^ antiporter (*CsCAX*), Cyclic nucleotide-gated channels (*CsCNGC*s), Mechanosensitive ion channel like (*CsMSL*), Annexins (*CsAnn*), Mitochondria calcium uniporter (*CsMCU*). **b** Heatmap analysis of expression patterns of genes encoding tonoplast Mg/H transporter (*CsMHX*), Magnesium transporter (*CsMGT*), Heavy metal H-ATPase (*CsHMA*), and Copper transporter (*CsCOPT*). **c** qRT-PCR verification of expression patterns of several key transporter genes in roots in response to 0.4 mM and 2.5 mM Al. The heatmaps were made with transcriptome data normalized as Log_10_(1 + log (1 + FPKM)). MeV software was used to depict transcript levels. qRT-PCR data are presented as means ± SD from three independent experiments and statistical significance is based on Student’s t-test (*P < 0.05; ** *P* < 0.001) compared with controls

Two major essential nutrients Mg and Ca, were significantly reduced in tea plant roots in response to high concentration of Al. Therefore, it was relevant to further examine the gene candidates known to be involved in plant Ca^2+^ and Mg^2+^ transport and homeostasis. In addition to the RNA-Seq data, validation of selected genes was performed by RT-PCR. The cellular homeostasis of Ca^2+^ in plant cells is enabled by membrane transporters at the plasma membrane and at endomembranes, including various Ca^2+^-permeable ion channels for Ca^2+^ influx into the cytosol [19]. and active Ca^2+^ efflux transporters, which can include the endoplasmic reticulum-type Ca^2+^-ATPase (ECA) and autoinhibited Ca^2+^-ATPase (ACA), the Ca^2+^/H^+^ exchanger (CAX), and the mitochondrial Ca^2+^ uniporter (MCU) proteins [20]. Ca^2+^ influx is mediated by several families of selective and non-selective ion permeable channels, including cyclic nucleotide-gated channels (CNGCs), ionotropic glutamate-like receptors (GLR), two-pore channel 1 (TPC1), annexins, and mechanosensitive-like (MSL) ion channels, although it is important to highlight that many of these channels can also mediate the movement of other ions in addition to Ca^2+^ (Table S1, Fig. S1-S7) [21]. The RNA-Seq data as shown in the heatmaps indicated that many of the Ca^2+^ permeable channel genes were down-regulated in response to 0.4 or 2.5 mM Al, including the GLR genes TEA014867 (*CsGLR1*) and TEA006325 (*CsGLR15*), and the MSL genes TEA001140 (*CsMSL1*) and TEA019176 (*CsMSL8*), while TEA024970 (*CsGLR13*) was up-regulated in Al treated roots at 12 h and 24 h (Table S1, Fig. S1-S7). In contrast, some of the transcripts encoding proteins responsible for moving Ca^2+^ into internal organelles, including a putative MCU gene TEA000129 (*CsMCU2*) and two ECAs TEA015853 (*CsECA9*) and TEA011305.1 (*CsECA11*), showed up-regulation by 2 to 8-fold over the 3 time points examined in response to excess (2.5 mM) Al (Fig. 3A). The ACA genes TEA023534 (*CsACA6*) and TEA026012 (*CsACA11*) were down-regulated in response to both Al treatments in comparison to no Al. There was also evidence of Al-responsive reduced transcript abundance for TEA008626 (*CsACA3*). However, CAX genes including TEA007963 (*CsCAX1*) and TEA000101 (*CsCAX2*) did not show any significant expression change in response to Al (Fig. 3A, Table S1, Fig. S1-S7).

The CCX gene family has been proposed to play a role in the transport of various ions, with some *A. thaliana* members such as AtCCX3 and AtCCX4 implicated in K^+^ and possibly Na^+^ transport, while AtCCX1 and AtCCX2 are likely to function as Ca^2+^ transporters [22, 23]. One of the tea plant CCX genes TEA032754 (*CsCCX3*), was markedly down-regulated by Al treatment, while TEA021659 (*CsCCX6*) was highly up-regulated by between 2 to 8-fold in response to 0.4 mM and 2.5 mM Al treatment (Fig. 3A, Table S1, Fig. S1-S7).

Mg plays a positive role in plant resistance to Al stress [24, 25]. Two classes of Mg transporters have been well studied in plants, the Mg^2+^/H^+^ exchangers (MHX) and the Mg^2+^ transporters (MGT). AtMHX is a tonoplast protein mediating transport of Mg^2+^ into the vacuole in a proton-dependent manner [26, 27]. *A. thaliana* MGTs including AtMGT1 and AtMGT10 are plasma membrane high affinity Mg^2+^ transporters that mediate Mg uptake into roots and also have low affinity to other cations including Fe^2+^, Mn^2+^ and Cu^2+^, and yet are also highly sensitive to Al stress [24, 26]. Some MGTs even can transport Al [27]. From the 14 putative MGT genes in tea, TEA005607 (*CsMGT5*), TEA011435 (*CsMGT8*), TEA015654 (*CsMGT11*), and TEA015667 (*CsMGT12*) were up-regulated by approximately 2 to 3.2-fold in response to 2.5 mM Al stress, while TEA030537 (*CsMGT13*) was up-regulated (between 2.6 and 9-fold) following both Al treatments (Fig. 3B). In contrast, TEA010637 (*CsMGT4*), TEA011435 (*CsMGT8*) and TEA011455 (*CsMGT9*) showed down-regulation in response to 0.4 mM Al, with *CsMGT8* and *CsMGT9* giving markedly higher transcript abundance in the 2.5 mM treatments compared to the 0.4 mM treatments, and the representative gene *CsMGT4* was verified with qRT-PCR. (Fig. 3B,C, Table S2, Fig. S8). Only the MHX gene TEA032978 (*CsMHX3*) was slightly up-regulated in response to 2.5 mM Al (Fig. 3B).

HMAs belong to the P-type ATPase superfamily, which is further classified into several sub-families, according to their transport specificity [28]. The P1B HMA sub-family can either mediate the transport of divalent Zn^2+^/Co^2+^/Cd^2+^/Pb^2+^ (including AtHMA1 to 4) or monovalent Cu^+^/Ag^+^ (including AtHMA5 to 8) [18, 29]. Most tea HMAs showed just moderate responses to Al exposure. Those most closely related to AtHMA1 to 4 and therefore putative Zn^2+^ and Cd^2+^ efflux transporters, including TEA019557 (*CsHMA1*) and TEA018983 (*CsHMA2*), were moderately increased (but less than 2-fold) in response to 2.5 mM Al (Fig. 3B, Table S4, Fig. S16). Likewise, those more closely related to AtHMA5 to 8 and therefore putative Cu^+^ efflux transporters, including TEA018281 (*CsHMA3*), TEA000616 (*CsHMA7*), TEA000615 (*CsHMA9*) were also moderately up-regulated in response to 2.5 mM Al treatment (Fig. 3B), and data was validated by qRT-PCR for representative gene *CsHMA9* (Fig. 3C). The Cu influx transporter (COPT) family are high affinity plasma membrane Cu transporters [30]. Three of the tea COPT genes were highly expressed in the roots: TEA022572 (*CsCOPT1*), TEA030188 (*CsCOPT2*) and TEA024734 (*CsCOPT4*). These all show induced expression of approximately 1.4-fold following by 48 h following 0.4 mM Al exposure (Fig. 3B, Table S3, Fig. S16-S17).

### Altered regulation of Fe transport and homeostasis genes in tea plant root by Al stress

**Fig. 4:**
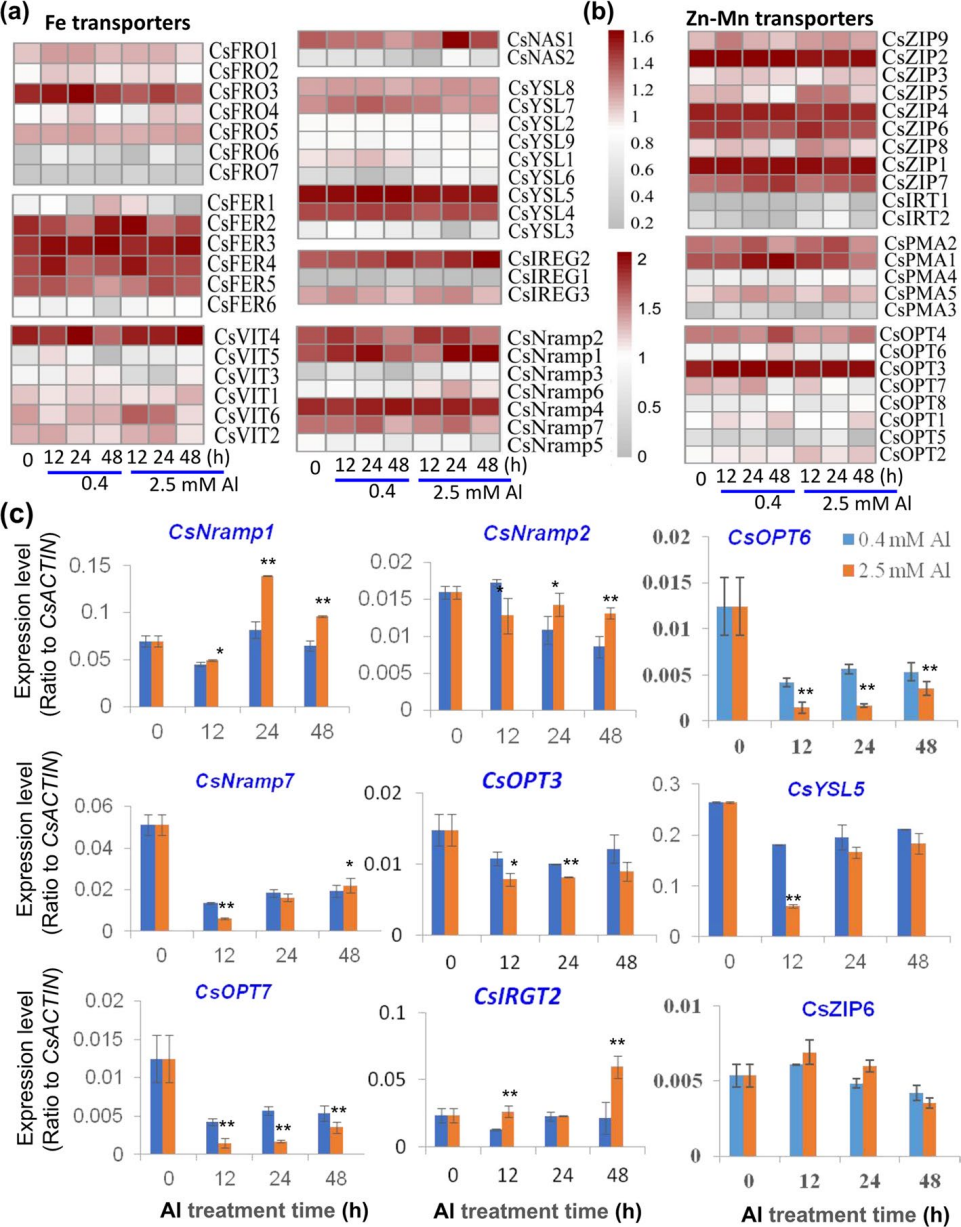
Effects of Al stress on transporter genes involved in the transport of Fe, Zn, Cu, and other metals. **a** Expression patterns for genes encoding transporters or enzymes involved in transport of Fe or Fe-organic acid complex across various membranes, including Ferric reductase oxidase (*CsFRO*), Fe-binding proteins ferritin (*CsFER*), vacuolar iron transporters (*CsVIT*s), nicotianamine synthase (*CsNAS*), yellow-stripe like (*CsYSL*) transporters, and IRON REGULATED/Ferroportin (*CsIREG/Ferroportin*). **b** Expression patterns for other genes encoding multi-function transporters, such as NRAMP transporters, ZRT/IRT-like proteins (*CsZIP*s), plasma membrane ATPases (*CsPMA*s), oligopeptide transporter (*CsOPT*s). **c** qRT-PCR verification of expression patterns of key genes involved in metal ion transport in roots in response to 0.4 mM and 2.5 mM Al. The heatmaps were made with transcriptome data normalized as Log_10_(1 + log (1 + FPKM)). MeV software was used to depict transcript levels. qRT-PCR data are presented as means ± SD from three independent experiments and statistical significance is based on Student’s t-test (*P < 0.05; ** P < 0.001) compared with controls

Fe concentrations were significantly increased by Al stress in tea plant roots. This correlated with observed alteration in the expression of Fe metabolism and transport related genes. For most dicots, the ferric reductase oxidase (FRO) reduces Fe^3+^ to Fe^2+^, which can then be transported into the root epidermis by the divalent metal transporter IRT1, which is a member of the ZRT/IRT-like protein (ZIP) family of transporter. Vacuolar iron transporter (VIT) proteins at the tonoplast can in turn store labile Fe^2+^ into vacuolar compartments, such as performed by *A. thaliana* AtVIT1, while some members of the natural resistance-associated macrophage protein (NRAMP) family can mediate Fe release from the vacuole [31, 32]. Of the seven putative FRO genes, TEA033298 (*CsFRO6*) expression increased over 2-fold in response to both 0.4 and 2.5 mM Al treatments (Fig. 4A). Expression of a putative tea *CsIRT1* gene (TEA023203) was undetectable in control or 0.4 mM Al treated roots, but low-level expression was observed following 2.5 mM Al treatment (Fig. 4A). Six VIT genes were found within the tea root transcriptome (Table S3, Fig. S9-S12). In comparison to the control condition, TEA021515 (*CsVIT3*) was down-regulated by 2-fold and 4-fold at 12 h and 24 h, respectively after 2.5 mM Al treatment, while the transcript that is most closely related to AtVIT1, TEA015983 (*CsVIT6*), was increased by nearly 3-fold at 12 h under 2.5 mM Al treatment in comparison to the 0.4 mM Al treatment (Fig. 4A). NRAMPs are broad substrate transporters including AtNRAMP3 and AtNRAMP4, which are associated with Fe^2+^ and Mn^2+^ transport [32, 33]. TEA011223 (*CsNRAMP2*) is most closely related to these genes and was slightly down-regulated in response to 0.4 mM Al (Fig. 4A, Table S3, Fig. S12). Other transport proteins that are associated with Fe homeostasis are members of the oligopeptide transporter (OPT) family, which include the Yellow Stripe-like (YSL) proteins that can mediate the transport of Fe chelate complexes such as Fe bound to phytosiderophores (PS) or nicotianamine (NA) [34–36]. The IRON REGULATED (IREG) / ferroportin (FPN) proteins are efflux transporters, such as AtFPN2, a vacuolar membrane protein that is expressed during Fe deficiency [37], while soybean and buckwheat GmIREG3 and FeIREG3 are tonoplast transporters that contribute to Al resistance in plants [38]. OPT genes, such as TEA015243 (*CsOPT3*), TEA016756 (*CsOPT6*) and TEA021263 (*CsOPT7*) were down-regulated in roots under 0.4 mM and 2.5 mM Al treatment (Fig. 3A, Table S3, Fig. S9-S12). Most of the YSL genes examined did not show a clear response to Al exposure, with the exception of TEA025648 (*CsYSL6*) that was induced by 2.5 mM Al, while TEA019706 (*CsYSL1*) and TEA018013 (*CsYSL3*) were down-regulated by 2.5 mM Al treatment compared with the control (Fig. 4B). The IREG TEA008315 (*CsIREG2*) was up-regulated by 2.5 mM Al stress at 48 h after treatment (Fig. 4B).

NA is synthesized in higher plants by NA synthase (NAS). NA complexes with Fe^2+^ and Fe^3+^, and other metal ions, such as Cu^2+^, Ni^2+^, Co^2+^, Zn^2+^, and Mn^2+^ to facilitate transport into cells via YSL transporters and the long-distance translocation of mineral nutrients from roots to shoots or vice versa. Among the two NAS genes, TEA020761 (*CsNAS1*) was up-regulated in response to 2.5 mM Al at 24 h (Fig. 4B), suggesting the potential for enhanced Fe-NA complexes for translocation within the tea plant. The Fe-binding protein ferritin (FER) also plays an important role in plant Fe homeostasis [39]. Altered transcript abundance was seen for most of the *CsFER* genes. TEA016450 (*CsFER1*), TEA002014 (*CsFER3*) and TEA026599 (*CsFER4*) were up-regulated following 0.4 mM Al treatment, while TEA016447 (*CsFER2*) showed increased abundance in 2.5 mM Al (Fig. 4B, Table S3, Fig. S15). Some of these significantly up-regulated genes as mentioned above were selected for being verified with qRT-PCR (Fig. 4C).

### Changes in expression of metal transporter genes for trace metals

Some subtle but significant changes in the content of Cu, Mn and Zn, as well as the non-essential toxin Cd were observed in tea roots in response to Al exposure. These included slightly reduced Cu and Mn content and slightly increased Zn and Cd content, which corresponded with changes in expression of various metal transporters. Many of the transporter classes described above, including non-selective cation channels, ZIPs, NRAMPs, and OPTs, are all broad substrate transporters that can mediate accumulation and cellular distribution of these and other metals [18, 40, 41]. Of the various ZIP genes identified, many showed transient fluctuation in expression in response to Al stress. TEA032109 (*CsZIP5*) and TEA028928 (*CsZIP8*) were up-regulated by 2.5 mM Al treatment by over 2-fold while TEA028921 (*CsZIP7*) was up-regulated in 0.4 mM Al treatment at 48 h compared with the control (Fig. 4B). Tea NRAMP transporter genes TEA017264 (*CsNRAMP1*), TEA002435 (*CsNRAMP3*) and TEA009385 (*CsNRAMP6*), which are all specifically expressed in root tissue (Table S3, Fig. S12-S13), were all were up-regulated substantially in response to Al. Based on the phylogenetic analysis, these three genes are most closely related to *OsNRAMP4* from rice, which is a root plasma membrane transporter that can provide Al^3+^ accumulation into root tip cells but does not appear to transport other cations including Fe^2+^, Mn^2+^ or Cd^2+^ [42]. These tea NRAMPs are also closely grouped with AtNRAMP1, which is a plasma membrane Mn^2+^ and Fe^2+^ transporter [18, 29].

### Antioxidant and oxidative stress under Al^3+^/H^+^ rhizotoxicity

**Fig. 5:**
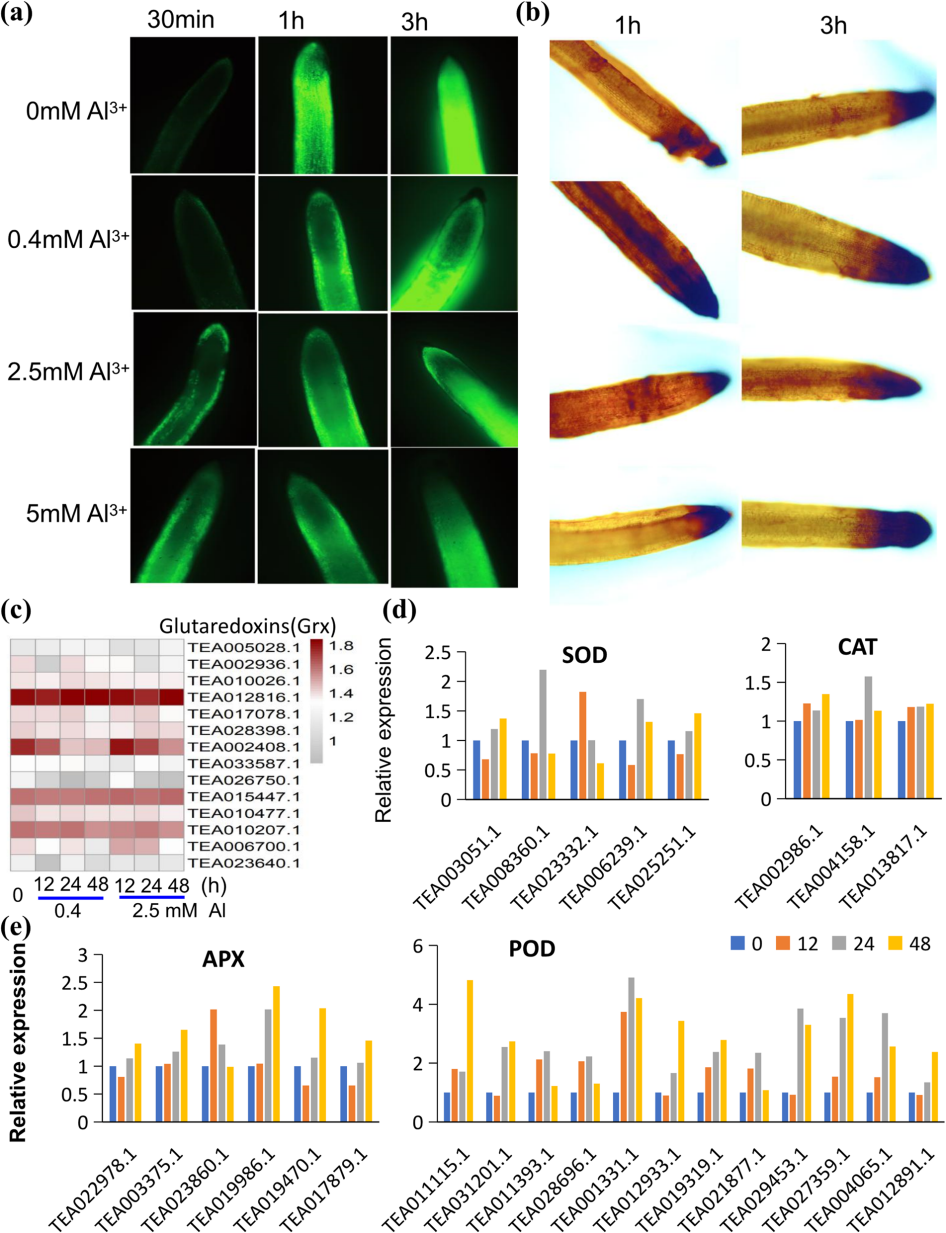
Oxidative stress in tea plant roots upon Al stress. **a**, **b** 2,7-DCFDA staining of ROS (a) and DAB staining of H_2_O_2_ (**b**) in tea seedling roots in response to 0, 0.4, 2.5, and 5 mM Al_2_(SO4)_3_. The root tips were stained with 2,7-DCFDA or 3,3-diaminobezidin (DAB) in 10 mM Tris-HCl (pH 7.6) and then imaged using a fluorescence microscope. **c** Heatmap analysis of expression of glutaredoxin (*CsGRX*) genes in tea seedling roots in response to Al stress. **d** Analysis of expression patterns of superoxide dismutase (*CsSOD*) and catalase (*CsCAT*) genes in tea seedling roots in response to Al stress. The data are the ratio of transcriptome data at different treatment times. **e** Analysis of expression patterns of ascorbate peroxidase (*CsAPX*) and peroxidase (*CsPOD*) genes in tea seedling roots in response to Al stress. Data are presented as averages of calculated FPKM readings and representative fluorescence microscopy images are shown. All results were from three independent experiments

One of the most significant changes in most plant roots under Al stress is oxidative damage caused by Al toxicity and the Al-induced accumulation of metal ions such as Fe^2+^ [11]. We therefore used the 2,7-DCFDA reporter to monitor for increasing reactive oxygen species (ROS) in roots. While fluorescence was observed in all roots including the control treatment without Al after the 1 h time point, fluorescence at 30 min was only seen in response to Al exposure and fluorescent intensities increased in response to increasing Al concentrations (Fig. 5A). It was also observed that the epidermal cells and root elongation zones showed more fluorescence than other parts of the roots. We also determined H_2_O_2_ generation and localization using DAB staining (Fig. 5B), which showed that root tips were the regions that produced more H_2_O_2_, which accumulated over the time of Al exposure and in response to increasing concentration of Al. Glutaredoxin (GRX) and thioredoxin (TRX) proteins play important roles in glutathione metabolism, which is important for heavy metal-induced oxidative stress tolerance [43]. Among 14 GRX genes tested, a few showed increased expression levels under Al exposure, including TEA005028 (*CsGRX1*), TEA012816 (*CsGRX8*), TEA015447 (*CsGRX20*), and TEA006700 (*CsGRX28*), while others including TEA002408 (*CsGRX11*) and TEA026750 (*CsGRX18*) were decreased by Al (Fig. 5C). Many TRX genes were mostly up-regulated by Al stress, suggesting significant changes in cellular redox status (Table S5, Fig. S18-S20).

Several genes encoding ROS scavenging enzymes were examined. These included superoxide dismutase (SOD), catalase (CAT), ascorbate peroxidase (APX), and peroxidase (POD) (Fig. 5C-D), as well as other protective enzymes, such as glutathione S-transferases (GSTs) that are also important for the synthesis of metal binding peptides such as metallothionein (Fig. 5). Interestingly, several major GST genes were clearly down-regulated by 0.4 mM Al, but also significantly up-regulated by 2.5 mM Al, such as the tau-type GST genes, TEA014619.1, TEA015778.1, TEA025564.1, TEA025571.1 (Fig. 5). But other classes of GST genes were down-regulated by 2.5 mM Al stress, indicating strong toxicity of Al (Table S5, Fig. S18-S20). Several genes for SOD and CAT isoforms as well as APX and POD were up-regulated in roots treated with 2.5 mM Al as compared with the control, suggesting that these genes were responsive to Al-induced oxidative stresses. All these data support the conclusion that in Al accumulating tea plants, heavy metal imbalance occurred and caused massive ROS generation and oxidative stress.

### Aquaporin transporter genes in tea roots induced by Al stress

**Fig. 6:**
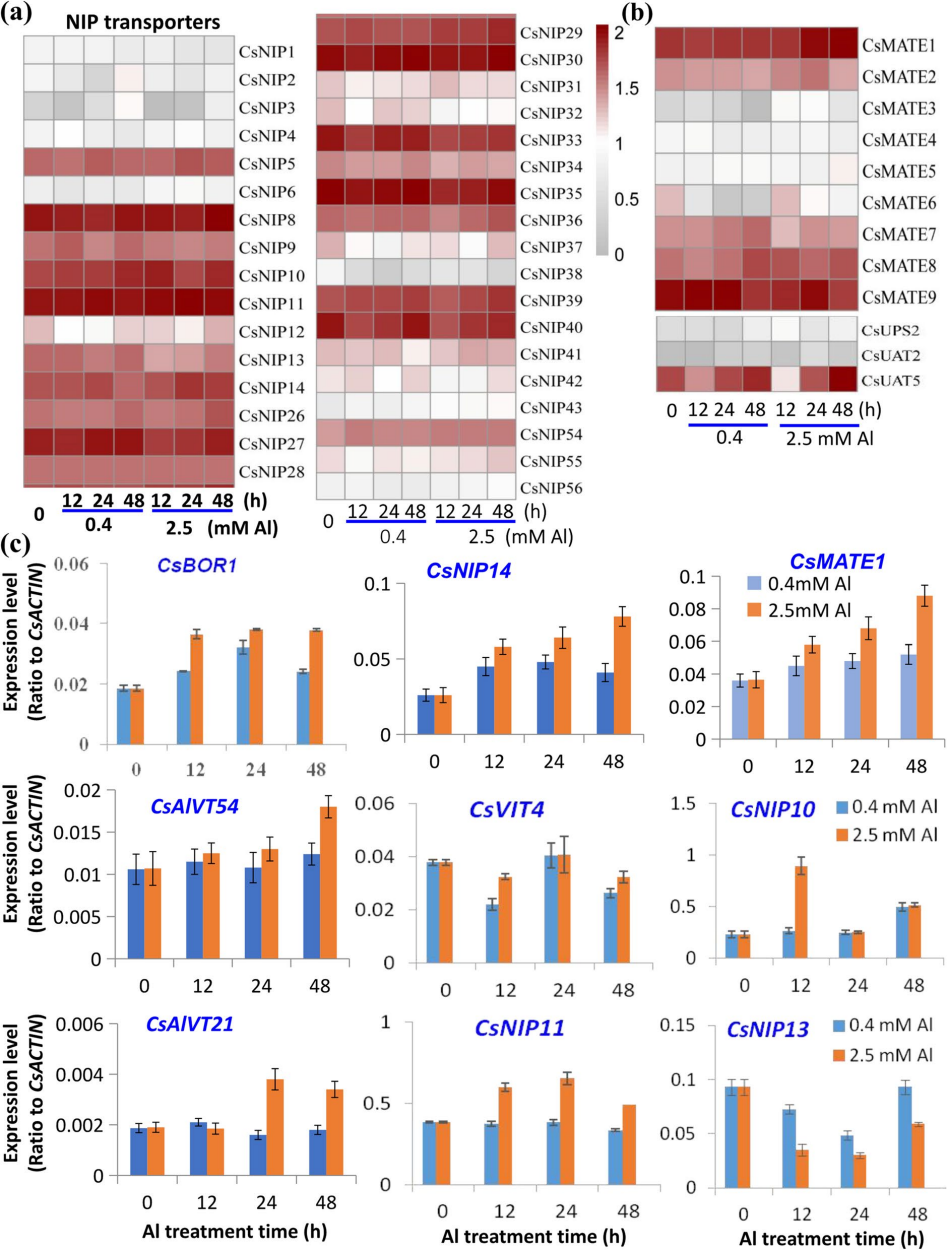
Effects of Al stress on NIP like aquaporin and other transporter genes. **a**, **b** Heatmap analysis of expression of genes encoding nodulin 26-like intrinsic protein (*CsNIP*) (a) and multidrug and toxin extrusion protein (*CsMATE*) and urate transporter (*CsUPS/UAT*) (**b**) in tea seedlings in response to Al. **c** qRT-PCR verification of gene expression for *CsNIP* genes in tea seedling roots in response to Al stress. The heatmaps were made with transcriptome data normalized as Log_10_(1 + log (1 + FPKM)). MeV software was used to depict transcript levels. qRT-PCR data are presented as means ± SD from three independent experiments and statistical significance is based on Student’s t-test (*P < 0.05; ** P < 0.001) compared with controls

The aquaporin nodulin 26-like intrinsic protein (NIP) type transporter can allow influx of water and other substrates such as metals into plant cells. For example, one route for Al entry across the plant plasma membrane in *A. thaliana* has been proposed to be via AtNIP1;2, which can be specifically induced by Al stress and can transport an Al-malate complex, but not Al^3+^ ions, into the symplasm, followed by further translocation from the root to the shoot, after subsequent Al xylem loading [44]. The formation of the Al-malate complex requires a functional root malate exudation system mediated by the Al-activated malate transporter ALMT1 [16, 44]. Involvement of NIP-like aquaporin transporters in Al tolerance have also been reported in other plants [44]. NIP1-like aquaporin transporter genes including TEA012255 (*CsNIP8*), TEA027942 (*CsNIP10*), TEA019952 (*CsNIP11*), and TEA024296 (*CsNIP14*) were up-regulated in response to Al as compared with the control (Fig. 6A, Table S6, Fig. S21). Some NIPs can also facilitate the transfer of silicon, which can benefit plant growth and help alleviate different abiotic stresses including metal toxicities [45–47]. The homologous NIP genes in tea plants are TEA004662 (*CsNIP2*) and TEA013255 (*CsNIP3*) that were all slightly down-regulated initially (12 h) after Al treatment, but then up-regulated at 48 h, particularly for *CsNIP3* with nearly a 6-fold increase. Finally, there were a large number of putative NIP genes including TEA008904 (*CsNIP32*), TEA009357 (*CsNIP33*), TEA015178 (*CsNIP35*), TEA016839 (*CsNIP37*), TEA019674 (*CsNIP38*) and TEA009696 (*CsNIP40*) that were decreased in transcript abundance following both 0.4 mM and 2.5 mM Al treatment compared to the no Al control (Fig. 6A, Table S6, Fig. S21). Boron (B) is an essential plant micronutrient that can be transported via boric acid channels AtNIP5;1, AtNIP6;1 and AtNIP7;1, and by a boric acid/borate exporter AtBOR1 in *A. thaliana* [46, 48, 49]. B decreases root Al uptake and translocation to the shoot in pea plants, and thus alleviates Al toxicity [50]. We observed a slight increased expression of B transporter genes, TEA013947 (*CsBOR1/CsNIP54*), TEA001638 (*CsBOR2/CsNIP55*), and TEA000759 (*CsBOR3/CsNIP56*) in 2.5 mM Al treated roots. Two tea plant homolog genes for AtNIP5;1 and AtNIP6;1 are TEA019654 (*CsNIP5*) and TEA030821 (*CsNIP4*), which were transiently up-regulated by 0.4 mM and 2.5 mM Al stress (Fig. 6B, Table S6, Fig. S21). Several highly up-regulated *CsNIP* genes by Al treatment were selected and verified with qRT-PCR (Fig. 6C).

Multidrug and toxin extrusion (MATE) transporters can mediate citrate secretion in root and vascular cells for metal ion transport and detoxification of Al^3+^ extracellularly [14–16]. MATE transporters include the *A. thaliana* ferric reductase defective 3 (FRD3), which plays an important role in Fe homeostasis by transporting citrate into vascular tissues and facilitating Fe or Al transport through the xylem [14, 15]. We also observed that FRD3-like MATE transporter genes were significantly up-regulated by Al-treatment, including TEA027978 (*CsMATE1*) and TEA013673 (*CsMATE2*), which were induced by 2.5 mM Al but not by 0.4 mM Al, while TEA008152 (*CsMATE5*) and TEA027978 (*CsMATE8*) were increased in both 0.4 mM and 2.5 mM Al stress (Fig. 6C). *CsMATE5* is highly expressed in tea old leaves, but also was induced by 2.5 mM Al stress, indicating that it may be involved in Al translocation to the old leaves (Fig. 6C, Fig. S24). TEA017874 (*CsMATE6*) was the only gene markedly down-regulated by Al exposure (Fig. 6C, Table S7–8, Fig. S23–24). A representative one, *CsMATE1*, were confirmed and verified with qRT-PCR (Fig. 6C).

### ABC and ALMT transporters were involved in transport of Al and other metal cations

**Fig. 7:**
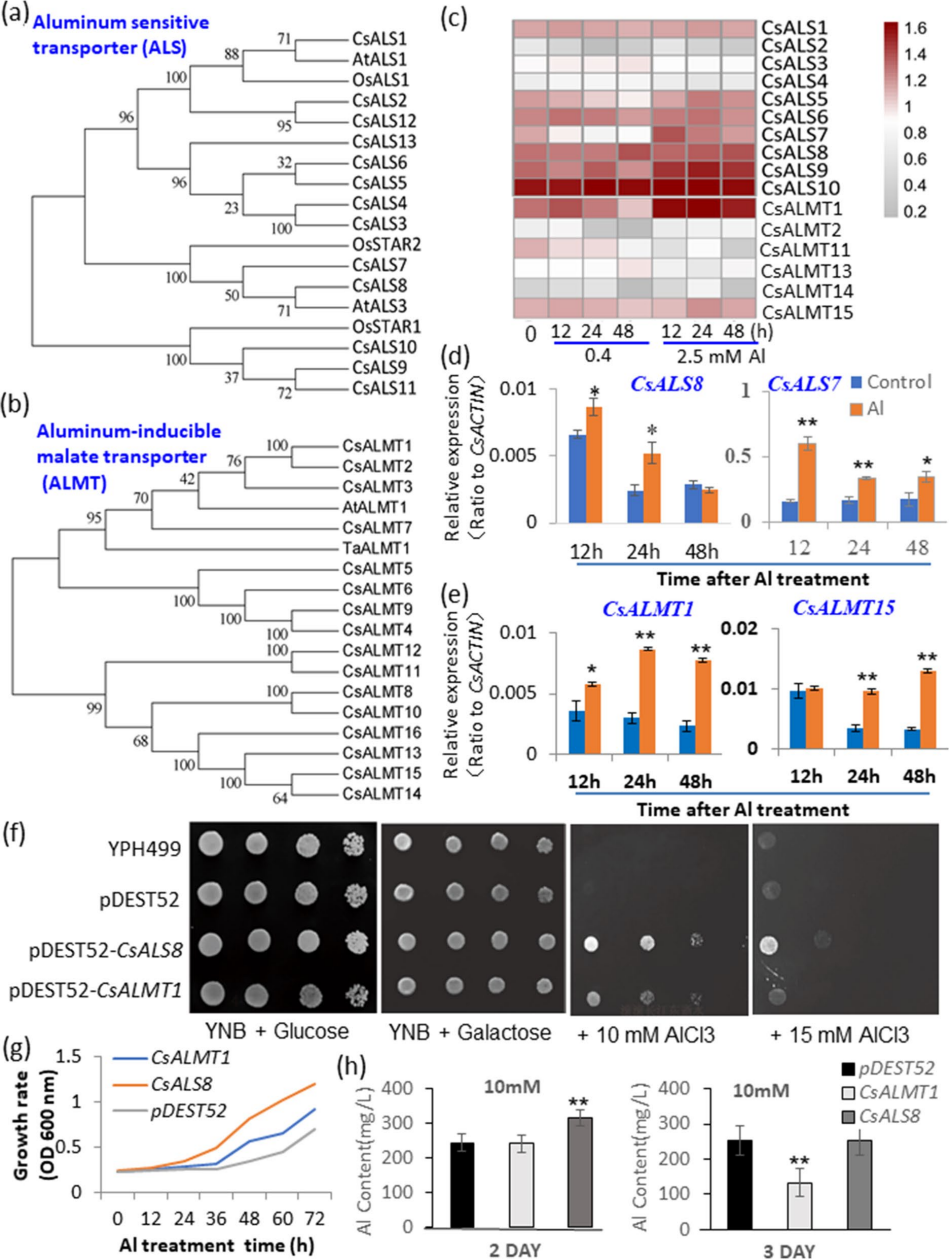
Functional identification of *CsALS8* and *CsALMT1* from tea plants. **a**, **b** Phylogenetic analysis of aluminum sensitive transporter (*CsALS*) (a) and aluminum-inducible malate transporter (*CsALMT*) (**b**) gene families in tea genome. **c** Heatmap analysis of expression of *CsALS7* and *CsALS8* homolog genes in tea seedling roots in response to Al stress. **d** qRT-PCR verification of *CsALMT1* and *CsALMT15* gene expression in tea roots in response to Al stress. **e** qRT-PCR verification of *CsALS8* and *CsALMT1* gene expression in tea roots in response to Al stress. **f** Growth test on the functions of CsALS8 and CsALMT1 in resistance to Al stress in yeast cells. **g** Growth curve of yeast strain YPH499 expressing empty vector pYESDEST52, *CsALS8*, or *CsALMT1* in response to Al stress. **h** Al content in the medium of yeast strain YPH499 expressing empty vector pYESDEST52, *CsALS8*, or *CsALMT1* in response to Al stress for 2 and 3 days. The heatmap was made with transcriptome data normalized as Log_10_(1 + log (1 + FPKM)). MeV software was used to depict transcript levels. qRT-PCR and Al content data are presented as means ± SD from three independent experiments and statistical significance is based on Student’s t-test (*P < 0.05; ** P < 0.001) compared with controls

ATP-binding cassette (ABC) family transporters represent one of the largest protein families in living organisms of most life domains [18]. Several ABC-transporters have been reported to directly be involved in transport Al/Al-organic acid (Al-AO) complexes, including into the vacuole and confer plant Al tolerance, such as *A. thaliana* AtALS1 and AtALS3, rice OsSTAR1 and OsSTAR2 [51–54]. OsSTAR1 homologous genes, including TEA027830 (*CsALS9*) and TEA023178 (*CsALS10*) were up-regulated in response to 2.5 mM Al treatment by up to 1.4-fold (for *CsALS10*) and up to 3-fold (for *CsALS9*) (Fig. 7, Table S7–8, Fig. S22). The AtALS3 homologous gene TEA002530 (*CsALS7*) was up-regulated in 2.5 mM Al treatment at 12 h by 2.8-fold, but down-regulated in response to 0.4 mM, while another AtALS3 homolog TEA025228 (*CsALS8*) was also up-regulated by 1.6-fold in 2.5 mM Al treatment at 48 h. The AtALS1 homologous gene TEA031570 (*CsALS1*) showed moderate up-regulation in response to Al (Fig. 7, Table S9, Fig. S22). The previously mentioned rice NRAMP transporter, NRAMP ALUMINUM TRANSPORTER 1 (OsNRAT1) can directly take up Al^3+^ from the extracellular space, and work together with the tonoplast ABC transporter ALS1 to detoxify Al^3+^ by sequestration into the vacuole [42]. Its homologous genes in tea plants, TEA017264 (*CsNRAMP1*) and TEA002435 (*CsNRAMP3*), were up-regulated by more than 2–3-fold by 2.5 mM Al stress (Fig. 4A).

Aluminum-induced malate transporters (ALMTs) are involved in organic acid release into the apoplast, particularly for malate to provide Al tolerance by chelation of excess Al^3+^ [6, 7, 15, 16]. The tea genome contains 16 putative ALMT genes, including homologs of HvALMT, TaALMT and AtALMT1; TEA013305 (*CsALMT1*) and TEA013312 (*CsALMT2*), which were both highly expressed in the roots and up-regulated in response to 2.5 mM Al (Fig. 7, Table S7–8, Fig. S23–24).

Next, *CsALMT1* and *CsALS8* were heterologously expressed in yeast cells to examine their effects on the resistance of yeast cells to 10 mM and 15 mM AlCl_3_ in the growth media at pH 4.5 (Fig. 7F). The growth of the wild type YPH449 yeast strain was inhibited by 10 mM Al when grown on both solid growth medium and liquid medium, while the growth of YPH449 cells transformed with the empty vector pDEST52 was similarly inhibited by 10 mM Al. However, the *CsALS8*-expressing yeast cells grew much faster than the control yeast cells expressing empty vector pDEST52, and was able to mediate growth on both 10 mM and 15 mM Al. Also, the *CsALMT1*-expressing yeast cells grew less well than *CsALS8*-expressing yeast cells, but faster than the control cells, indicating increased resistance to Al stress in solid growth medium and liquid medium (Fig. 7F). We also analyzed Al contents in the growth media of yeast cells expressing *CsALS8, CsALMT1*, and pDEST52. Al content remaining in the growth media after 2 days of cultivation was higher for the *CsALS8*-expressing yeast cultures than others, and after 3 days of cultivation, the Al content in the media was lowest for the *CsALMT1*-expressing yeast cultures (Fig. 7H). This was consistent with *CsALS8* expression in yeast cells conferring resistance to Al stress, potentially by restricting Al entry into the cells, such as through Al efflux. Furthermore, *CsALMT1* expression also conferred more resistance to Al than the pDEST52 control cells, potentially by enhancing Al-organic acid conjugate formation in the apoplastic space and reducing the availability of free Al^3+^ ions, which are otherwise toxic to yeast cells.

## Discussion

It has long been known that tea plants can accumulate and tolerate higher concentrations of Al compared to many other plant species [12, 17, 55], and that this characteristic is determined in part by large scale transcriptional changes in response to Al exposure, which include genes involved in Al transport and sequestration, biosynthesis and secretion of organic acids, and modification to the cell wall [12, 13]. It is also well known that metal responses are interactive and typically affect other metals, such that changes in the availability and plant accumulation of one metal, such as Al, typically alters the behavior of other metals and mineral nutrients. For example, in maize plants, Al exposure reduced the accumulation of Ca and Mg, while Al supply to tea leaves alters the uptake and transport of Fe and B [55]. While other studies have examined broad-scale biochemical and transcriptional responses to Al exposure in tea plants, this study aimed to specifically examine the ionomic and transcriptional responses in relation to metal transport and mineral nutrient homeostasis mechanisms in tea roots exposed to Al and found that there were indeed widespread changes in many processes related to many different metals and mineral nutrients.

As expected, increased Al exposure led to increased Al content in roots, potentially facilitated by plasma membrane channels, such as the aquaporins including NIP1;2 [44]. NIP1;2 like genes from tea, as well as other NIP isoforms, were transcriptionally increased in response to Al. AtNIP1;2 was characterized to transport Al-malate complex from cell wall into the symplasm, through which Al-malate can be further translocated from the root to the shoot, after subsequent Al xylem loading [15]. Most plants share common mechanisms to tolerate Al exposure and the increased Al content in cells. These include the exudation of organic acids such as malate and citrate from the roots to chelate and detoxify excess Al^3+^ in the apoplastic space and the sequestration of cellular Al into the vacuole [15, 16, 56]. While intracellular transport of citrate or malate may provide external or internal detoxification of Al in roots and leaves, ALS1 on the tonoplast, together with other transporters such as Nrat1, remove Al^3+^ from the cell wall and sequester it in the root-cell vacuole [15, 54]. Key organic acid transporters involved in Al tolerance include the putative ALMT type malate transporter [16], while other organic acid transporters involved in Al tolerance include the MATE type transporters [17]. We showed that a putative malate exporter *CsALMT1* in tea plant roots could confer a moderate tolerance to Al when over-expressed in yeast cells (Fig. 7). Some half-size ABC transporters, such as ALS1 and ALS3 in Arabidopsis [51, 52], and their tea homologs, including *CsALS5, 6, 7* and *8*, were all induced by Al in roots. Particularly, *CsALS8* was further demonstrated by using yeast heterologous expression to be able to provide strong Al tolerance (Fig. 7). Members of this family include AtALS1 and OsALS1 found on the vacuolar membrane of root tip cells that may transport Al-organic acid complexes into the vacuole to provide detoxification [51–54]. Furthermore, AtALS3 or OsSTAR2 localized at the plasma membrane of root phloem cells may upload Al-organic acid complexes into the vascular tissues for translocation of Al to above-ground tissues, such as old leaves, that are insensitive to Al stress [51, 53]. The correlation analyses on the expression changes of nearly 1000 transporter genes with altered metal contents in tea plant roots under various Al conditions showed that many of these transporter genes and metal changed are tightly correlated, either negatively or positively (Table. S10). Also, expression changes of a AtBOR1-like *CsNIP5* was highly correlated to Al accumulation, indicating the possible involvement of this transporter in Al tolerance (Table. S10).

The Al-citrate complex is a widely detected organic acid complex in many plants [15, 16]. The MATE gene FRD3 has been demonstrated to be involved in the translocation of citrate in the xylem to control Fe movement, but when overexpressed, FRD3 can provide Al tolerance [6, 7, 15]. The expression of two orthologs of FRD3 were highly induced by the Al treatment. This supports the speculation that in the presence of Al, the amount of citrate release to the xylem is increased in order to enhance Al-citrate translocation, requiring the induction of genes such as MATE transporters involved in citrate release. Once inside the root cells, Al can induce the peroxidation of membrane lipids and the production of ROS, which can lead to further cellular damage. The increased expression of antioxidant genes including members of the GST, GRX, SOD, and CAT families, indicate additional roles in mitigating Al toxicity [8, 11].

Our data clearly showed that high Al stresses caused significant losses of macronutrients, such as Mg and Ca, which are essentially required for plant growth, photosynthesis, and cell wall structure, function, and stability. Because of important cell wall structural and physiological roles of these two elements in plants, maintenance of high levels of Mg and Ca levels in roots and shoots are essential for normal plant growth and development [24, 25]. Deficiencies of these two major nutrients cause various symptoms, which could connect to the major causes for reduced root cell elongation and root growth by high Al and low pH. Changes in expression of candidate Ca transporter genes may in part explain the reduced Ca content. For example, members of the GLR and MSL families of Ca^2+^ influx channels that play a role in cellular Ca accumulation showed reduced expression. Likewise, some putative organellar Ca^2+^ transporters such as ACA Ca^2+^ pump isoforms that are important in providing Ca content into intracellular stores were also reduced in response to Al. However, loss of these Ca transport pathways is unlikely to significantly decrease total root Ca content [57]. In fact, transcriptional modification of these Ca^2+^ transporters is more likely to play a key role in altering the generation of cellular Ca^2+^ signals, which may in turn mediate adaptive responses [20]. Al stress often causes an increase in cytosolic Ca^2+^ spiking, which plays a role in activating defense responses against Al toxicity [7, 21]. A correlation was observed between the rapid Al-induced increase in cytosolic Ca^2+^ and root growth inhibition in wheat, leading to the hypothesis that disruption of Ca^2+^ homeostasis may be the primary cause of Al^3+^ toxicity [21].

The Al-induced inhibition of root Mg content might also have been due to the repression of Mg transporter genes, such as members of the MGT family. The change in profile of tea MGT genes was variable as some were enhanced by Al exposure and others were reduced. Al-Mg interaction via this class of transporter has previously been observed. Overexpression of MGT1 enhanced Al tolerance [24, 25]. Mg^2+^ in the cytoplasm directly regulates H^+^-ATPase activity, which maintain the membrane potential and pH gradient across the plasma membrane [24, 25]. Al stress inhibits H^+^-ATPase activity, but Mg addition can restore activity, thus alleviating Al toxicity [62]. In addition, it is hypothesized that enhanced Mg nutrition under Al stress may prevent Al-induced perturbation of cytosolic Ca^2+^ signaling [24].

Enhanced Fe uptake was observed in the tea plant roots under Al stress, likely to result from altered expression of Fe transporter genes, although this observation contrasts with previous studies that showed Al stimulated reduction in Fe uptake and transport [55]. Most dicots, including tea plants, use a FRO isoform to reduce Fe^3+^ to Fe^2+^, which can then be transported into the root epidermis by the divalent metal transporter IRT1, both of which were increased. Other components that can enhance the accumulation and transfer of Fe such as NAS, FER and YSL were also enhanced [31, 33]. Fe is essential for plant growth, respiration, and synthesis of Fe-S clusters. However, the increased intracellular concentration of free Fe is also cytotoxic since Fe is highly reactive via the Fenton reaction to generate ROS [31, 33]. Thus, plants tightly control Fe homeostasis by multiple strategies, depending on the types of plants, to react to Fe deficiency as well as Fe overload. So the transcriptional increase of the ROS scavenging mechanisms that were observed, components to sequester Fe into intracellular stores such as the ortholog of the vacuolar transporter VIT1 were increased [32].

## Conclusions

Tea plants actively take up Al under acidic conditions, and transport Al from roots to above ground tissues, therefore it was important to understand the processes of Al accumulation and other metals in root tissues. Al stress significantly reduced root Mg, Ca, and Mn uptake, but increased Fe uptake. Al stress modified metal cation transporter gene profiles as compared with controls. These altered transporter gene expression patterns may explain why imbalanced metal ion accumulation occurred in the Al-stressed roots. Over-accumulation of Al, and other toxic metals, such as Cd in tea plants could cause public concerns over the safety of consuming tea products [1, 2]. The improved understanding gained from this study of the genetic responses to Al exposure, particularly in relation to metal transport and homeostatic processes, has highlighted potential gene targets for breeding or genetic engineering approaches to develop safer tea products.

## Materials and methods

### Plant materials and growth conditions

The collection of tea plant material in this study had complied with relevant institutional, national, and international guidelines and legislation. Seedlings of the tea plant (*Camellia sinensis* L.O. Kuntze) cultivar “Shuchazao” were used for all experiments. This cultivar was obtained from the Guohe tea garden (Anhui Agricultural University), Hefei, Anhui province, China. About 1.5-year old tea plant (Shuchazao) seedlings were used for Al treatment experiments in hydroponic solutions. Preliminary Al treatments were performed to ascertain the optimum Al concentration and the toxic exposure concentration for tea plants, by using a concentration range of 0, 0.4, 2.5, and 5 mM Al delivered to the plants using a hydroponics system. The Al concentration at 0.4 mM provided the best performance of root growth, but for analysis of Al responses, the no Al (0 mM Al) treatment was used as a control concentration. The hydroponics SK-solution (pH was set to 4.3 as the initial value) contained (NH_4_)_2_SO_4_ (1.07 mM), Ca (NO_3_)_2_·4H_2_O (0.357 mM), KH_2_PO_4_ (0.1 mM), K_2_SO_4_ (0.413 mM), CaCl_2_·2H_2_O (0.392 mM), MgSO_4_·7H_2_O (1.029 mM), C_10_H_12_FeN_2_NaO_8_ (6.27 μM), H_3_BO_3_ (9.25 μM), MnSO_4_·7H_2_O (18.2 μM), ZnSO_4_·7H_2_O (1.53 μM), CuSO_4_·5H_2_O (0.39 μM), Al_2_(SO_4_)_3_·18H_2_O (0.4 mM) and Na_2_MoO_4_·2H_2_O (0.52 μM), with modifications to the Al concentration as needed. The solution was aerated by using an air pump and refreshed with fresh medium once every 5 days. Tea plant seedlings were grown under a natural photosynthetic photon flux density of 1300 μmol m^− 2^ s^− 1^ with a diurnal cycle of 18 h day/6 h night, Temperature (24 °C /18 °C, day/night) and humidity (70–80%) were controlled in the cabinet.

### RNA-Seq, DEG analysis, and qRT-PCR validation

After tea seedlings were exposed to 0, 0.4, or 2.5 mM Al for up to 48 h in SK solution (about 50 seedlings for each concentration of Al), roots of at least 3 seedlings were harvested and then frozen in liquid nitrogen before total RNA was extracted using a General Plant RNA Extraction Kit (BioTeke, China). The extracted RNA was digested with DNase I (TAKARA) to remove contaminated genomic DNA. mRNAs were purified from the total RNA using Dynabeads Oligo (dT) (Life Technologies). The derived mRNAs were reverse transcribed into first-strand cDNAs with random hexamer and then the second-strand cDNAs were synthesized according to the manufacturer’s instructions. The double-stranded cDNAs were purified and ligated to adaptors for Illumina paired-end sequencing. The cDNA library was sequenced using the Illumina HiSeq2500 system by BGI (Shengzhen, China).

A range of 55.1–66.6 million clean reads was obtained for each repeat sample, and in total there were 767.4 million clean reads generated for all samples. Both a de novo assembly strategy (the Trinity method) with the regular parameters [58], and assembly against the tea plant reference genome [58] was used. All-predicted coding sequences were verified with protein databases including NCBI, Swiss-Prot, KEGG and COG. 45,737 transcript contigs were also assembled by BLAST against tea plant genome sequences. About 86% of the transcript contigs were mapped to the tea plant genome sequence of *C. sinensis var. sinensis* [58]. The differential expression genes (DEGs) were identified and modified by a rigorous algorithm. A false discovery rate (FDR) ≤ 0.001 and an absolute value of log2Ratio ≥ 1 were chosen as the thresholds to determine the significance of differentially expressed genes. For a given unigene, the corresponding FPKM value for transcripts were determined. Hierarchical clustering was carried out using Cluster 3. Differentially expressed genes were extracted for GO functional enrichment analysis and KEGG pathway enrichment analysis. The enrichment analysis was tested using a hypergeometric test at a significance cutoff of ~ 0.1% FDR. The assembled contigs had a length distribution ranging from 201 to 25,284 bp, with an average length of 1184 bp with a high read coverage for the contigs.

To validate the RNA-Seq data for the expression of key genes involved in organic acid and metal ion transport, qRT-PCR was performed with RNA samples prepared from Al treatment experiments. The single-stranded cDNAs used for real-time PCR analysis were synthesized from the RNAs using a Prime-Script™ 1st Strand cDNA Synthesis Kit (TaKaRa, Dalian, China) based on three biological replicates and three technical replications. In addition, detailed information of the validated transcripts, including unigene IDs and the primer pairs designed for qPCR analysis in this study, is presented in Supplemental Table S11. An IQ5 real-time PCR detection system (Bio-Rad) was utilized as previously described [15, 23]. *CsACTIN* was used as an internal reference gene, and relative expression was calculated relative to the optimal Al concentration of 0.4 mM Al using the 2ΔCt method. A total of 11 transcripts were originally validated by qPCR and then compared with transcriptome data, that we found the transcriptome data were highly reliable.

### Microscopic observations

After treatment of tea plant seedlings with 0, 0.4, 2.5 and 5 mM Al in SK medium for various times, roots from different plants were fixed with ethanol and stained with DAPI for 15 min for cell death examination. ROS generation in roots was observed by using 2,7-DCFDA as a reporter. Seedlings were treated with Al for different time and then were put into a fresh PBS buffer; 5 μg ml^− 1^ of 2,7-DCFDA were added to stain the ROS [8]. Roots were imaged under a fluorescence microscope (Nikon Eclipse 800, Japan) with excitation at 488 nm and emission at 540 nm to detect green fluorescence intensity. Roots were stained with 1 mg ml^− 1^ DAB solution (pH 4.0) for 8 h for examination of the presence of H_2_O_2_ in root cells [59]. Tissue- or cell-specific accumulation of Al in tea seedling roots were determined using Morin staining according to the method described previously [8]. Briefly, 10 μM morin in DMSO solution was added to the root tips on the glass plate for staining for 15 min. Excitation and emission wavelengths of 420 nm and 515 nm, respectively, were used for imaging the Al–morin complex in the roots [8].

### Transporter gene cloning and functional characterization

The open reading frames (ORFs) for *CsALS8* and *CsALMT1* genes were obtained by PCR amplification using gene-specific primer pairs based on RNA-Seq sequence information. Total RNA was extracted from roots, 5 μg of which were used to synthesize the first-strand cDNA using the first-strand synthesis system Superscript III (Invitrogen, USA). The cDNA library was used as a template for gene amplification and performed using short primers with the Ex-Taq DNA polymerase (TAKARA): 5 min at 94 °C, followed by 30 s at 94 °C, 30 s at 58, 1.30 min at 72 °C, and then 10 min at 72 °C, for 30 cycles. The PCR products were purified and cloned into the Gateway entry vector pDONR221 by using BP Clonase (Invitrogen, USA). The resulting pDONR221 constructs harboring target genes were confirmed by sequencing and recombined by using Gateway LR Clonase (Invitrogen, USA) into the destination vector pYEST-DEST52 for yeast strains expression. These final constructs containing *CsALS8* and *CsALMT1* genes were also confirmed by sequencing. The Genbank accession numbers for these genes are: *CsALMT1* (OK562691) and *CsALS8* (OK562692).

For the functional characterization of *CsALS8* and *CsALMT1*, the wild-type yeast strain YPH499 (MATa *ura3–52 lys2-801_amber ade2–101_ochre trp1-Δ63 his3-Δ200 leu2-Δ1*) was used for transformation with *pYESDEST52-CsALS8*, or *pYESDEST52-CsALMT1* constructs, with empty vector pYESDEST52 as a control, by using the PEG/lithium acetate method [60]. Transformants were selected on YNB lacking Uracil and functional expression and induction of *CsALS8* and *CsALMT1* were performed as described previously [60]. YPH499 cells expressing empty vector pYESDEST52, *CsALS8*, or *CsALMT1* were grown in media containing galactose or glucose, supplemented with different concentrations of Al, then were examined for their growth response to Al stress for 2 and 3 days. Growth rates were assayed in 24-well plates and Al contents in yeast cells were determined under different conditions.

### Determination of metal accumulation by ICP-MS

Accumulation of Al, Ca, Cd, Cu, Fe, Mg, Mn, and Zn in tea roots, and of Al in yeast cells or in yeast growth medium upon Al treatment were determined by using ICP-MS (inductively coupled argon plasma mass spectrometer) [60]. The roots or yeast cells were washed with 5 mM CaCl_2_ solution for 10 min before further rinsed in ddH_2_O. Al in the yeast growth medium was measured, according to the method described previously [8, 60].

## Phylogenetic analysis

Phylogenetic analysis was carried out by using the MEGA4.0 program (http://www.megasoftware.net/). The neighbor-joining method was used to construct the phylogenetic tree with 1000 bootstrap trials by MEGA4.0.

### Statistical analysis

All experimental data were from at least three independent experiments with duplicate sampling. A difference between two groups of data, control and treatment, was considered significant when *p* < 0.05 in the Student’s *t*-test.

## Supplementary Information

**Additional file 1.**

**Additional file 2.**

## Data Availability

The datasets generated and/or analyzed during the current study are available in the supporting information. The original RNA-seq data have been deposited in the NCBI Sequence Read Archive, accession number: PRJNA- (https://www.ncbi.nlm.nih.gov/sra/PRJNA-).
