# Supplementary material for: Tea plant roots respond to aluminum-induced mineral nutrient imbalances by transcriptional regulation of multiple cation and anion transporters
Source: BMC Plant Biol. 2022 Apr 19;22:203. doi: 10.1186/s12870-022-03570-4 (PMC9017051; doi:10.1186/s12870-022-03570-4)
Supplement: Supplementary file 2 — Additional file 2. [file 12870_2022_3570_MOESM2_ESM.pdf]

Endoplasmic reticulum (ER)-Calcium-ATPase (*CsECA*) and autoinhibited calcium ATPase (*CsACA*)

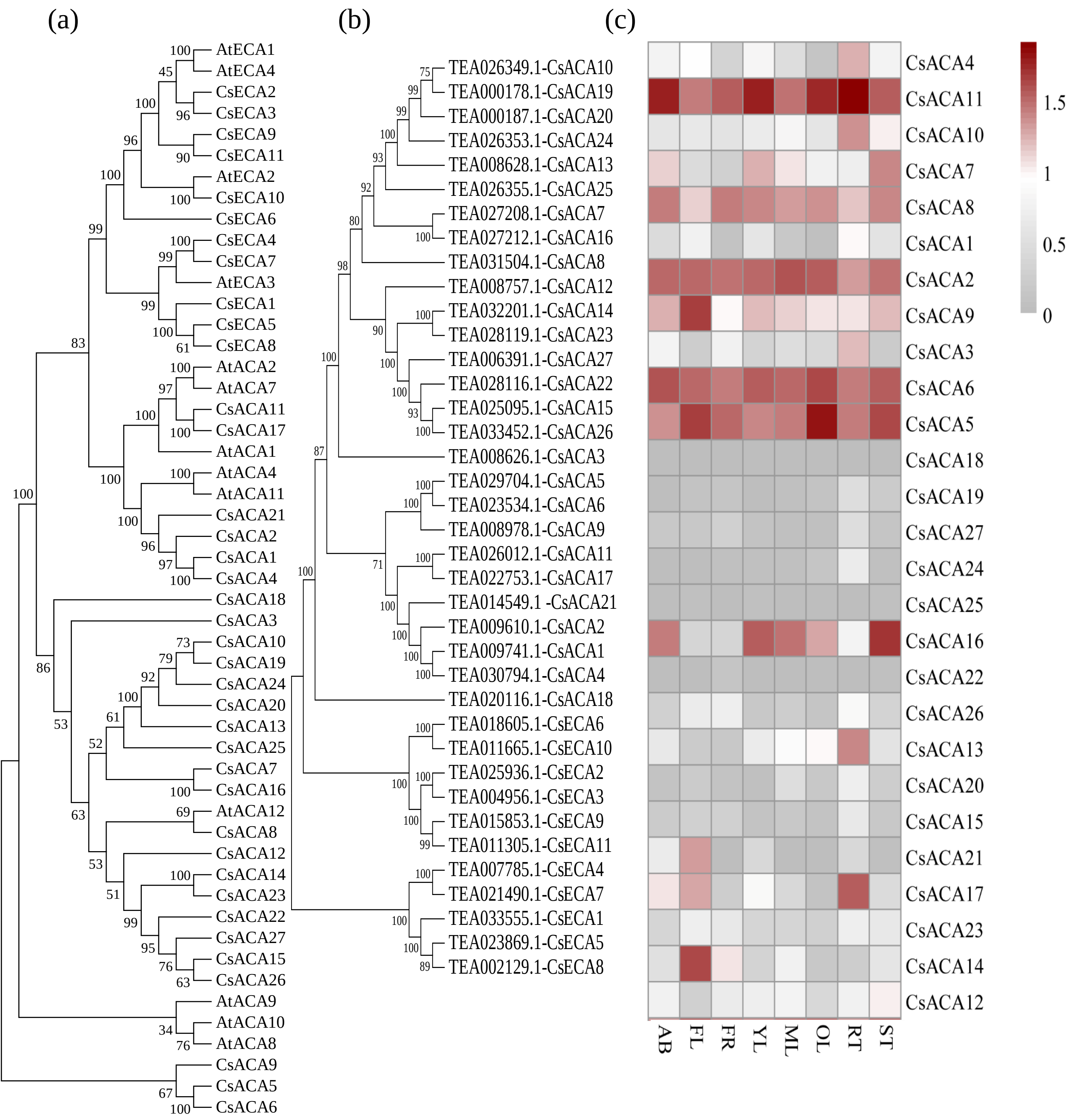

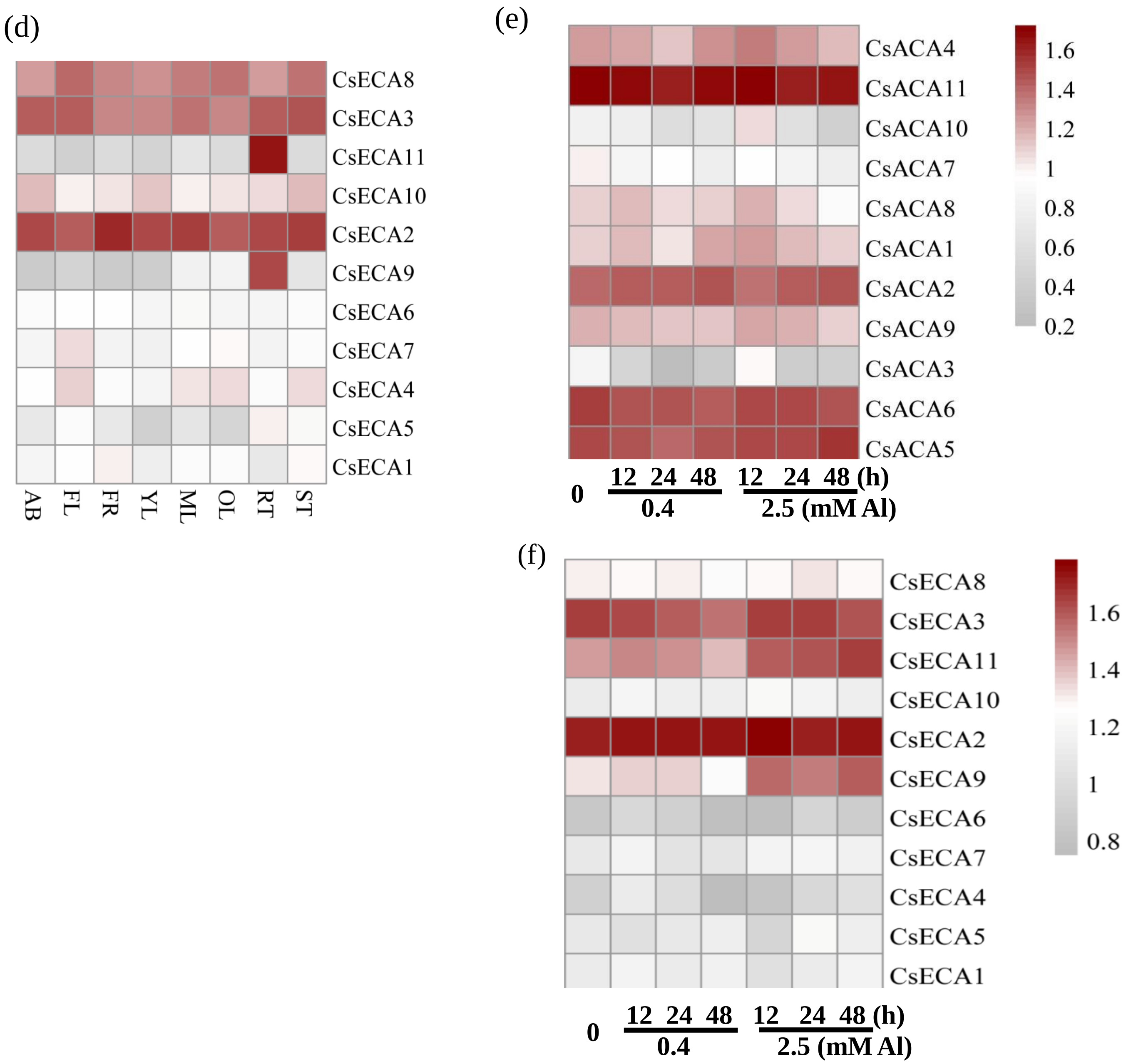

Fig S1 Identification and expression patterns of endoplasmic reticulum (ER)-Calcium-ATPase (*CsECA*) and autoinhibited calcium ATPase (*CsACA*) gene family in *C. sinensis.*

(a) Phylogenetic analysis of endoplasmic reticulum (ER)-Calcium-ATPase (*CsECA*) and autoinhibited calcium ATPase (*CsACA*) genes homology to functional characterized ones in Arabidopsis

(b) Annotation of endoplasmic reticulum (ER)-Calcium-ATPase (*CsECA*) and autoinhibited calcium ATPase (*CsACA*) genes in *C. sinensis.*

(c) and (d) Expression patterns of autoinhibited calcium ATPase (*CsACA*) genes and endoplasmic reticulum (ER)-Calcium-ATPase (*CsECA*) genes in various tissues of tea plants

(e) and (f) Expression patterns of autoinhibited calcium ATPase (*CsACA*) genes and endoplasmic reticulum (ER)-Calcium-ATPase (*CsECA*) genes in tea plant roots in response to Al stress for various times

Ca(2+)/H(+) antiporter (*CsCAX*) and Cation/calcium exchanger (*CsCCX*)

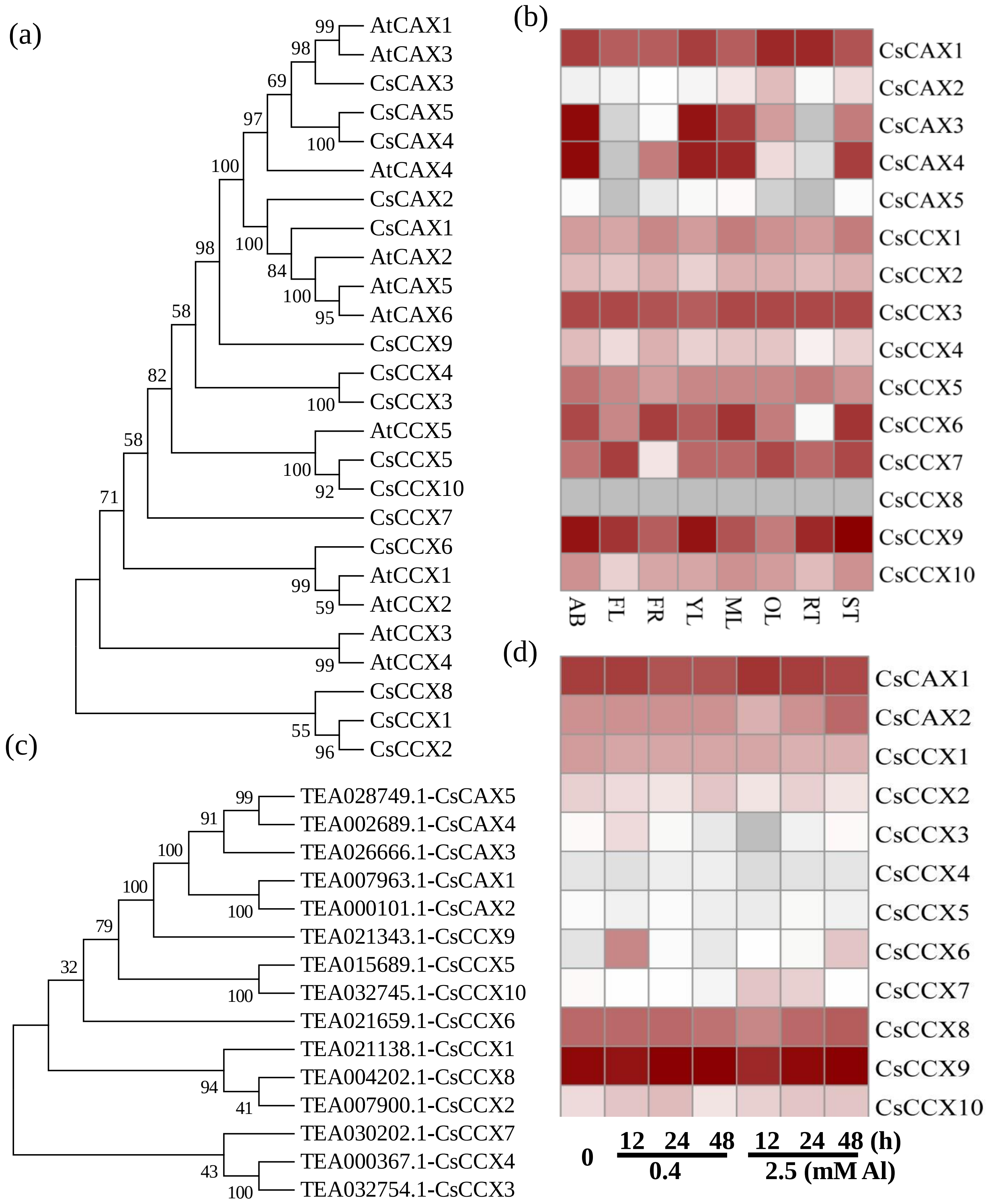

Fig S2 Identification and expression patterns of Ca(2+)/H(+) antiporter (*CsCAX*) and Cation/calcium exchanger (*CsCCX*) gene family in *C. sinensis*.
(a) Phylogenetic analysis of Cation/calcium exchanger (*CsCCX*) gene homology to functional characterized ones in Arabidopsis
(b) Annotation of Cation/calcium exchanger (*CsCCX*) gene in *C. sinensis*.
(c) Expression patterns of Ca(2+)/H(+) antiporter (*CsCAX*) gene in various tissues of tea plants
(d) Expression patterns of Ca(2+)/H(+) antiporter (*CsCAX*) gene in tea plant roots in response to Al stress for various times

Annexin (Ann)

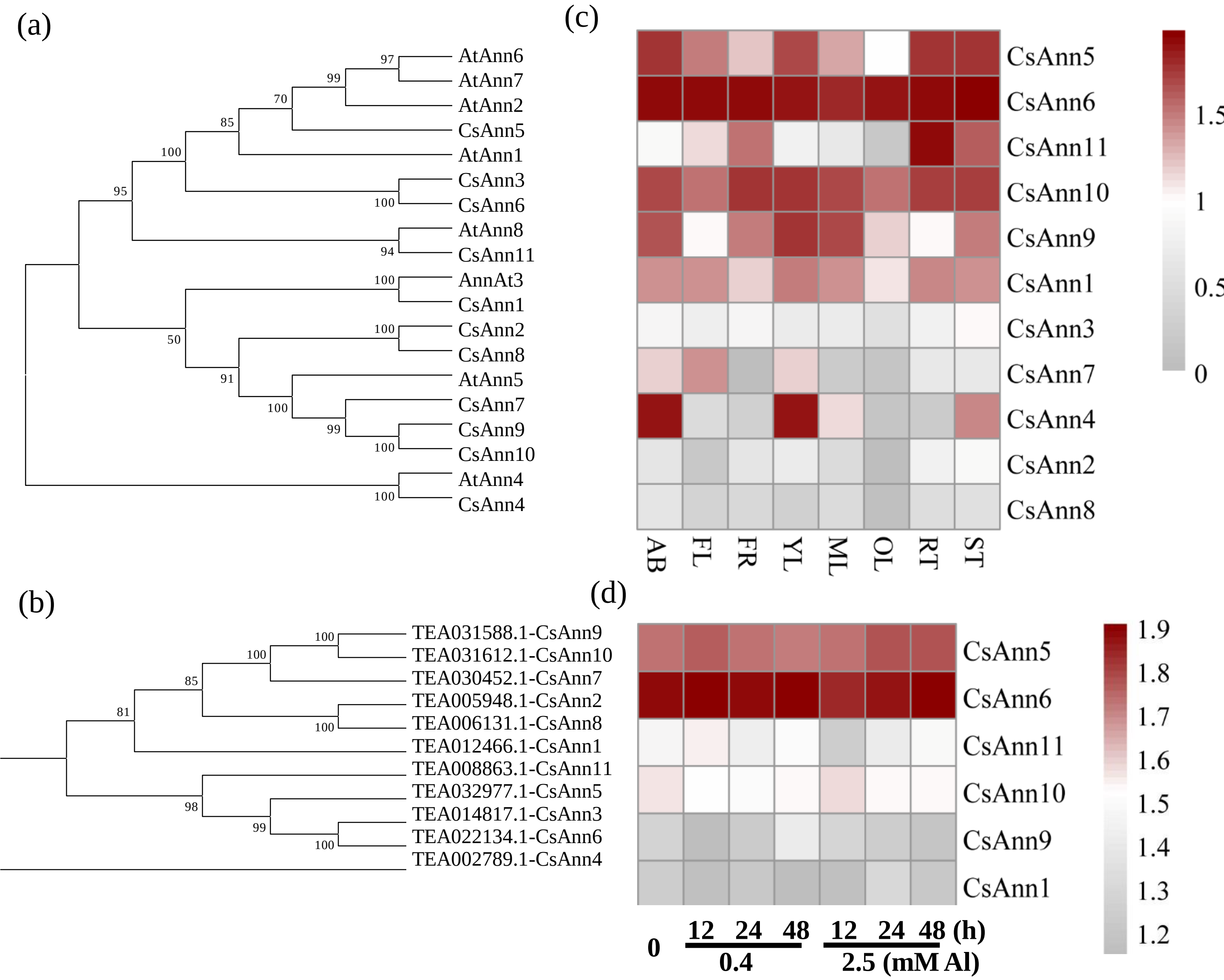

Fig S3. Identification and expression patterns of annexins (*CsAnn*) gene family in *C. sinensis*.
(a) Phylogenetic analysis of annexin (*CsAnn*) genes homology to functional characterized ones in Arabidopsis
(b) Annotation of annexin (*CsAnn*) genes in *C. sinensis*.
(c) Expression patterns of annexin (*CsAnn*) genes in various tissues of tea plants
(d) Expression patterns of annexin (*CsAnn*) genes in tea plant roots in response to Al stress for various times

mechanosensitive ion channel protein (*CsMSL*)

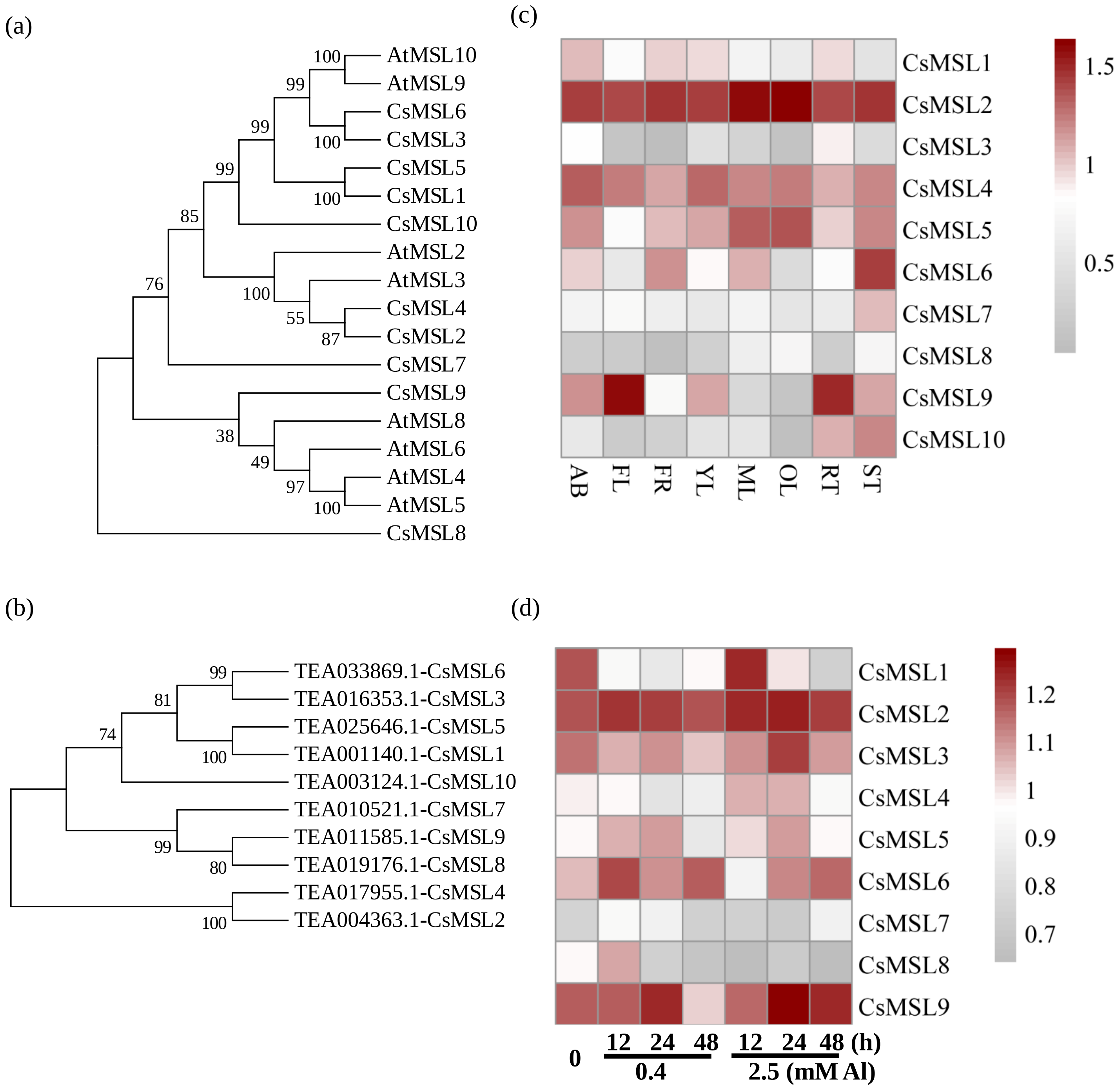

Fig S4 Identification and expression patterns of mechanosensitive ion channel protein (*CsMSL*) gene family in *C. sinensis.*

(a) Phylogenetic analysis of mechanosensitive ion channel protein (*CsMSL*) genes homology to functional characterized ones in Arabidopsis

(b) Annotation of mechanosensitive ion channel protein (*CsMSL*) genes in *C. sinensis.*

(c) Expression patterns of mechanosensitive ion channel protein (*CsMSL*) genes in various tissues of tea plants

(d) Expression patterns of mechanosensitive ion channel protein (*CsMSL*) genes in tea plant roots in response to Al stress for various times

glutamate receptor (*CsGLR*)

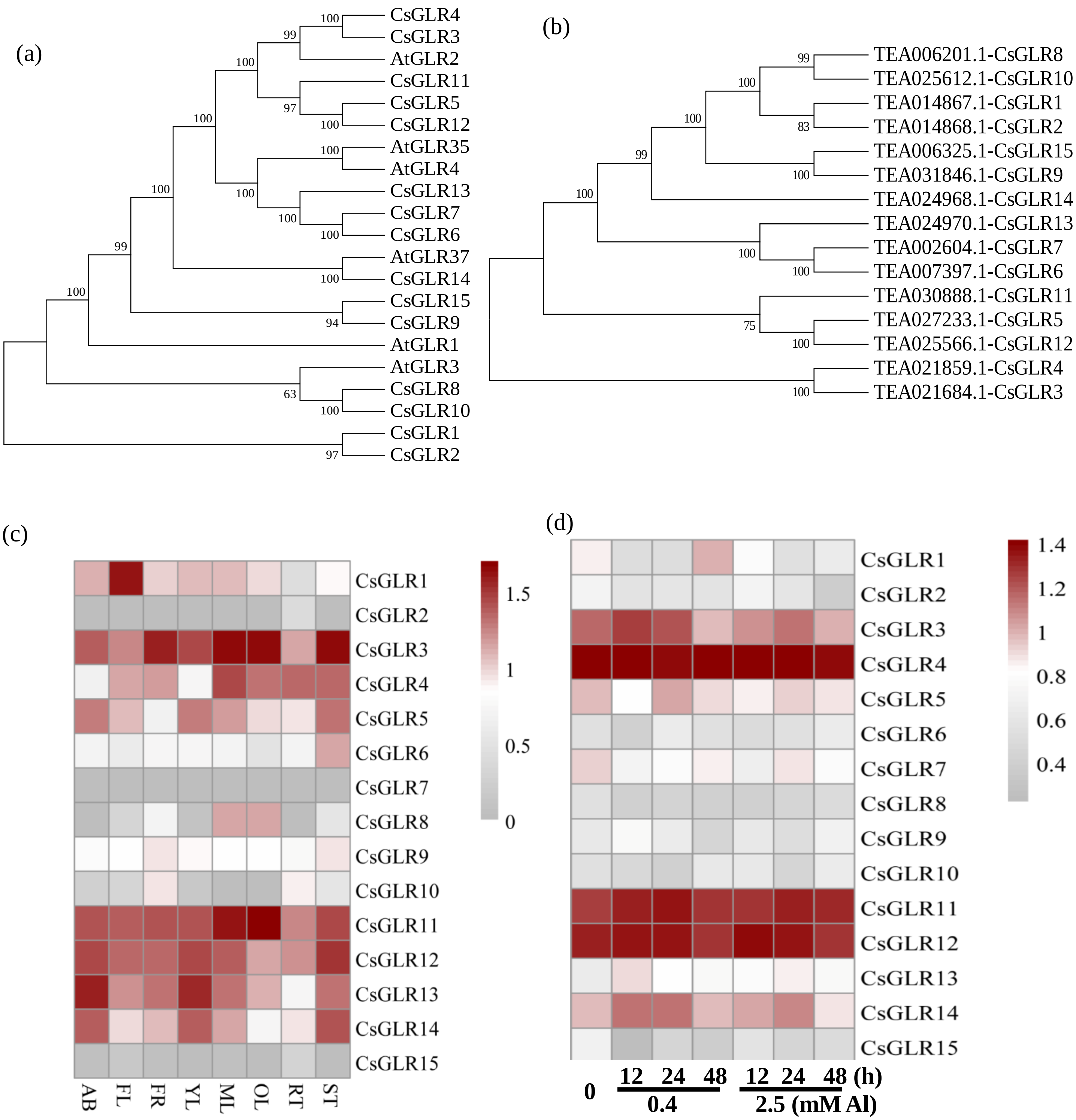

Fig S5 Identification and expression patterns of glutamate receptor (*CsGLR*) gene family in *C. sinensis*.
(a) Phylogenetic analysis of glutamate receptor (*CsGLR*) genes homology to functional characterized ones in Arabidopsis
(b) Annotation of glutamate receptor (*CsGLR*) genes in *C. sinensis*.
(c) Expression patterns of glutamate receptor (*CsGLR*) genes in various tissues of tea plants
(d) Expression patterns of glutamate receptor (*CsGLR*) genes in tea plant roots in response to Al stress for various times

Cyclic nucleotide-gated ion channel (*CsCNGC*)

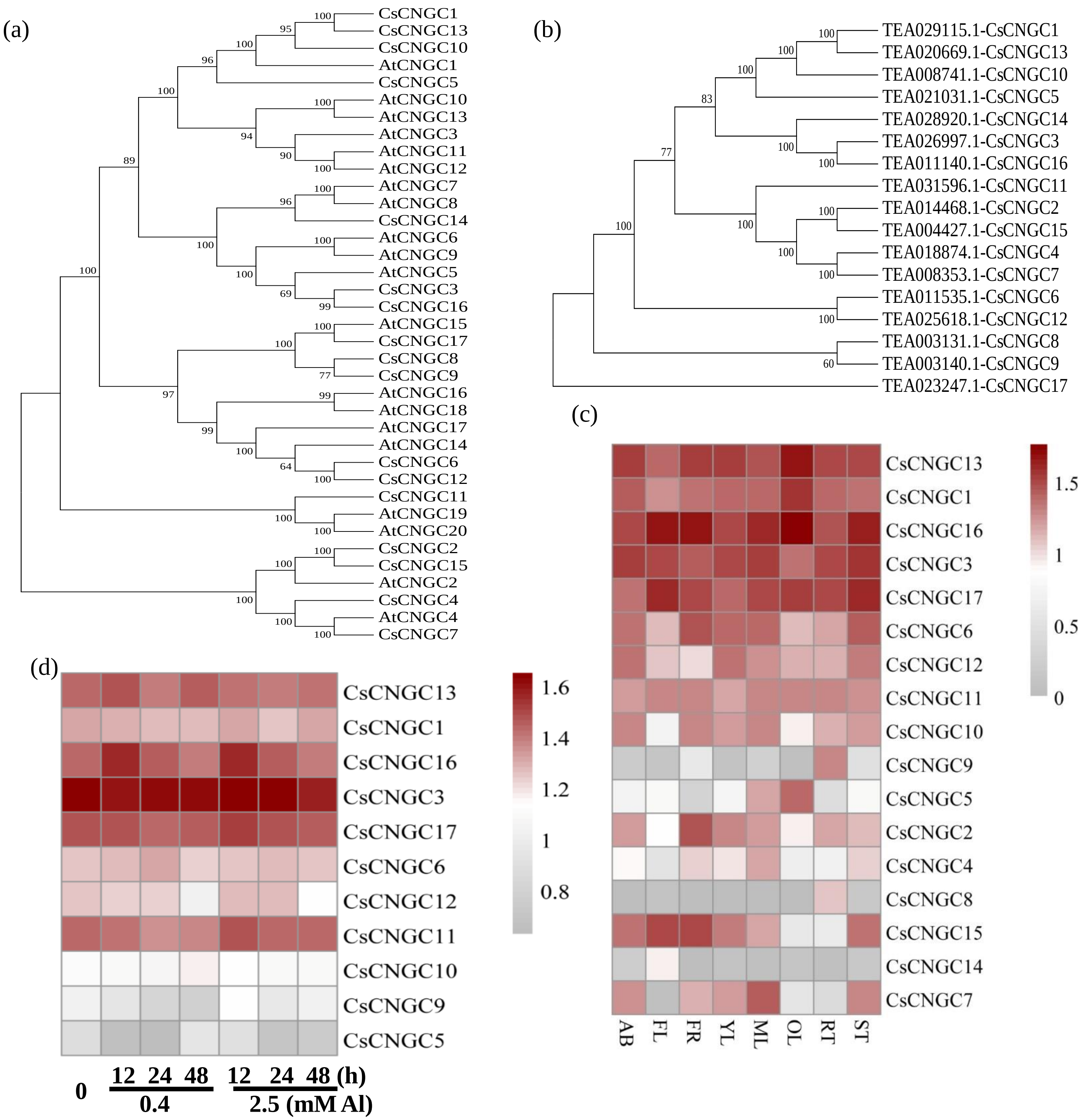

Fig S6 Identification and expression patterns of Cyclic nucleotide-gated ion channel (*CsCNGC*) gene family in *C. sinensis.*

(a) Phylogenetic analysis of Cyclic nucleotide-gated ion channel (*CsCNGC*) genes homology to functional characterized ones in Arabidopsis

(b) Annotation of Cyclic nucleotide-gated ion channel (*CsCNGC*) genes in *C. sinensis.*

(c) Expression patterns of Cyclic nucleotide-gated ion channel (*CsCNGC*) genes in various tissues of tea plants

(d) Expression patterns of Cyclic nucleotide-gated ion channel (*CsCNGC*) genes in tea plant roots in response to Al stress for various times

mitochondrial Calcium uniporter protein (*CsMCU*)

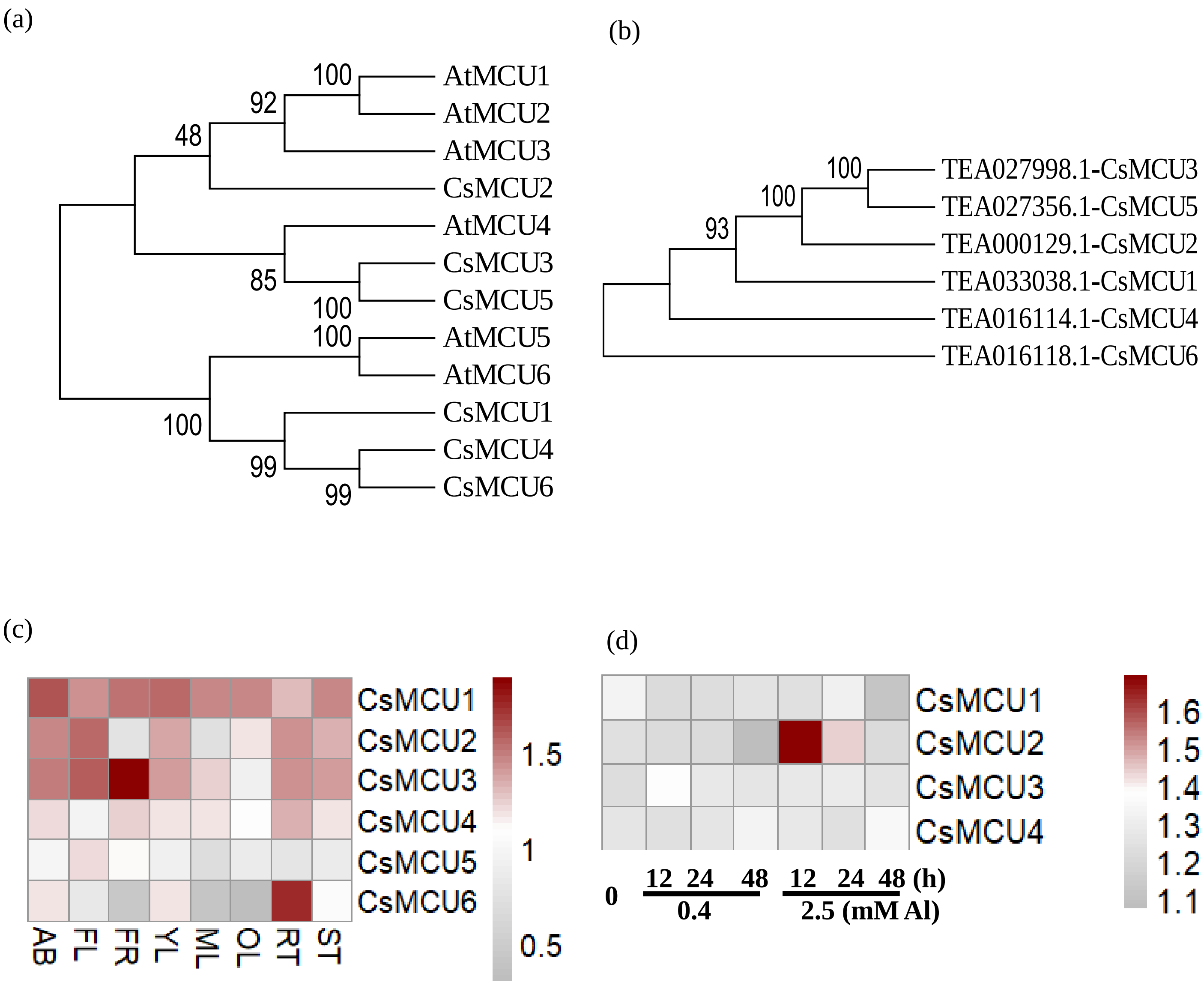

Fig S7 Identification and expression patterns of mitochondrial Calcium uniporter protein (*CsMCU*) gene family in *C. sinensis*.

(a) Phylogenetic analysis of mitochondrial Calcium uniporter protein (*CsMCU*) genes homology to functional characterized ones in Arabidopsis

(b) Annotation of mitochondrial Calcium uniporter protein (*CsMCU*) genes in *C. sinensis.*

(c) Expression patterns of mitochondrial Calcium uniporter protein (*CsMCU*) genes in various tissues of tea plants

(d) Expression patterns of mitochondrial Calcium uniporter protein (*CsMCU*) genes in tea plant roots in response to Al stress for various times

Mg **Magnesium Transporter (*CsMGT*) and Mg2+/H+ exchanger (*CsMHX*)**

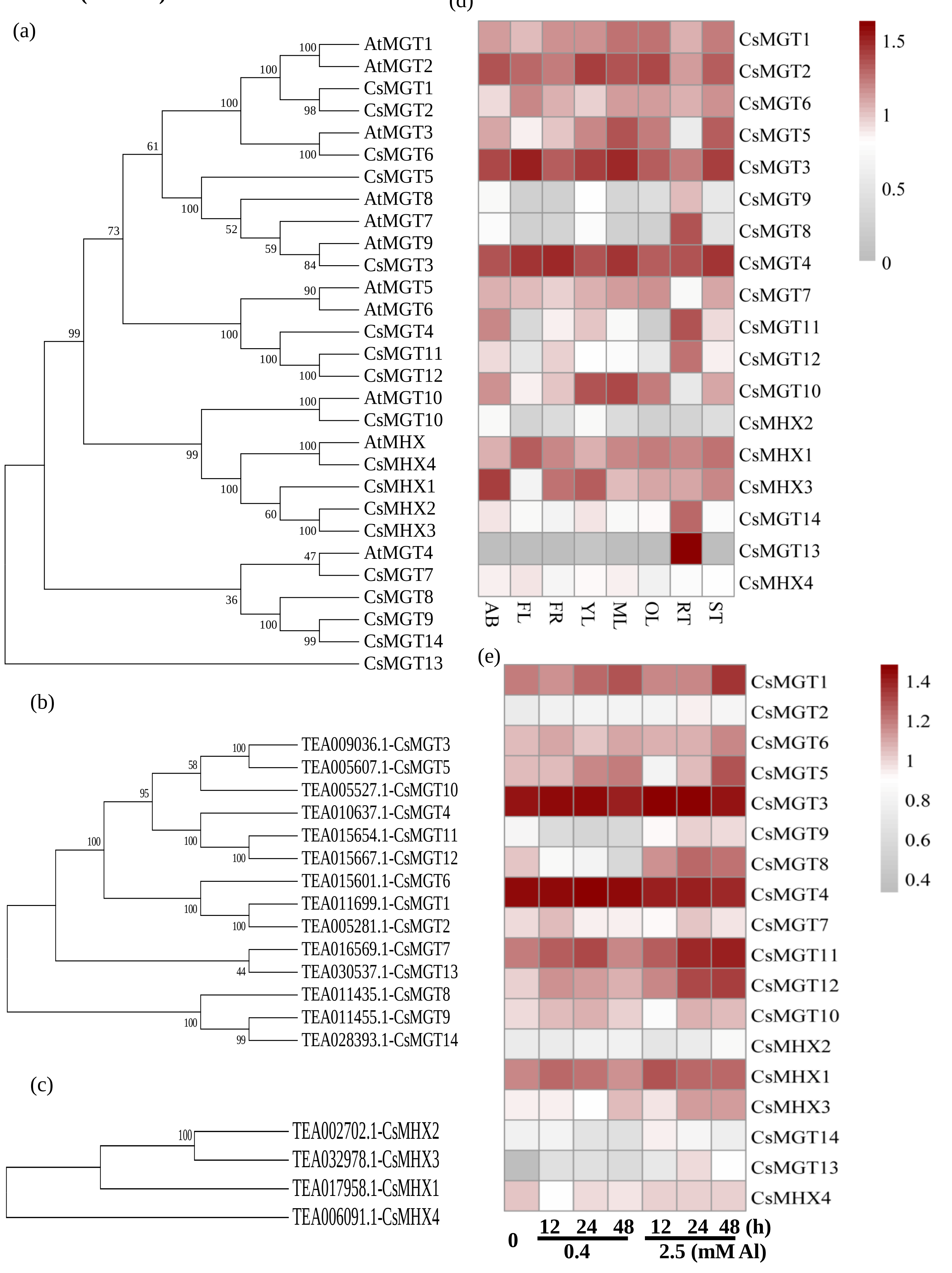

Fig S8 Identification and expression patterns of Magnesium Transporter (*CsMGT*) and Mg(2+)/H(+) exchanger (*CsMHX*) gene family in *C. sinensis.*
(a) Phylogenetic analysis of Magnesium Transporter (*CsMGT*)
and Mg(2+)/H(+) exchanger (*CsMHX*)gene homology to functional characterized ones in Arabidopsis
(b)(c) Annotation of Magnesium Transporter (*CsMGT*) and Mg(2+)/H(+) exchanger (*CsMHX*) genes in *C. sinensis.*
(d) Expression patterns of Magnesium Transporter (*CsMGT*) and Mg(2+)/H(+) exchanger (*CsMHX*) genes in various tissues of tea plants
(e) Expression patterns of Magnesium Transporter (*CsMGT*) and Mg(2+)/H(+) exchanger (*CsMHX*) gene in tea plant roots in response to Al stress for various times

Fe ferric reduction oxidase (*CsFRO*)

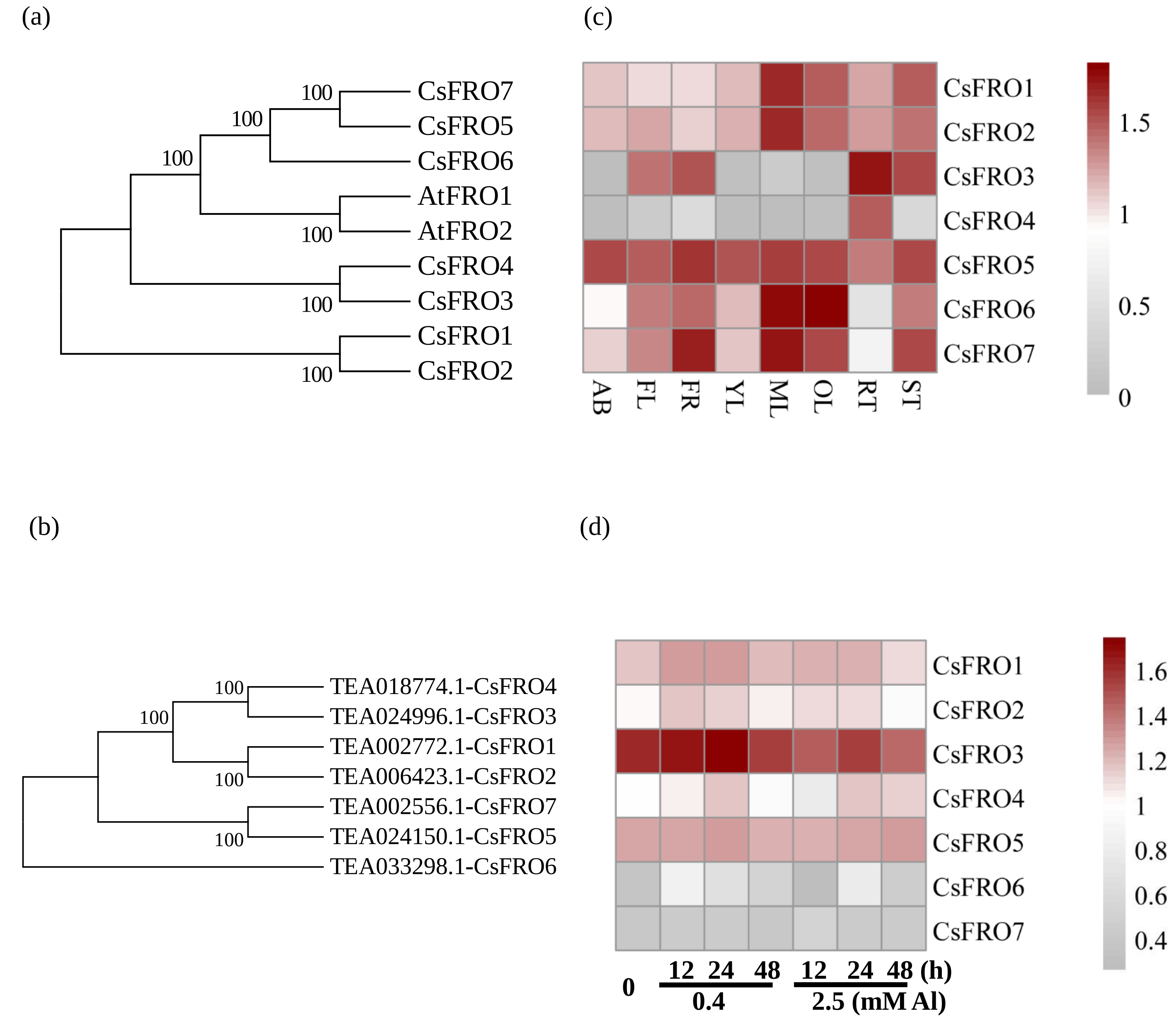

Fig S9 Identification and expression patterns of ferric reduction oxidase (*CsFRO*) gene family in *C. sinensis*.
(a) Phylogenetic analysis of ferric reduction oxidase (*CsFRO*) genes homology to functional characterized ones in Arabidopsis
(b) Annotation of ferric reduction oxidase (*CsFRO*) genes in *C. sinensis*.
(c) Expression patterns of ferric reduction oxidase (*CsFRO*) genes in various tissues of tea plants
(d) Expression patterns of ferric reduction oxidase (*CsFRO*) genes in tea plant roots in response to Al stress for various times

Zinc transporter (*CsZIP*)

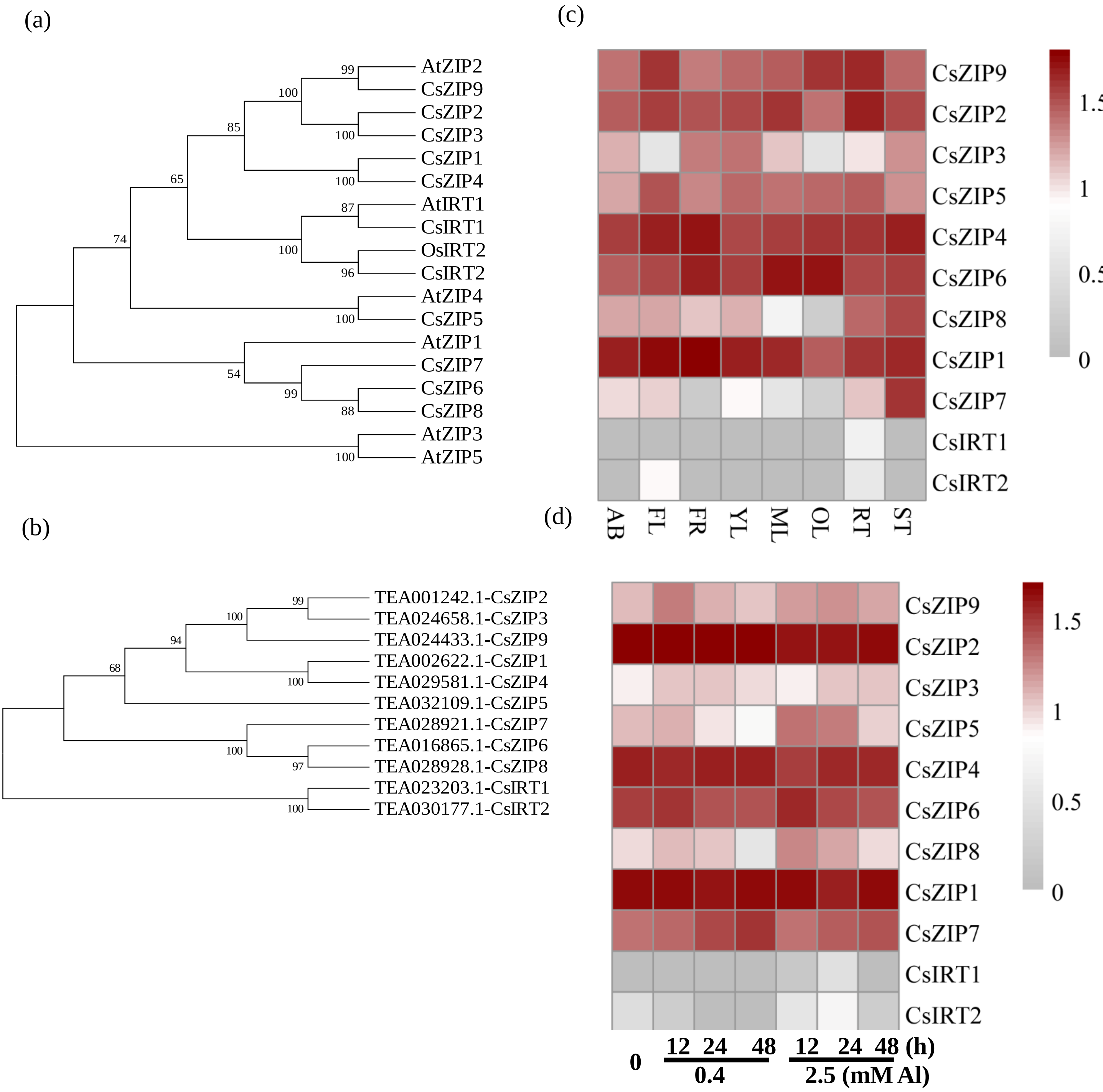

Fig S10 Identification and expression patterns of Zinc transporter (*CsZIP*) gene family in *C.sinensis.*

(a) Phylogenetic analysis of Zinc transporter (*CsZIP*) genes homology to functional characterized ones in Arabidopsis

(b) Annotation of Zinc transporter (*CsZIP*) genes in *C. sinensis*.

(c) Expression patterns of Zinc transporter (*CsZIP*) genes in various tissues of tea plants

(d) Expression patterns of Zinc transporter (*CsZIP*) genes in tea plant roots in response to Al stress for various times

Vacuolar iron transporter (*CsVIT*)

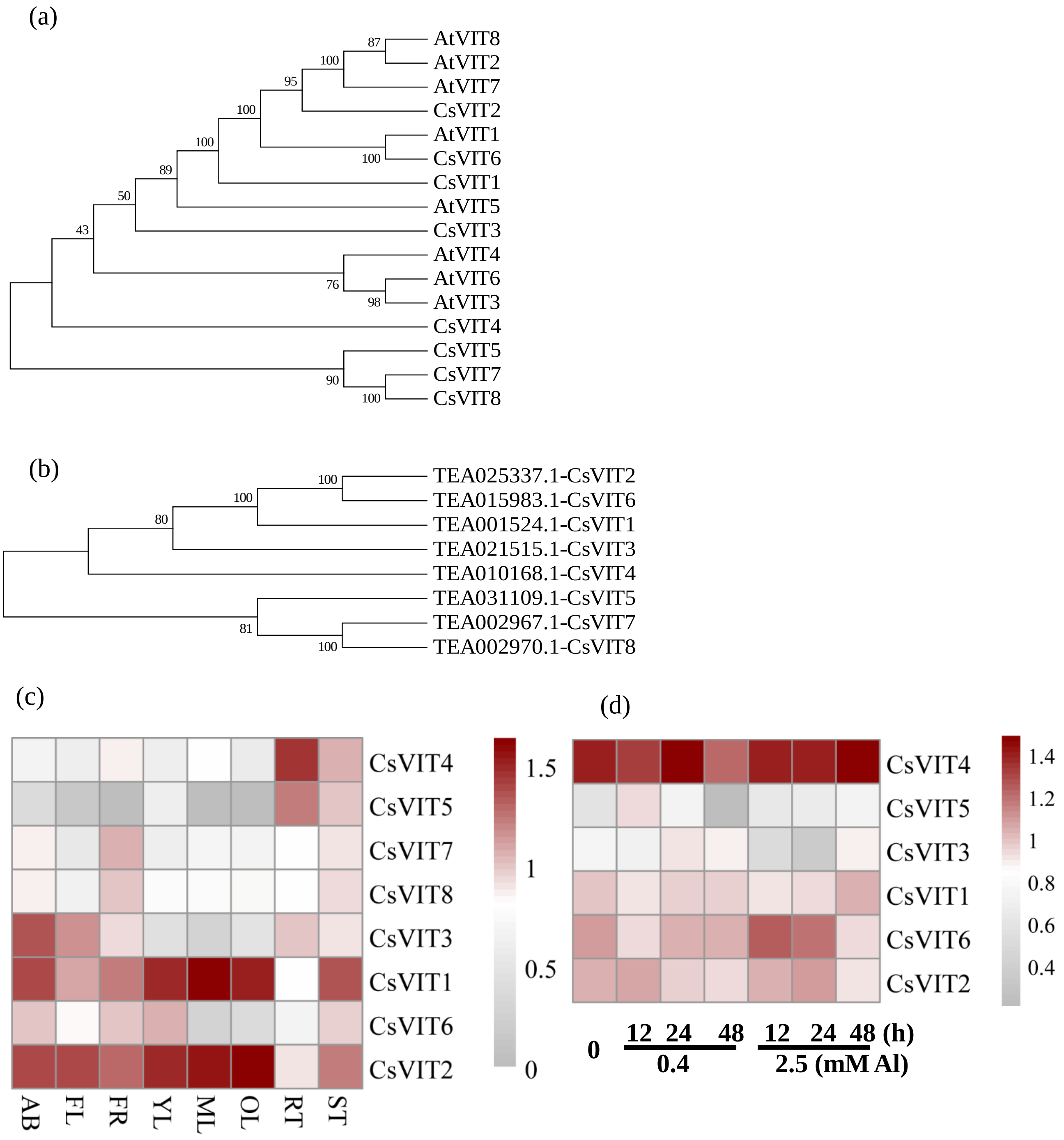

Fig S11 Identification and expression patterns of Vacuolar iron transporter (*CsVIT*) gene family in *C.sinensis*.

(a) Phylogenetic analysis of Vacuolar iron transporter (*CsVIT*) genes homology to functional characterized ones in Arabidopsis

(b) Annotation of Vacuolar iron transporter (*CsVIT*) genes in *C. sinensis*.

(c) Expression patterns of Vacuolar iron transporter (*CsVIT*) genes in various tissues of tea plants

(d) Expression patterns of Vacuolar iron transporter (*CsVIT*) genes in tea plant roots in response to Al stress for various times

natural resistance-associated macrophage proteins (*CsNramp*)

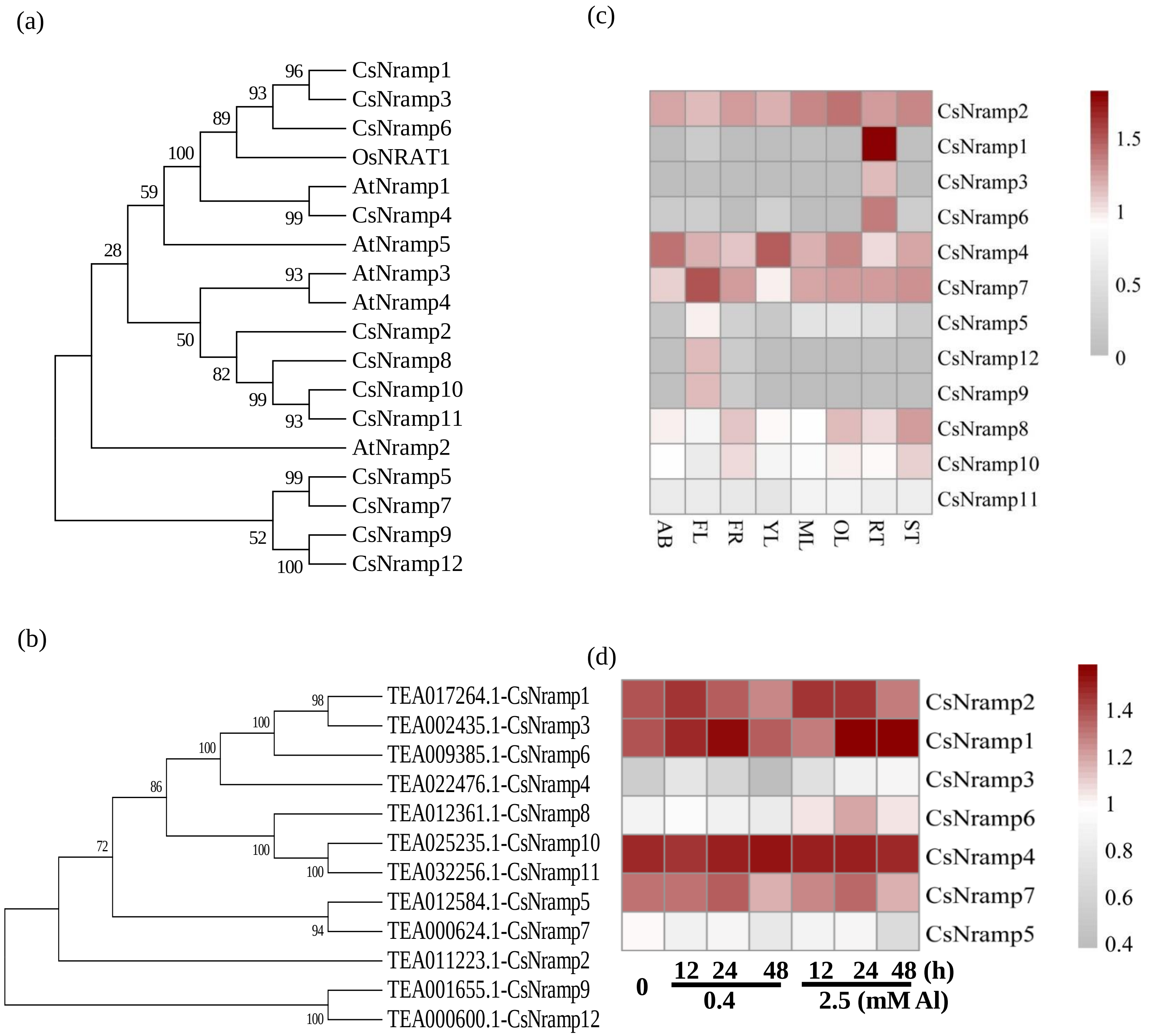

Fig S12 Identification and expression patterns of natural resistance-associated macrophage proteins (*CsNramp*) gene family in *C. sinensis*.
(a) Phylogenetic analysis of natural resistance-associated macrophage proteins (*CsNramp*) genes homology to functional characterized ones in Arabidopsis
(b) Annotation of natural resistance-associated macrophage proteins (*CsNramp*) genes in *C. sinensis*.
(c) Expression patterns of natural resistance-associated macrophage proteins (*CsNramp*) genes in various tissues of tea plants
(d) Expression patterns of natural resistance-associated macrophage proteins (*CsNramp*) genes in tea plant roots in response to Al stress for various times

oligopeptide transporter (*CsOPT*)

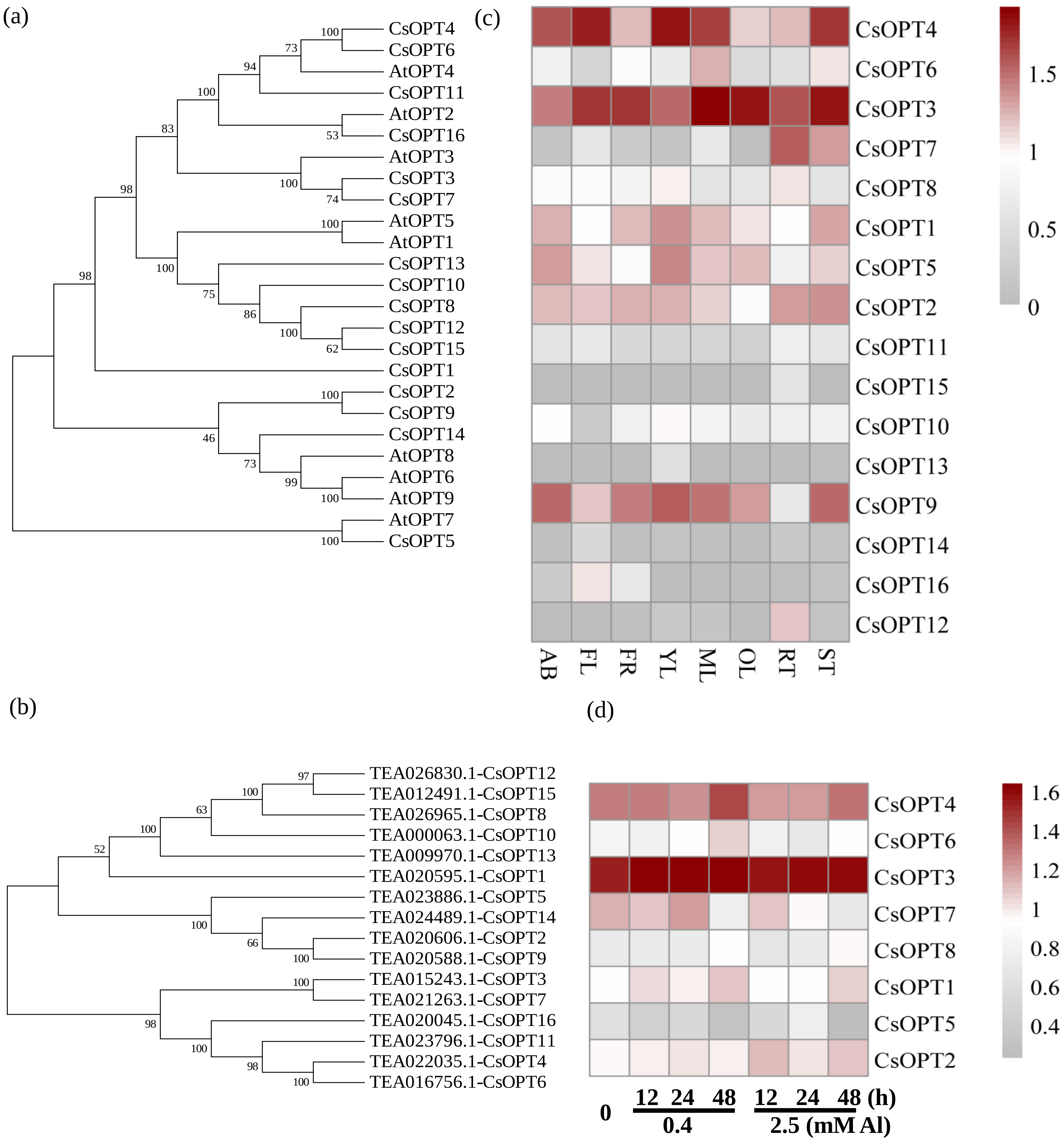

Fig S13 Identification and expression patterns of oligopeptide transporter (*CsOPT*) gene family in *C. sinensis*.

(a) Phylogenetic analysis of oligopeptide transporter (*CsOPT*) genes homology to functional characterized ones in Arabidopsis

(b) Annotation of oligopeptide transporter (*CsOPT*) genes in *C. sinensis*.

(c) Expression patterns of oligopeptide transporter (*CsOPT*) genes in various tissues of tea plants

(d) Expression patterns of oligopeptide transporter (*CsOPT*) genes in tea plant roots in response to Al stress for various times

Metal-nicotianamine transporter (*CsYSL*)

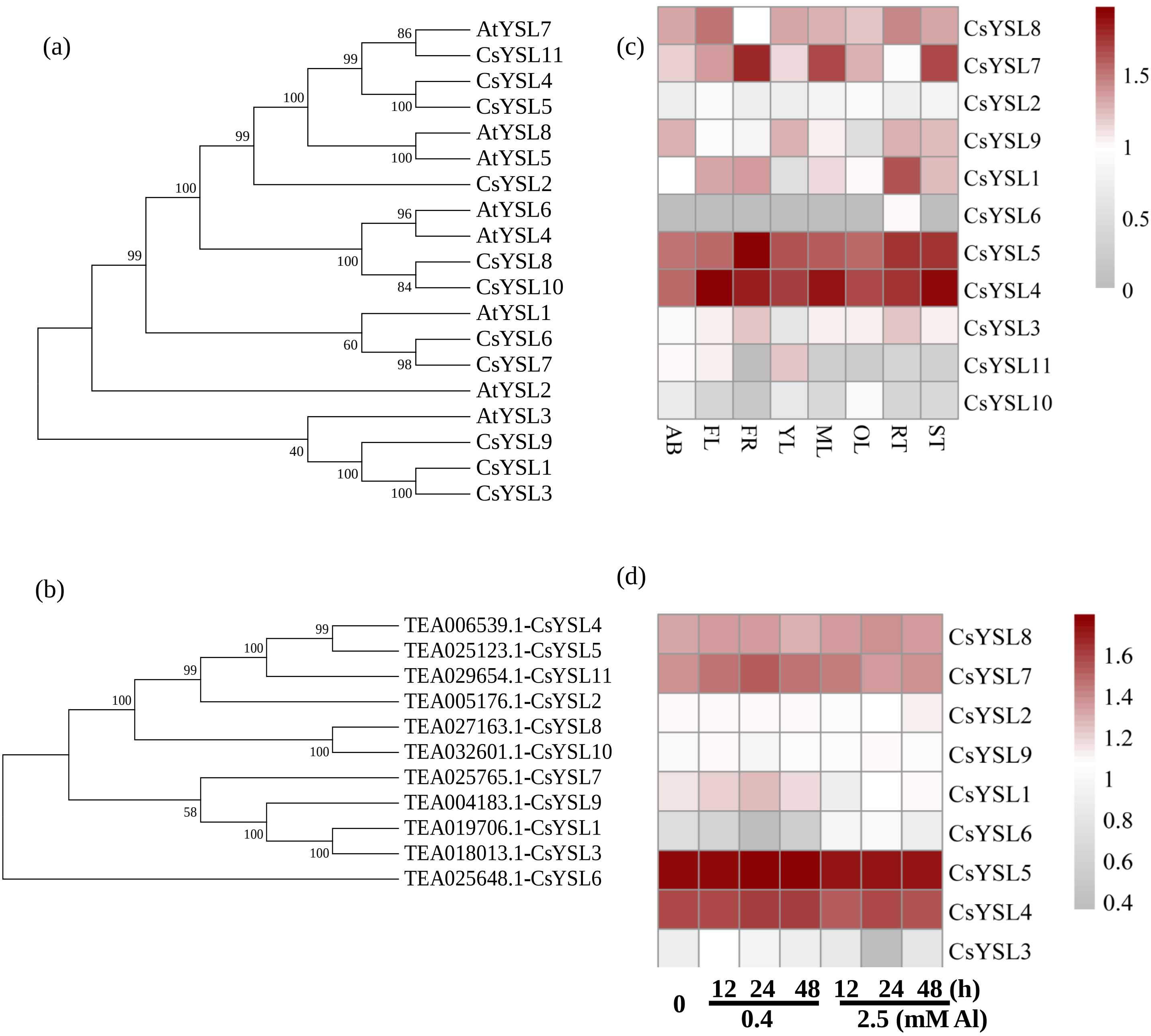

Fig S14 Identification and expression patterns of Metal-nicotianamine transporter (*CsYSL*) gene family in *C. sinensis*.

(a)Phylogenetic analysis of Metal-nicotianamine transporter (*CsYSL*) genes homology to functional characterized ones in Arabidopsis

(b)Annotation of Metal-nicotianamine transporter (*CsYSL*) genes in *C. sinensis.*

(c)Expression patterns of Metal-nicotianamine transporter (*CsYSL*) genes in various tissues of tea plants

(d)Expression patterns of Metal-nicotianamine transporter (*CsYSL*) genes in tea plant roots in response to Al stress for various times

ferretin (*CsFer*)

(a)

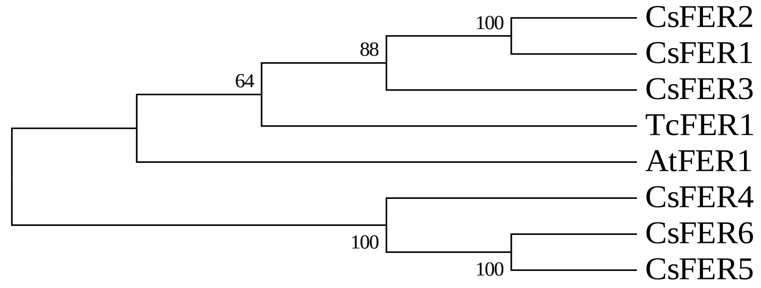

(b)

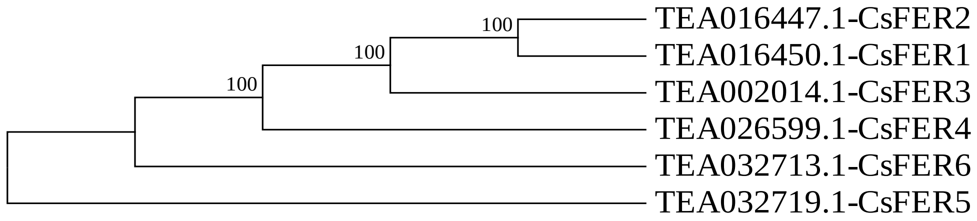

(c)

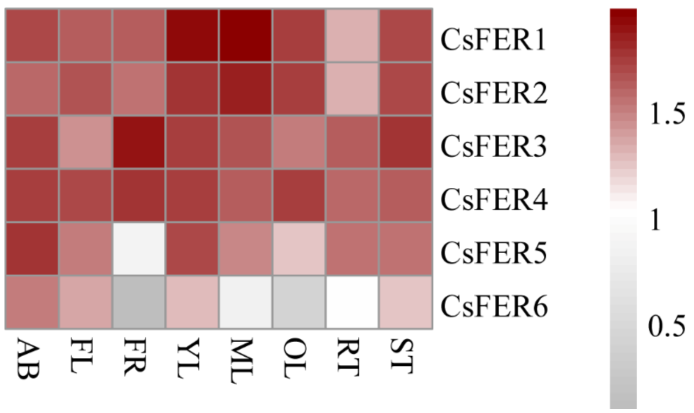

(d)

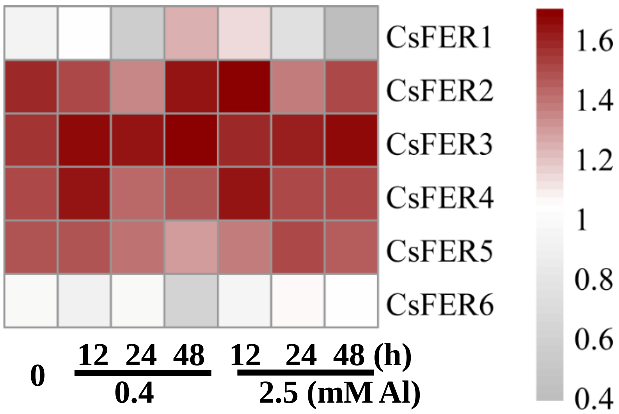

Fig S15 Identification and expression patterns of ferretin (*CsFer*) gene family in *C.sinensis*.
(a) Phylogenetic analysis of ferretin (*CsFer*) genes homology to functional characterized ones in Arabidopsis
(b) Annotation of ferretin (*CsFer*) genes in *C. sinensis*.
(c) Expression patterns of ferretin (*CsFer*) genes in various tissues of tea plants
(d) Expression patterns of ferretin (*CsFer*) genes in tea plant roots in response to Al stress for various times

heavy metal-associated ATPase (*CsHMA*) and plasma membrane H+ ATPase (*CsPMA*)

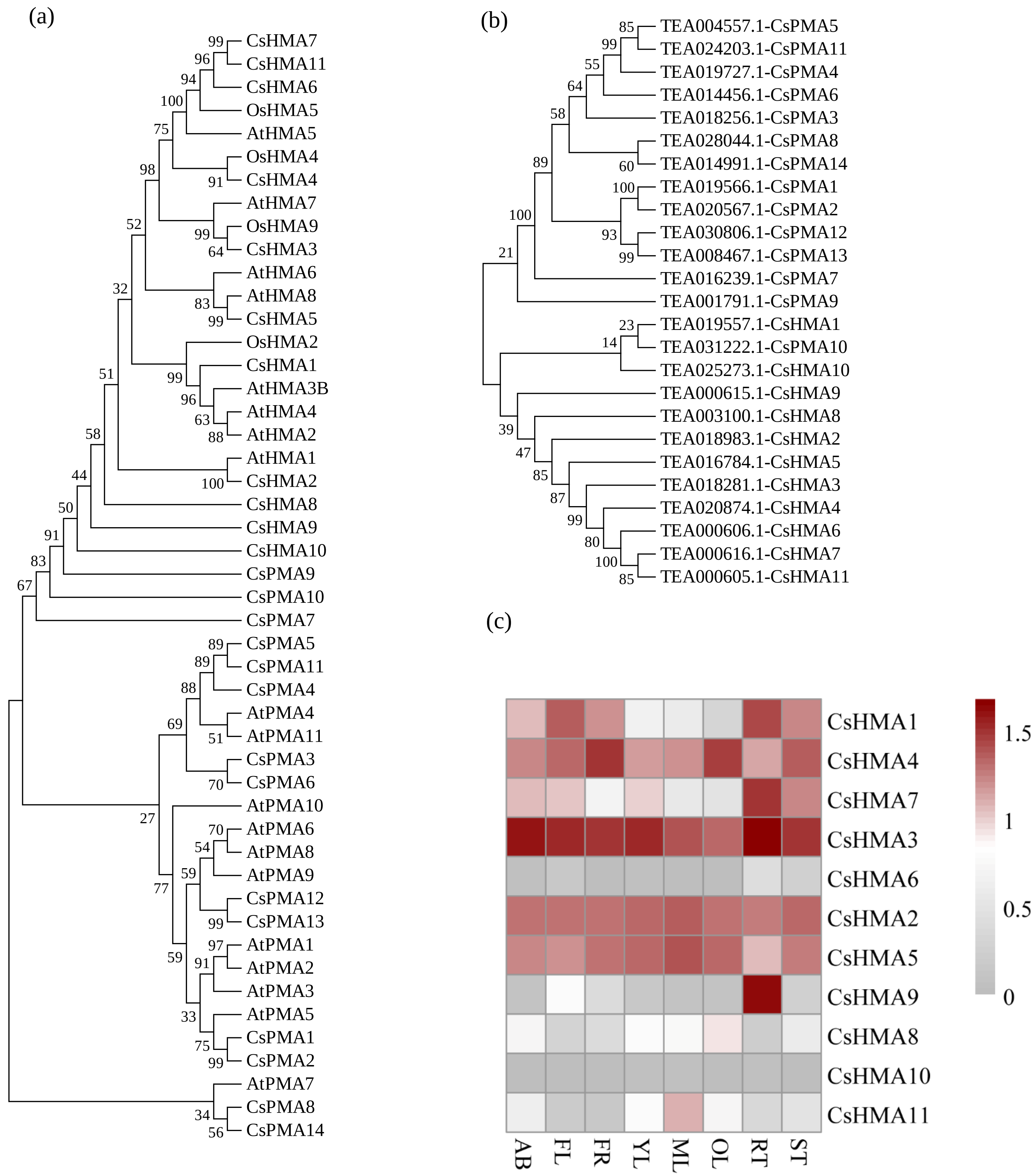

(d)

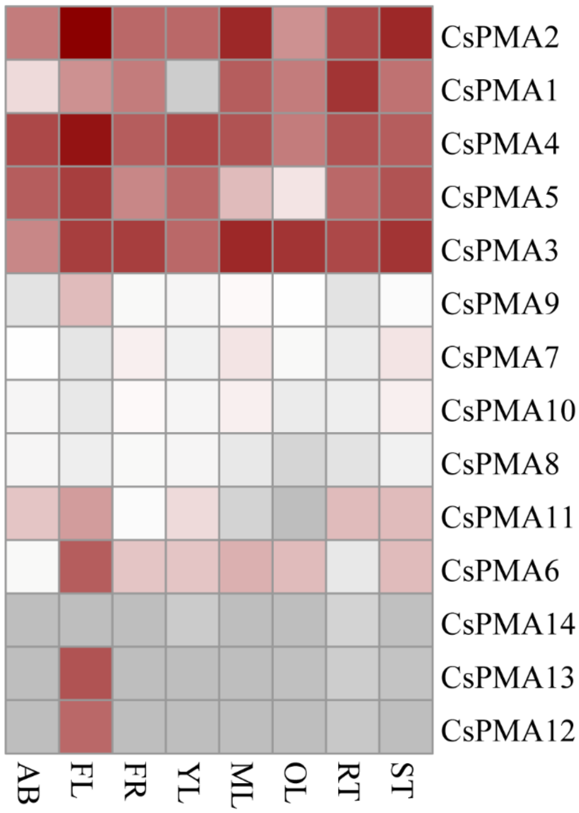

(e)

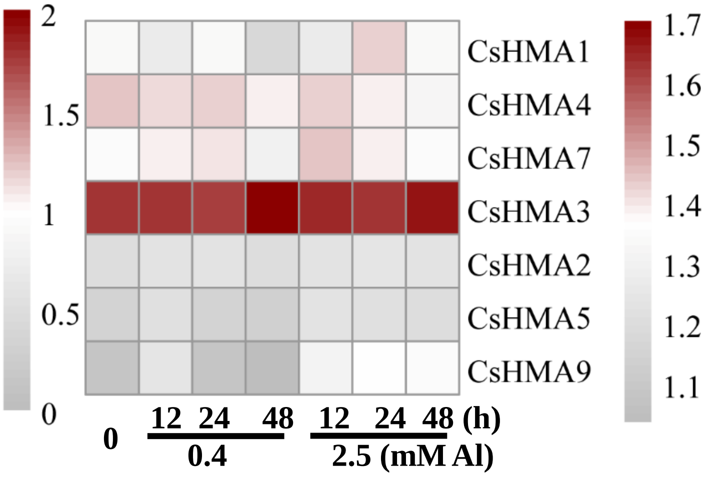

(f)

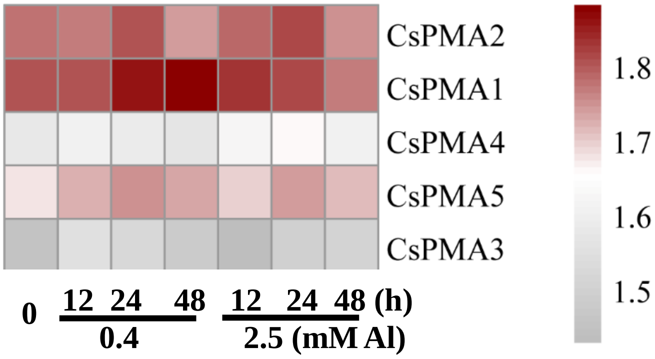

Fig S16 Identification and expression patterns of heavy metal-associated ATPase (*CsHMA*) and plasma membrane H+ ATPase (*CsPMA*) gene family in *C.sinensis.*

(a) Phylogenetic analysis of heavy metal-associated ATPase (HMA) and plasma membrane H+ ATPase (*CsPMA*)

genes homology to functional characterized ones in Arabidopsis

(b) Annotation of heavy metal-associated ATPase (*CsHMA*) and plasma membrane H+ ATPase (PMA) gene in *C. sinensis*.

(c)(d) Expression patterns of heavy metal-associated ATPase (*CsHMA*) and plasma membrane H+ ATPase (*CsPMA*) gene in various tissues of tea plants

(e)(f) Expression patterns of heavy metal-associated ATPase (*CsHMA*) and plasma membrane H+ ATPase (*CsPMA*) gene in tea plant roots in response to Al stress for various times

Cu Copper transporter (*CsCOPT*)

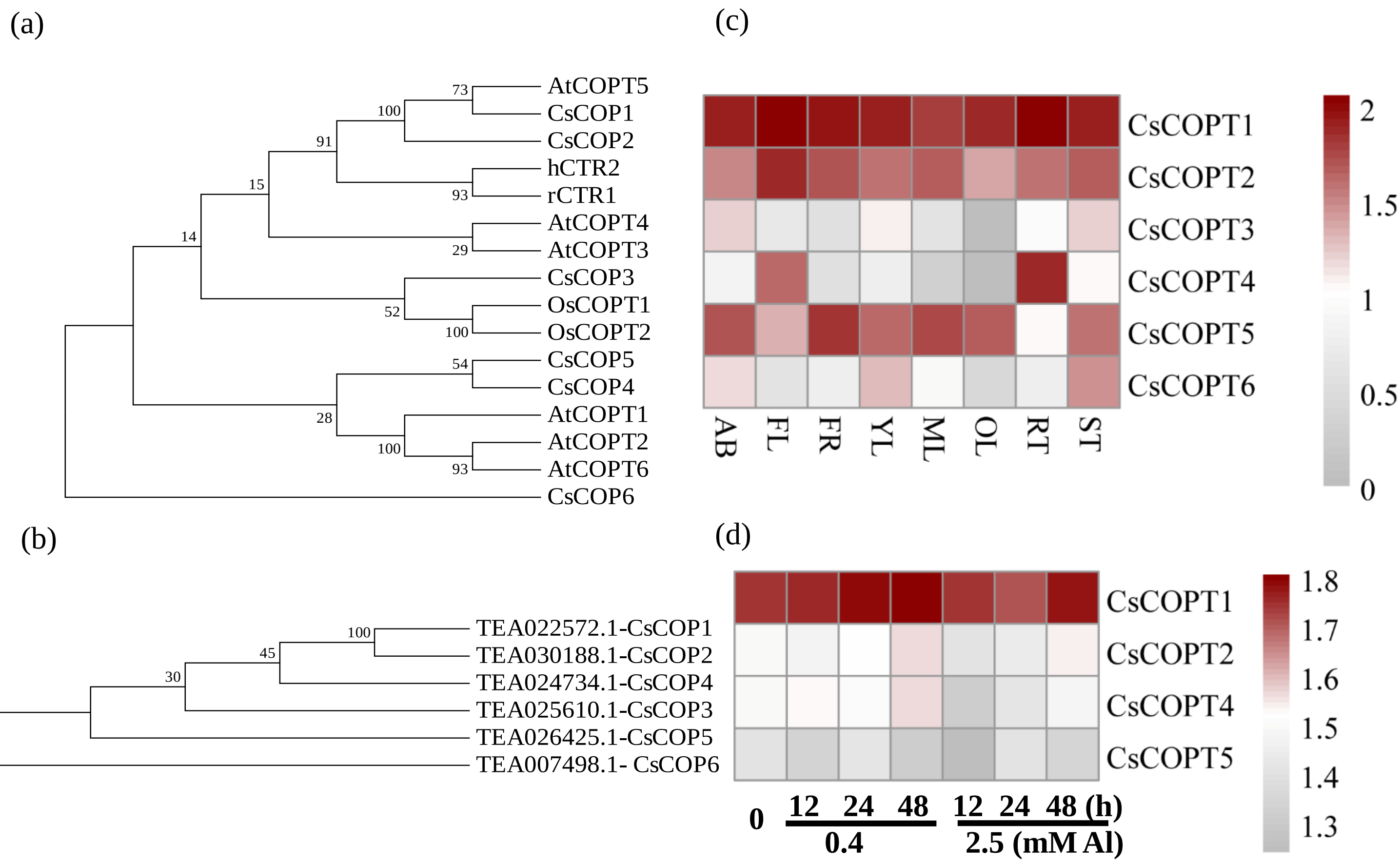

Fig S17 Identification and expression patterns of Copper transporter (*CsCOPT*) gene family in *C.sinensis.*
(a) Phylogenetic analysis of Copper transporter (*CsCOPT*) genes homology to functional characterized ones in Arabidopsis
(b) Annotation of Copper transporter (*CsCOPT*) genes in *C. sinensis*.
(c) Expression patterns of Copper transporter (*CsCOPT*) genes in various tissues of tea plants
(d) Expression patterns of Copper transporter (*CsCOPT*) genes in tea plant roots in response to Al stress for various times

Glutaredoxin (*CsGrx*)

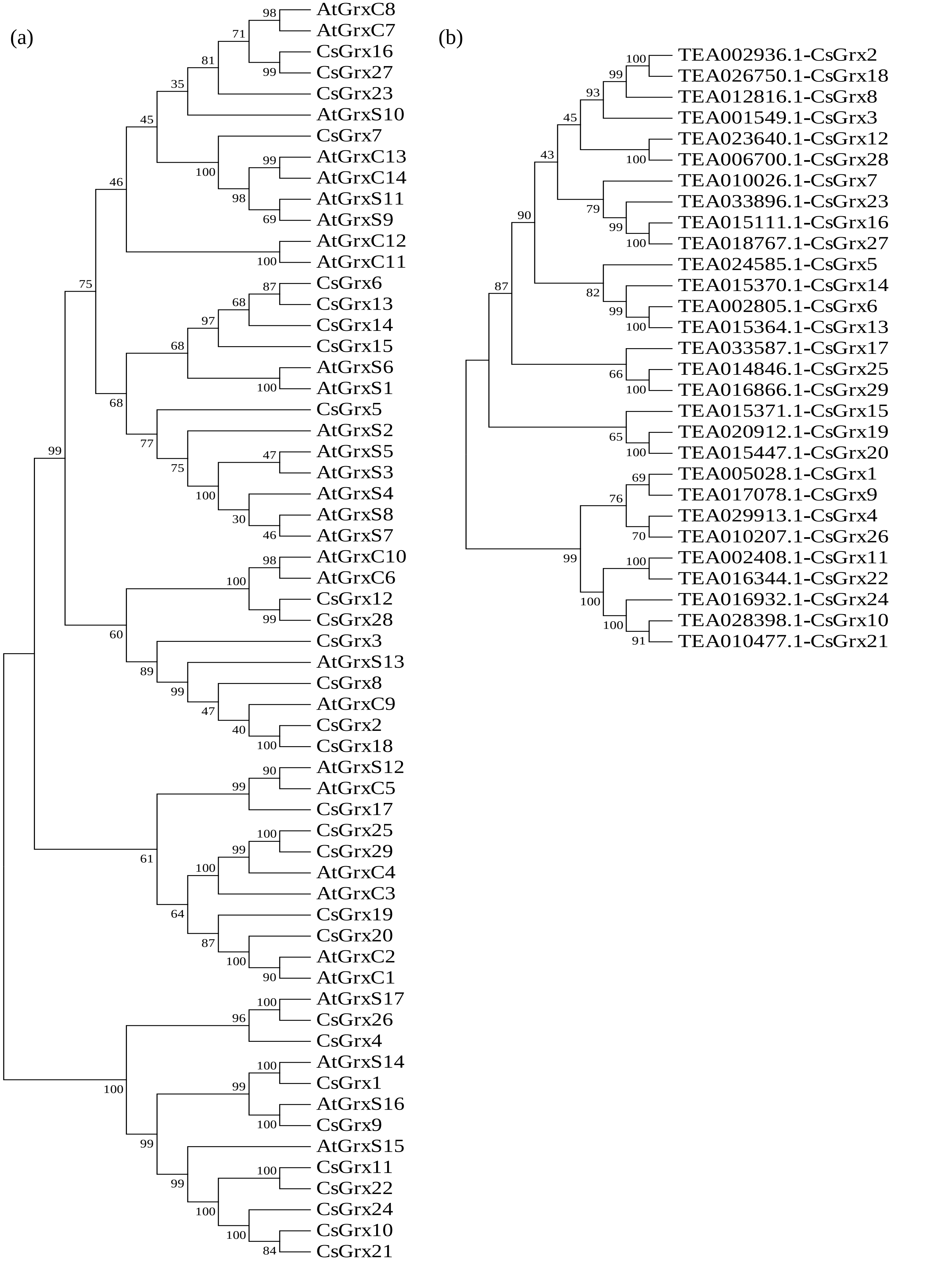

(c)

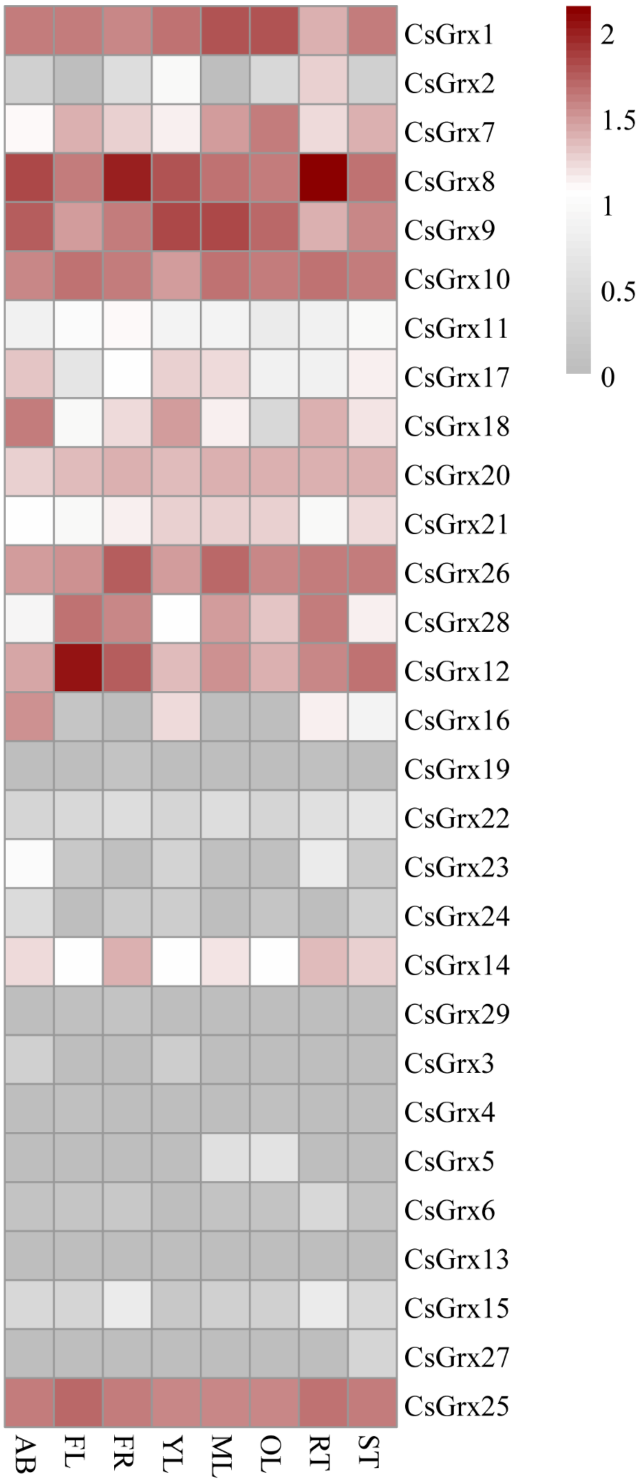

(d)

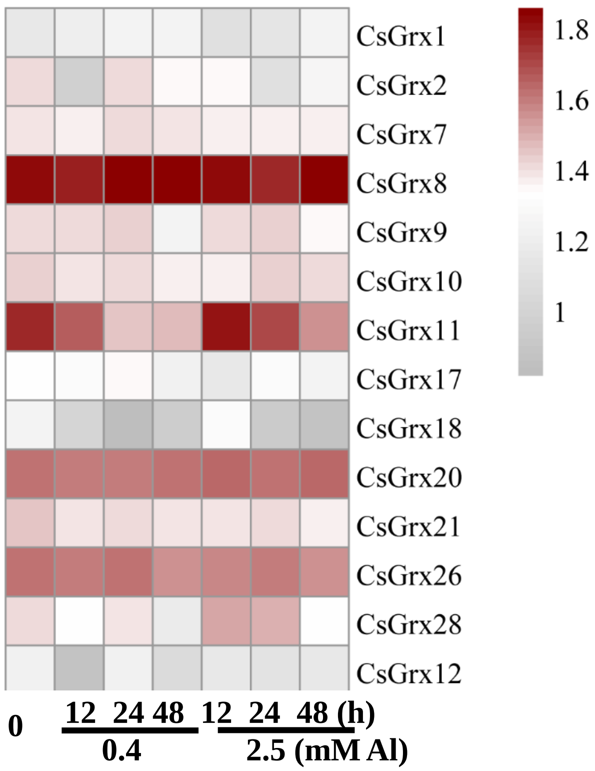

Fig S18 Identification and expression patterns of glutaredoxin (*CsGrx*) gene family in *C.sinensis*.
(a) Phylogenetic analysis of glutaredoxin (*CsGrx*) genes homology to functional characterized ones in Arabidopsis
(b) Annotation of glutaredoxin (*CsGrx*) genes in *C. sinensis.*
(c) Expression patterns of glutaredoxin (*CsGrx*) genes in various tissues of tea plants
(d) Expression patterns of glutaredoxin (*CsGrx*) genes in tea plant roots in response to Al stress for various times

thioredoxin (*CsTRX*)

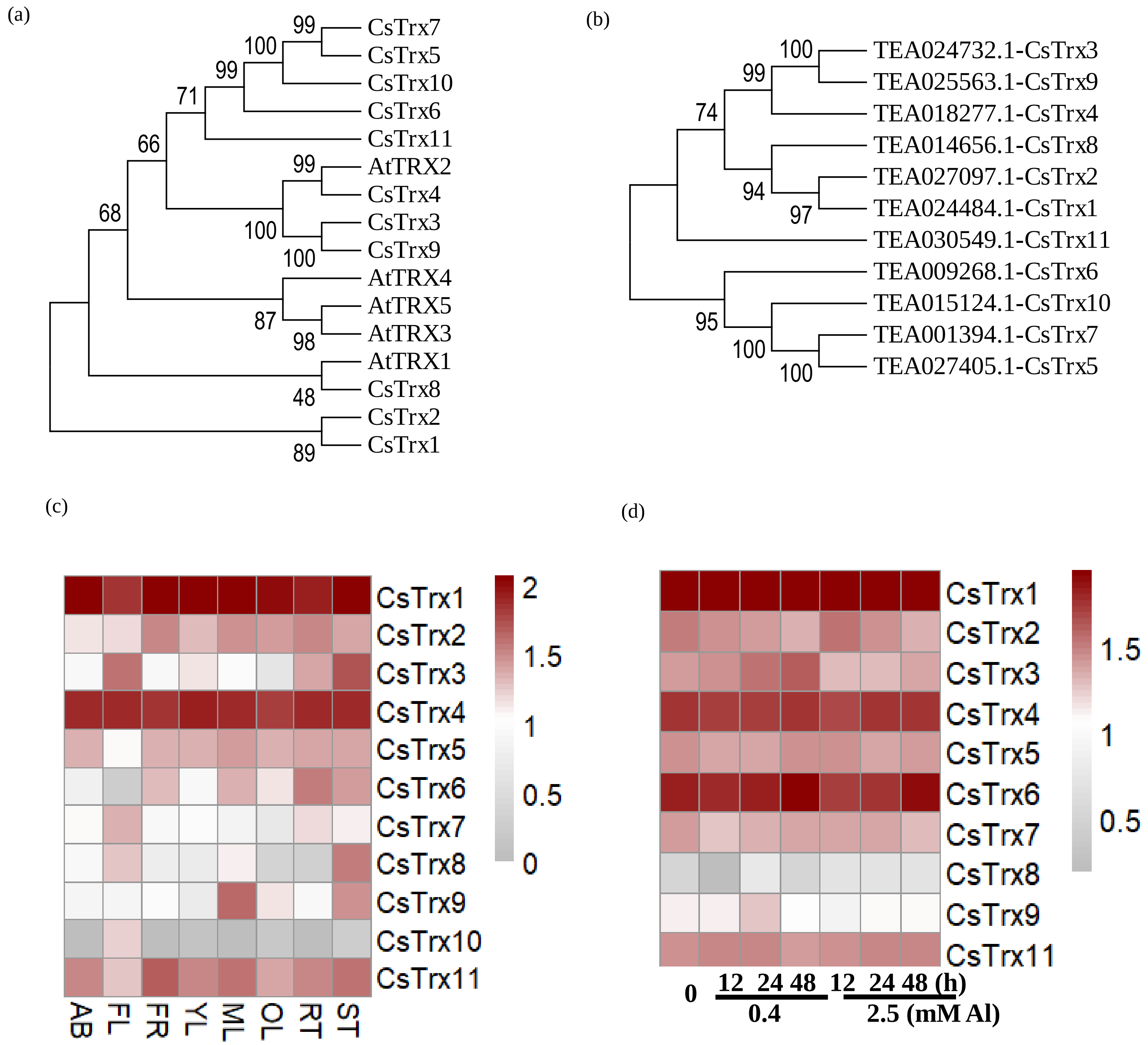

Fig S19 Identification and expression patterns of thioredoxin (*CsTRX*) gene family in *C. sinensis*.

(a) Phylogenetic analysis of thioredoxin (*CsTRX*) genes homology to functional characterized ones in Arabidopsis

(b) Annotation of thioredoxin (*CsTRX*) genes in *C. sinensis*.

(c) Expression patterns of thioredoxin (*CsTRX*) genes in various tissues of tea plants

(d) Expression patterns of thioredoxin (*CsTRX*) genes in tea plant roots in response to Al stress for various times

glutathione S-transferase (*CsGST*)

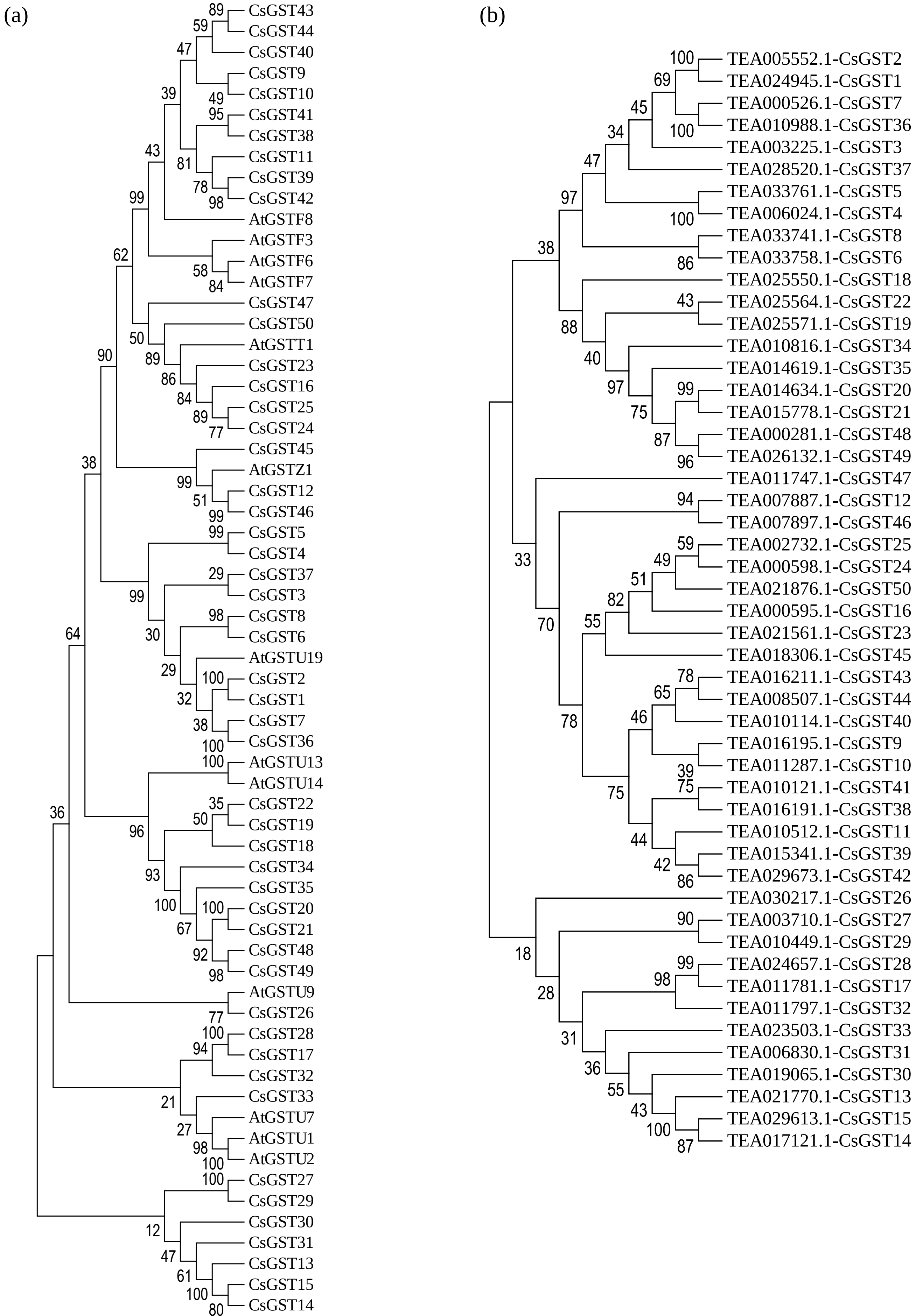

(c)

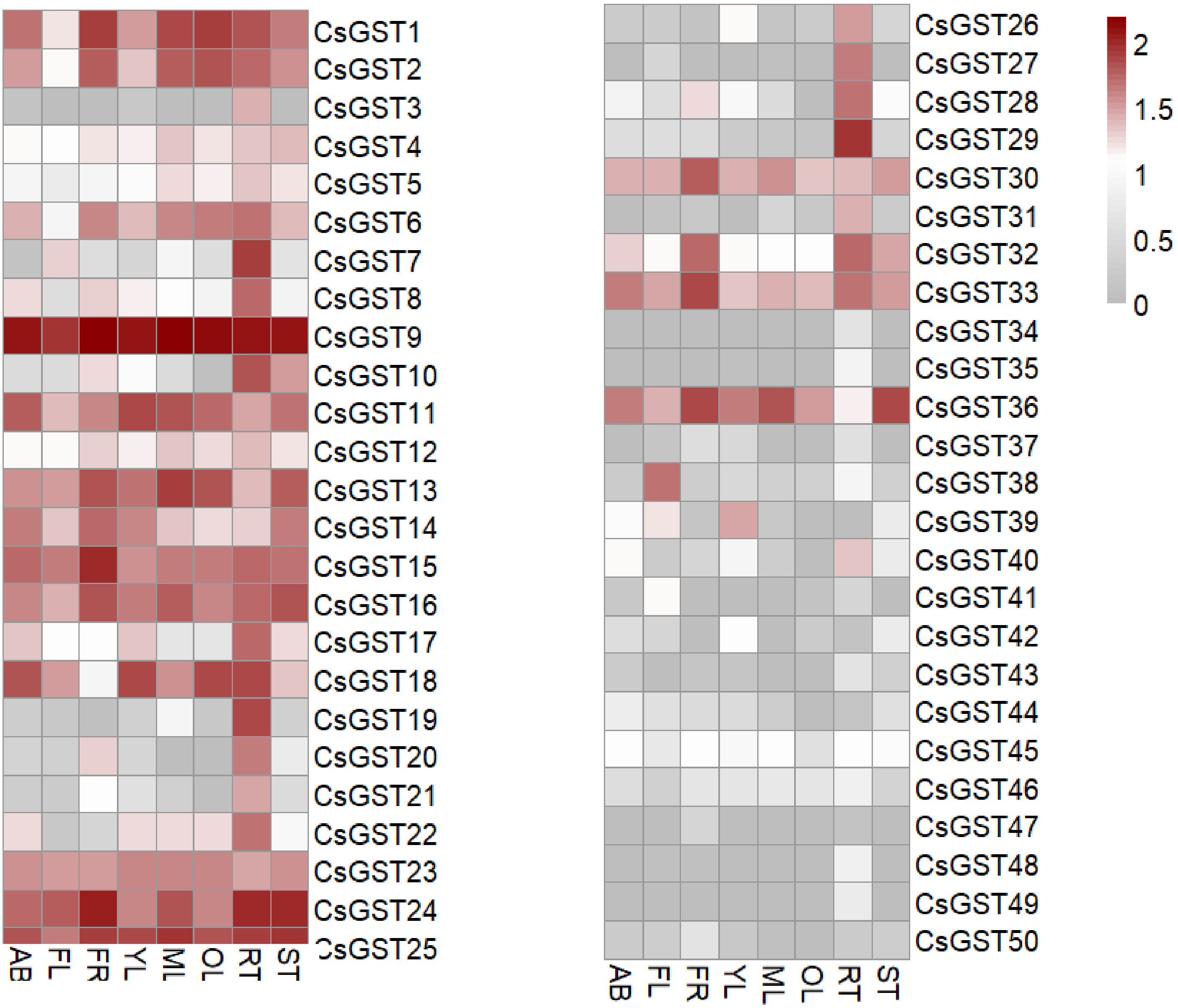
CsGST1
CsGST2
CsGST3
CsGST4
CsGST5
CsGST6
CsGST7
CsGST8
CsGST9
CsGST10
CsGST11
CsGST12
CsGST13
CsGST14
CsGST15
CsGST16
CsGST17
CsGST18
CsGST19
CsGST20
CsGST21
CsGST22
CsGST23
CsGST24
CsGST25
AB
FL
FR
YL
ML
OL
RT
ST
CsGST26
CsGST27
CsGST28
CsGST29
CsGST30
CsGST31
CsGST32
CsGST33
CsGST34
CsGST35
CsGST36
CsGST37
CsGST38
CsGST39
CsGST40
CsGST41
CsGST42
CsGST43
CsGST44
CsGST45
CsGST46
CsGST47
CsGST48
CsGST49
CsGST50
AB
FL
FR
YL
ML
OL
RT
ST
2
1.5
1
0.5
0

(d)

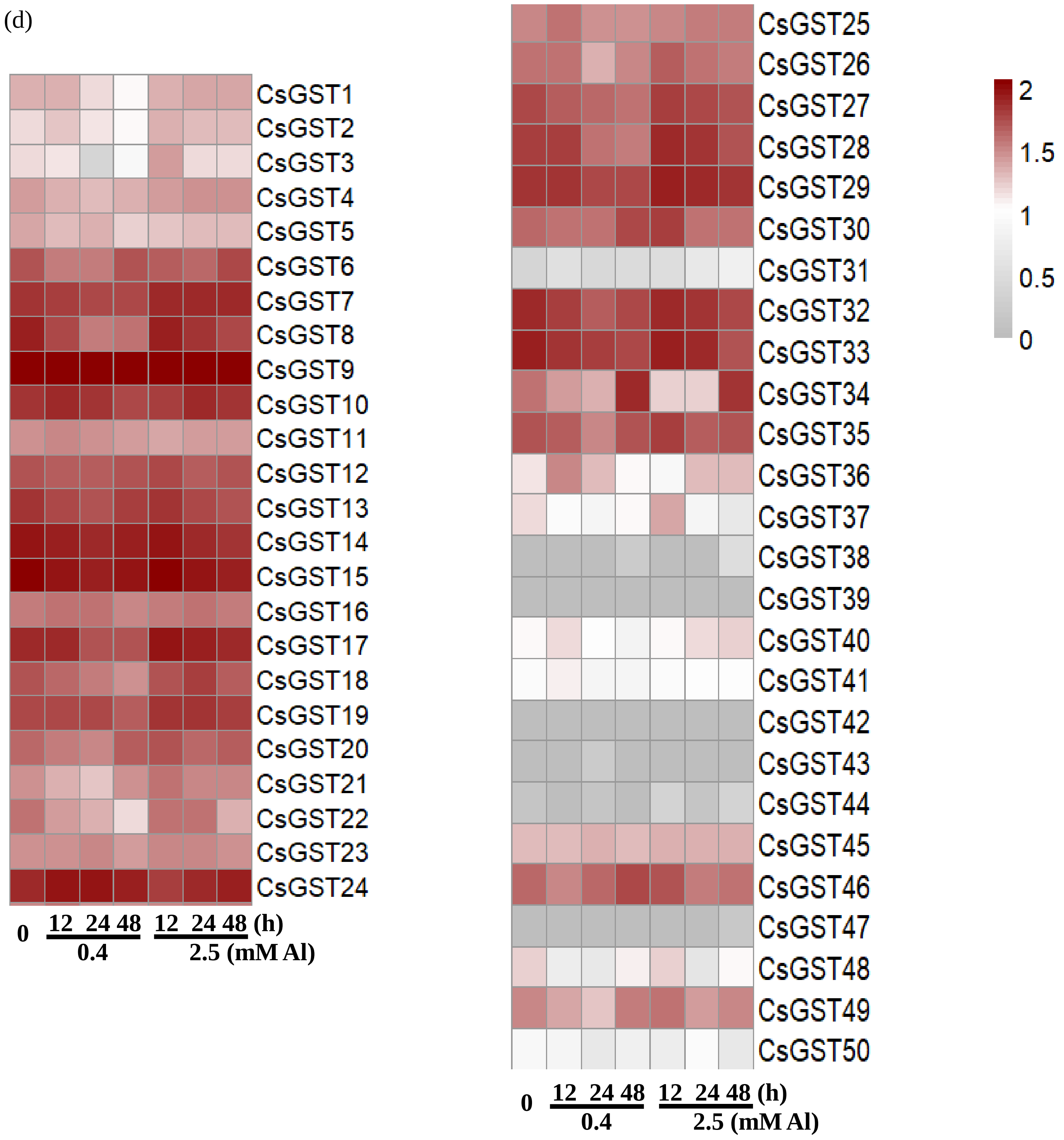

Fig S20 Identification and expression patterns of glutathione S-transferase (*CsGST*) gene family in *C. sinensis*.

(a) Phylogenetic analysis of glutathione S-transferase (*CsGST*) genes homology to functional characterized ones in Arabidopsis

(b) Annotation of glutathione S-transferase (*CsGST*) genes in *C. sinensis*.

(c) Expression patterns of glutathione S-transferase (*CsGST*) genes in various tissues of tea plants

(d) Expression patterns of glutathione S-transferase (*CsGST*) genes in tea plant roots in response to Al stress for various times

Aquaporin (*CsNIP*)

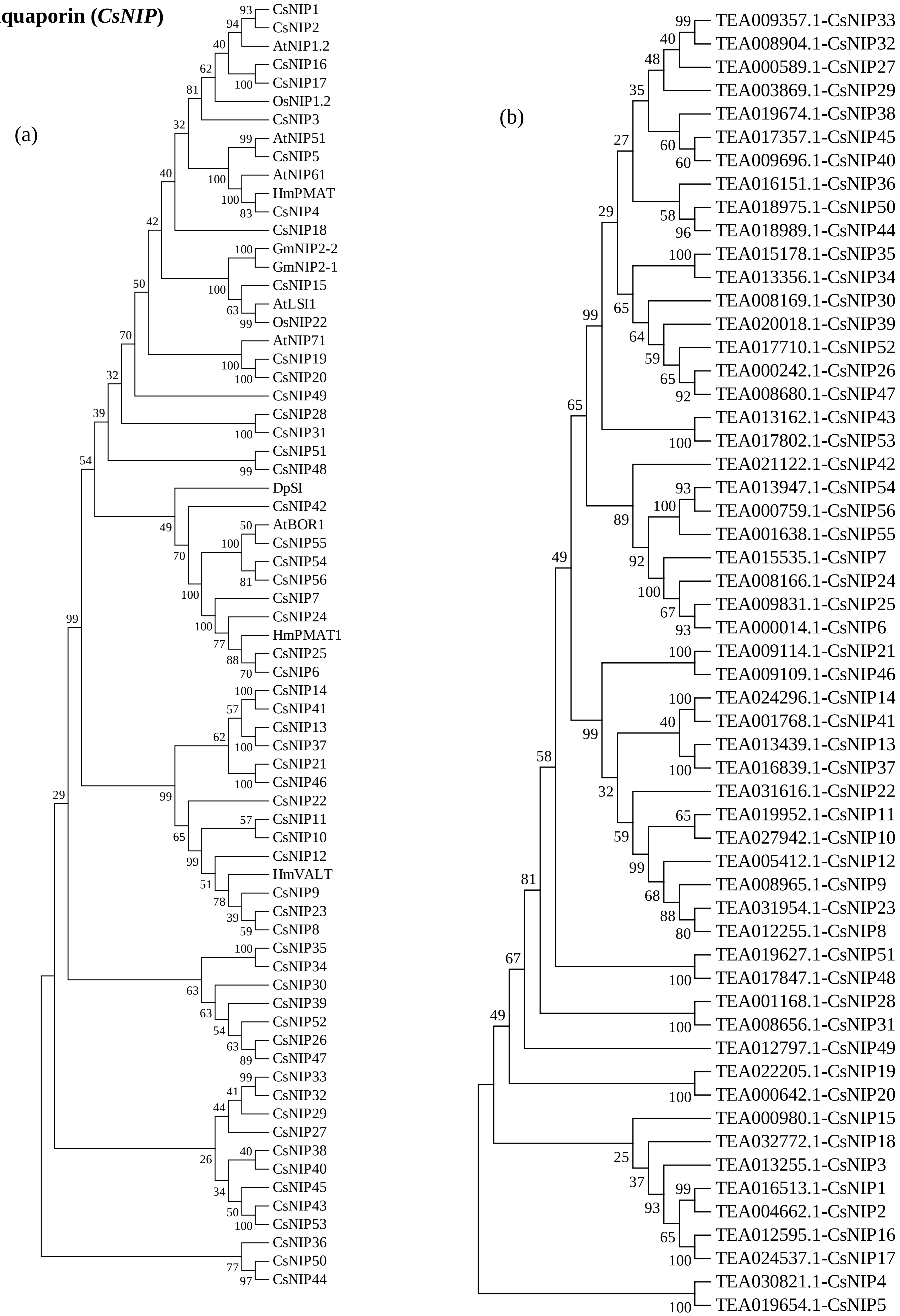

(c)

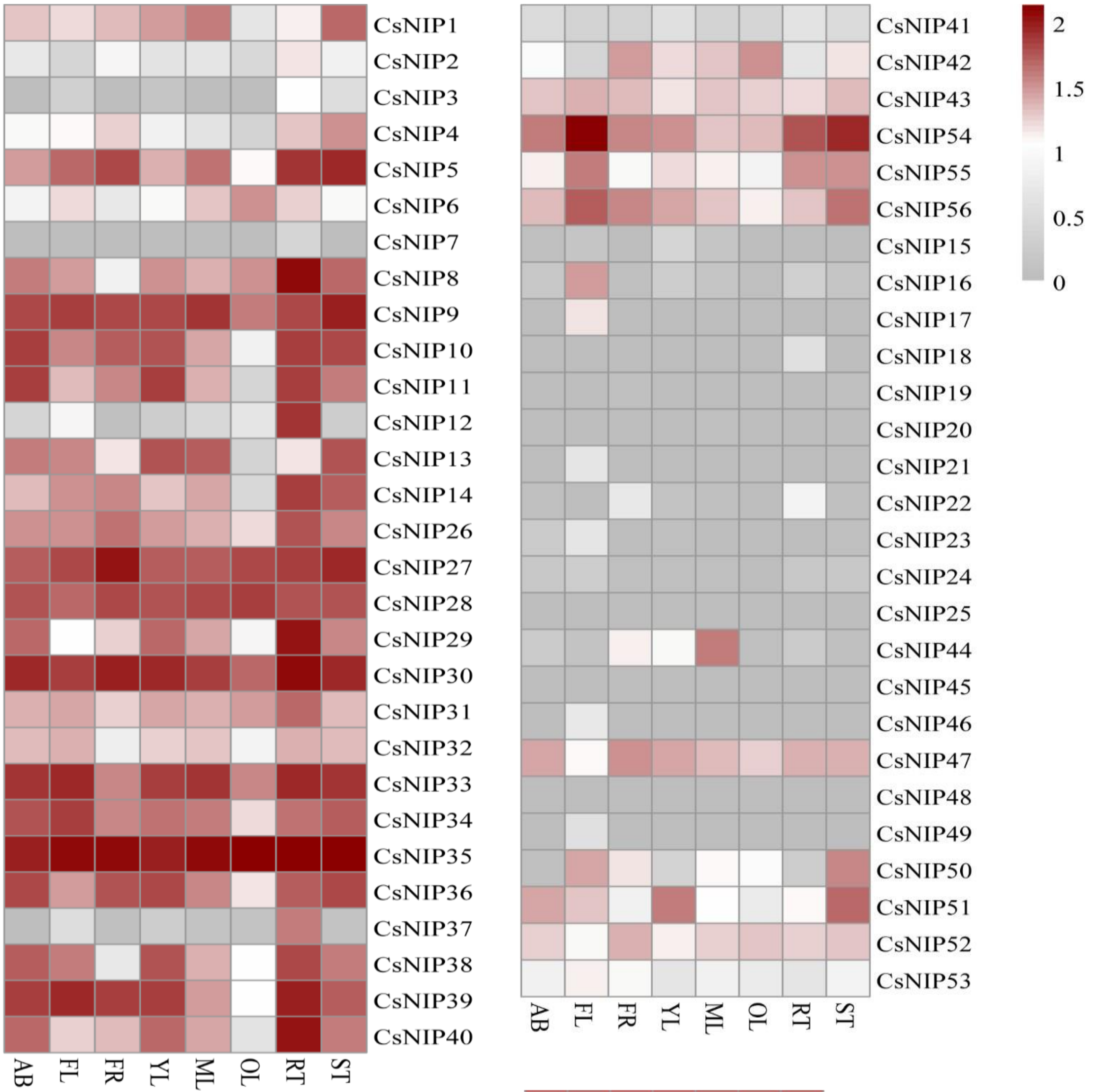
CsNIP1
CsNIP2
CsNIP3
CsNIP4
CsNIP5
CsNIP6
CsNIP7
CsNIP8
CsNIP9
CsNIP10
CsNIP11
CsNIP12
CsNIP13
CsNIP14
CsNIP26
CsNIP27
CsNIP28
CsNIP29
CsNIP30
CsNIP31
CsNIP32
CsNIP33
CsNIP34
CsNIP35
CsNIP36
CsNIP37
CsNIP38
CsNIP39
CsNIP40
AB FL FR YL ML OL RT ST
CsNIP41
CsNIP42
CsNIP43
CsNIP54
CsNIP55
CsNIP56
CsNIP15
CsNIP16
CsNIP17
CsNIP18
CsNIP19
CsNIP20
CsNIP21
CsNIP22
CsNIP23
CsNIP24
CsNIP25
CsNIP44
CsNIP45
CsNIP46
CsNIP47
CsNIP48
CsNIP49
CsNIP50
CsNIP51
CsNIP52
CsNIP53
AB FL FR YL ML OL RT ST
2
1.5
1
0.5
0

(d)

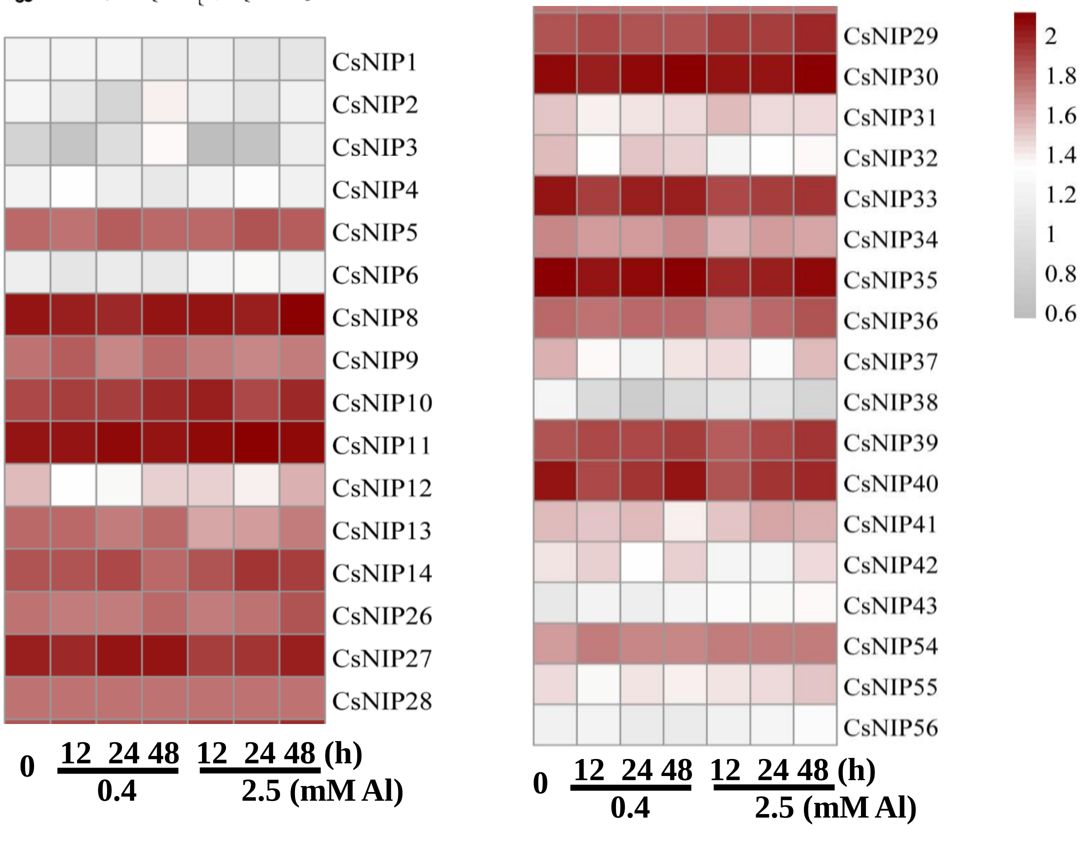
CsNIP1
CsNIP2
CsNIP3
CsNIP4
CsNIP5
CsNIP6
CsNIP8
CsNIP9
CsNIP10
CsNIP11
CsNIP12
CsNIP13
CsNIP14
CsNIP26
CsNIP27
CsNIP28
0 12 24 48 12 24 48 (h)
0.4 2.5 (mM Al)
CsNIP29
CsNIP30
CsNIP31
CsNIP32
CsNIP33
CsNIP34
CsNIP35
CsNIP36
CsNIP37
CsNIP38
CsNIP39
CsNIP40
CsNIP41
CsNIP42
CsNIP43
CsNIP54
CsNIP55
CsNIP56
0 12 24 48 12 24 48 (h)
0.4 2.5 (mM Al)
2
1.8
1.6
1.4
1.2
1
0.8
0.6

Fig S21 Identification and expression patterns of Aquaporin (*CsNIP*) gene family in *C. sinensis*.

(a)Phylogenetic analysis of Aquaporin (*CsNIP*) genes homology to functional characterized ones in Arabidopsis

(b)Annotation of Aquaporin (*CsNIP*) genes in *C. sinensis*.

(c)Expression patterns ofAquaporin (*CsNIP*) genes in various tissues of tea plants

(d)Expression patterns of Aquaporin (*CsNIP*) genes in tea plant roots in response to Al stress for various times

Al Aluminum sensitive (*CsALS*)-like

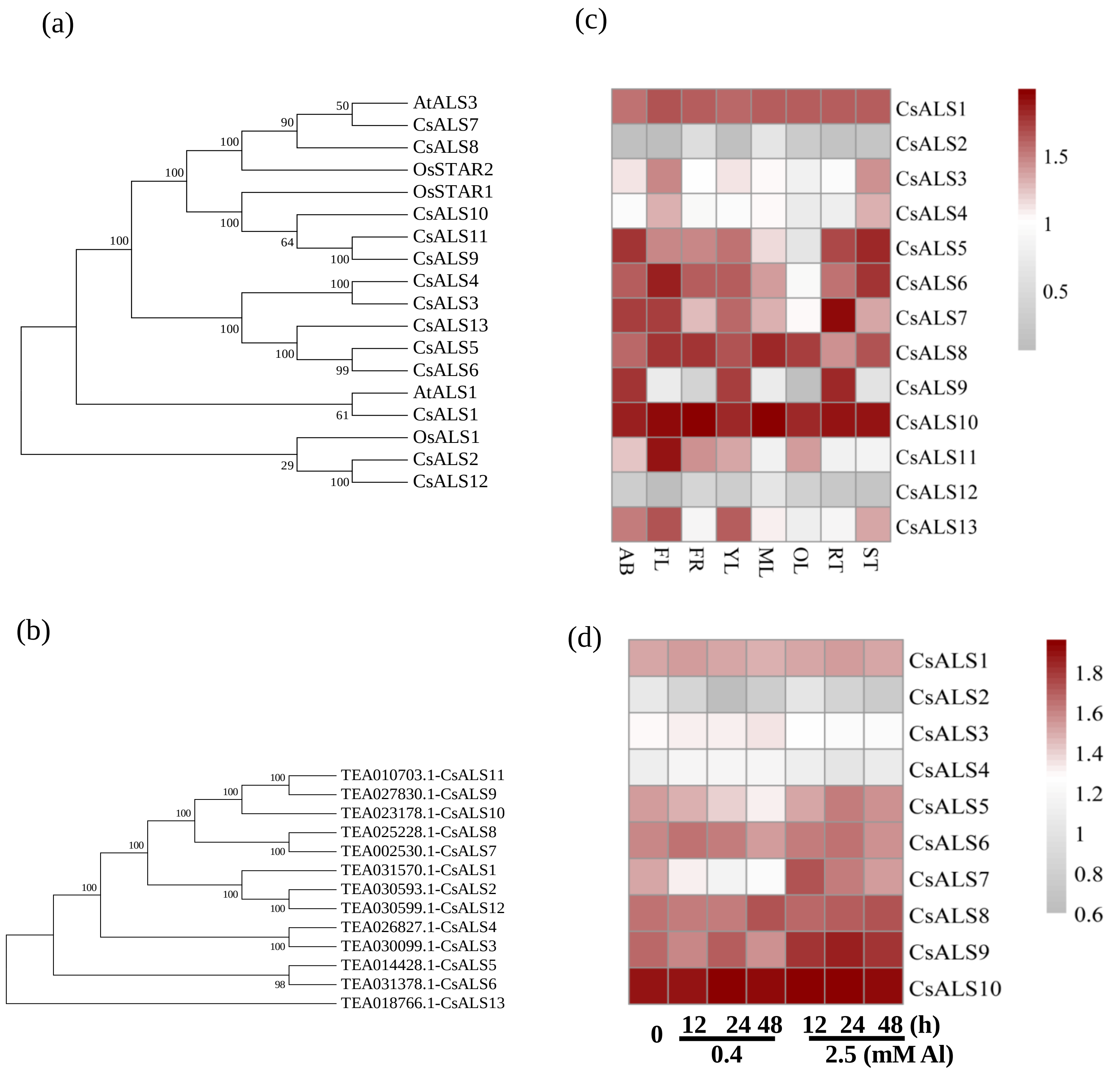

Fig S22 Identification and expression patterns of Protein aluminum sensitive (*CsALS*) gene family in *C.sinensis*.
(a) Phylogenetic analysis of Protein aluminum sensitive (*CsALS*) genes homology to functional characterized ones in Arabidopsis
(b) Annotation of Protein aluminum sensitive (*CsALS*) genes in *C. sinensis*.
(c) Expression patterns of Protein aluminum sensitive (*CsALS*) genes in various tissues of tea plants
(d) Expression patterns of Protein aluminum sensitive (*CsALS*) genes in tea plant roots in response to Al stress for various times

Aluminum-activated malate transporter (*CsALMT*)

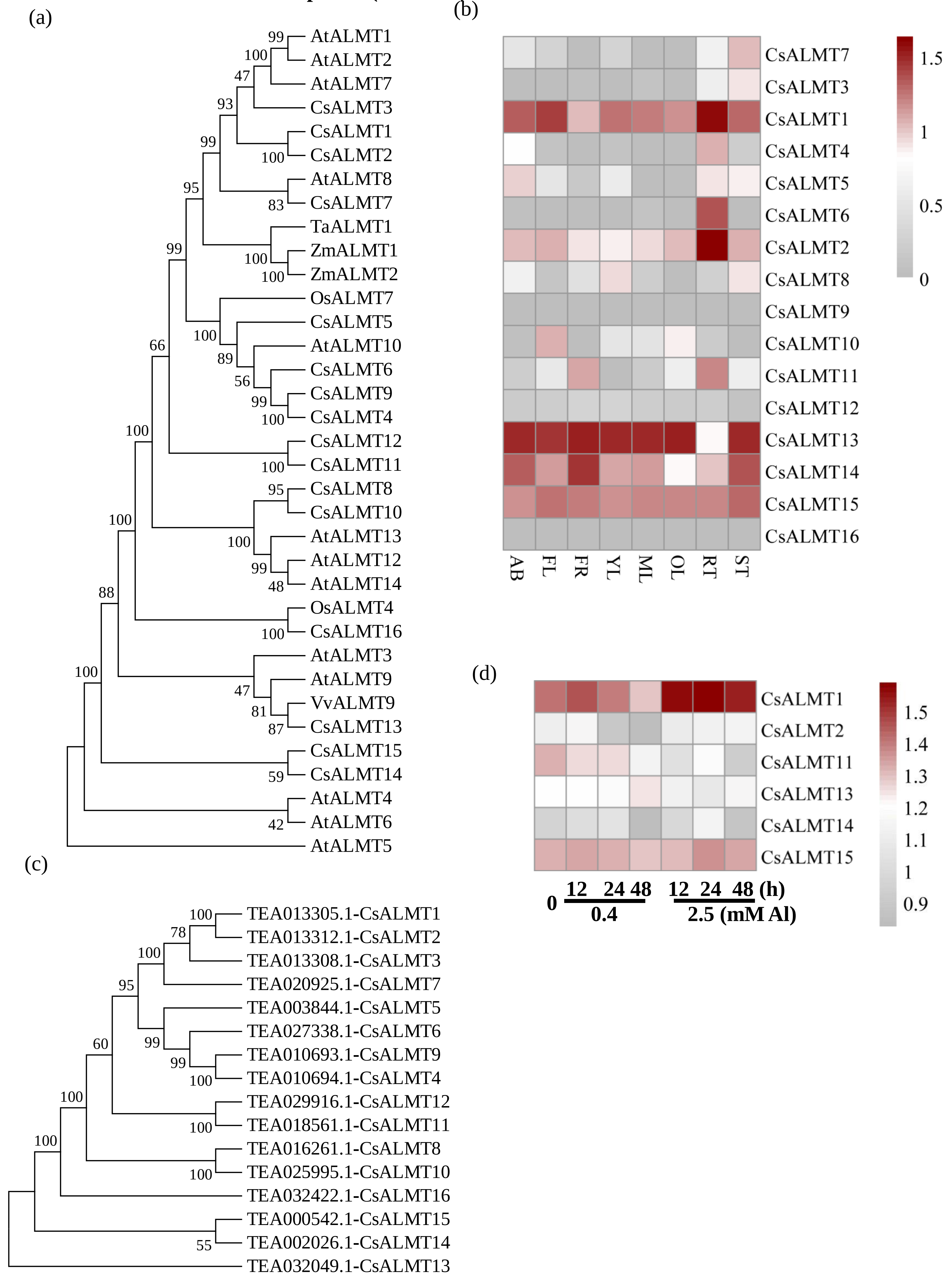

Fig S23 Identification and expression patterns of Aluminum-activated malate transporter (*CsALMT*) gene family in *C.sinensis*.
(a) Phylogenetic analysis of Aluminum-activated malate transporter (*CsALMT*) homology to functional characterized ones in Arabidopsis
(b) Annotation of Aluminum-activated malate transporter (*CsALMT*) genes in *C. sinensis*.
(c) Expression patterns of Aluminum-activated malate transporter (*CsALMT*) genes in various tissues of tea plants
(d) Expression patterns of Aluminum-activated malate transporter (*CsALMT*) genes in tea plant roots in response to Al stress for various times

Aluminum-activated citrate transporter (*CsMATE*)

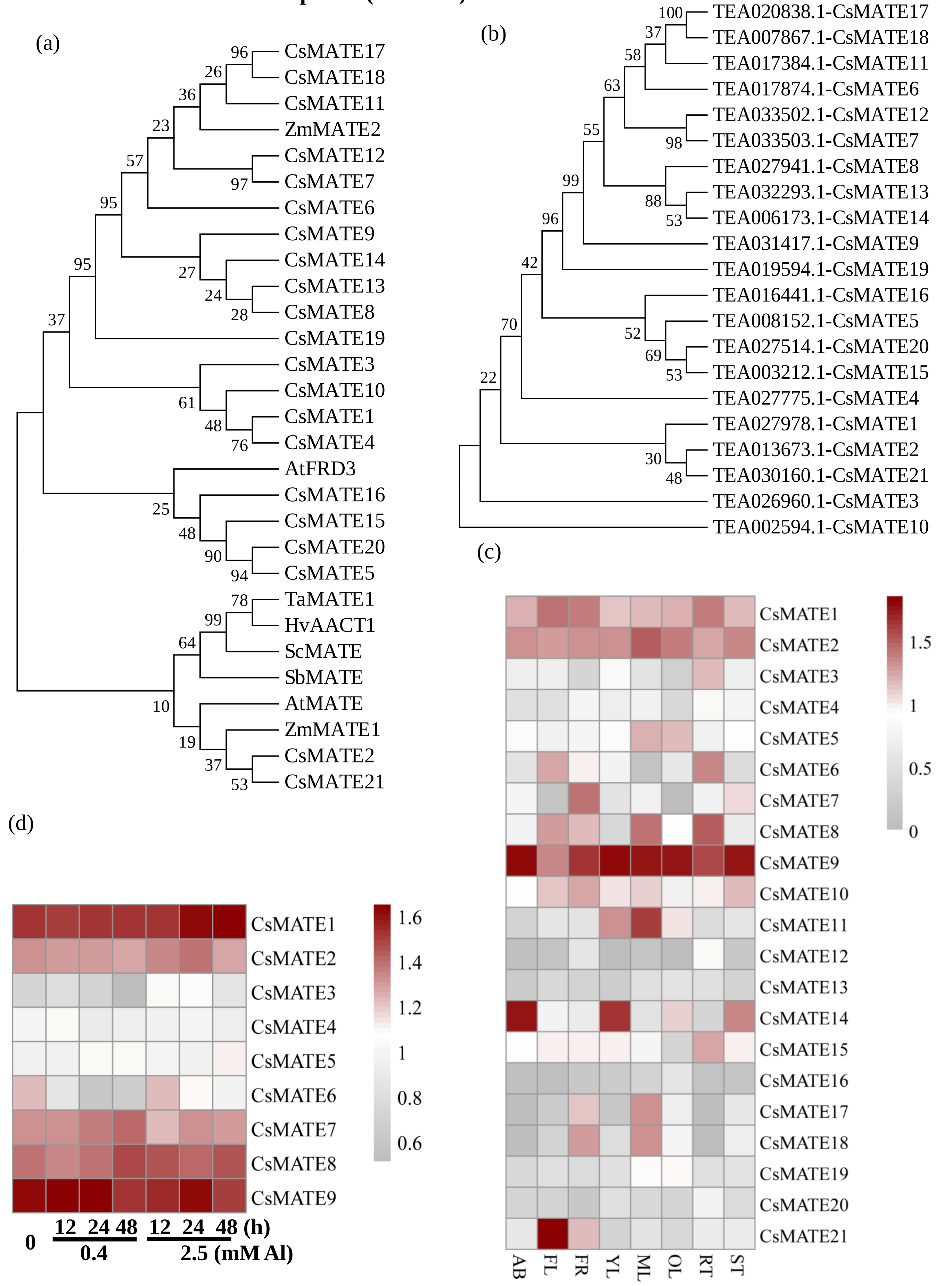

Fig S24 Identification and expression patterns of Aluminum-activated citrate transporter (*CsMATE*) gene family in *C.sinensis*.
(a) Phylogenetic analysis of Aluminum-activated citrate transporter (*CsMATE*) homology to functional characterized ones in Arabidopsis
(b) Annotation of Aluminum-activated citrate transporter (*CsMATE*) genes in *C. sinensis*.
(c) Expression patterns of Aluminum-activated citrate transporter (*CsMATE*) genes in various tissues of tea plants
(d) Expression patterns of Aluminum-activated citrate transporter (*CsMATE*) genes in tea plant roots in response to Al stress for various times
